# Supplementary material for: The configuration of Northern Hemisphere ice sheets through the Quaternary
Source: Nat Commun. 2019 Aug 16;10:3713. doi: 10.1038/s41467-019-11601-2 (PMC6697730; doi:10.1038/s41467-019-11601-2)
Supplement: Supplementary file 1 — Supplementary Information [file 41467_2019_11601_MOESM1_ESM.pdf]

Supplementary Information

The configuration of Northern Hemisphere ice sheets through the Quaternary

Batchelor *et al*.

This document contains details of the data sources used to inform our reconstructions of the maximum, minimum and best-estimate Northern Hemisphere (NH) ice-sheet extents. For each time-slice, the following are included:

- In Supplementary Figures, a raw data map showing the empirical and modelled data that were used to draw the ice-sheet reconstructions, alongside a map showing the hypothesised maximum, minimum and best-estimate ice-sheet extents. Supplementary Figure 1 shows the locations of the places mentioned in this document.
- In Supplementary Tables, a table listing the empirical and modelled data that were used to draw the ice-sheet reconstructions.
- In Supplementary Notes, explanatory text that details the decisions made in reconstructing the maximum, minimum and best-estimate ice-sheet extents. Overall and ice-sheet-wide robustness scores are also provided for each reconstruction. The robustness scores, which range from 0 (low) to 5 (high), are a subjective assessment of the amount and reliability of the source data from which the ice-sheet extent was constructed (Methods).

Shapefiles of our reconstructions, as well as the digitised and georeferenced empirical and modelled data, are available on the Open Science Framework [https://osf.io/7jen3/].

Table of Contents

Supplementary Figures *(reconstructions of NH ice-sheet extent at specified time slices)*

Supplementary Figure 1. Location map showing the places that are referred to in this document. 4

Supplementary Figure 2. 30 ka and 35 ka. 5

Supplementary Figure 3. 40 ka and 45 ka. 6

Supplementary Figure 4. MIS 4 (58–72 ka) and MIS 5a (72–86 ka). 7

Supplementary Figure 5. MIS 5b (86–92 ka) and MIS 5c (92–108 ka). 8

Supplementary Figure 6. MIS 5d (108–117 ka) and MIS 6 (132–190 ka). 9

Supplementary Figure 7. MIS 8 (243–279 ka) and MIS 10 (337–365 ka). 10

Supplementary Figure 8. MIS 12 (429–477 ka) and MIS 16 (622–677 ka). 11

Supplementary Figure 9. MIS 20–24 (790–928 ka) and the early Matuyama Chron (1.78–2.6 Ma). 12

Supplementary Figure 10. Late Gauss Chron (2.6–3.59 Ma). 13

Supplementary Tables (*published evidence for the spatial extent of NH glaciation at specified time slices*)

Supplementary Table 1. 30 ka. 14

Supplementary Table 2. 35 ka. 16

Supplementary Table 3. 40 ka 18

Supplementary Table 4. 45 ka. 20

Supplementary Table 5. MIS 4 22

Supplementary Table 6. MIS 5a. 25

Supplementary Table 7. MIS 5b.. 27

Supplementary Table 8. MIS 5c. 29

Supplementary Table 9. MIS 5d. 30

Supplementary Table 10. MIS 6. 32

Supplementary Table 11. MIS 8. 35

Supplementary Table 12. MIS 10. 36

Supplementary Table 13. MIS 12. 37

Supplementary Table 14. MIS 16. 38

Supplementary Table 15. MIS 20–24. 39

Supplementary Table 16. The early Matuyama Chron 40

Supplementary Table 17. The late Gauss Chron. 41

Supplementary Notes *(explanatory text that details the decisions made in reconstructing the maximum, minimum and best-estimate ice-sheet extents)*

Supplementary Note 1: The Last Glacial Maximum (LGM) 42

Supplementary Note 2: 30 ka 43

Supplementary Note 3: 35 ka 45

Supplementary Note 4: 40 ka 47

Supplementary Note 5: 45 ka 49

Supplementary Note 6: MIS 4 (58–72 ka) 51

Supplementary Note 7: MIS 5a (72–86 ka) 54

Supplementary Note 8: MIS 5b (86–92 ka) 56

Supplementary Note 9: MIS 5c (92–108 ka) 58

Supplementary Note 10: MIS 5d (108–117 ka) 60

Supplementary Note 11: MIS 6 (132–190 ka) 62

Supplementary Note 12: MIS 8 (243–279 ka) 64

Supplementary Note 13: MIS 10 (337–365 ka) 67

Supplementary Note 14: MIS 12 (429–477 ka) 70

Supplementary Note 15: MIS 16 (622–677 ka) 72

Supplementary Note 16: MIS 20–24 (790–928 ka) 74

Supplementary Note 17: Early Matuyama palaeomagnetic Chron (1.78–2.6 Ma) 76

Supplementary Note 18: Late Gauss palaeomagnetic Chron (2.6–3.59 Ma) 79

Supplementary References 82

Supplementary Figures

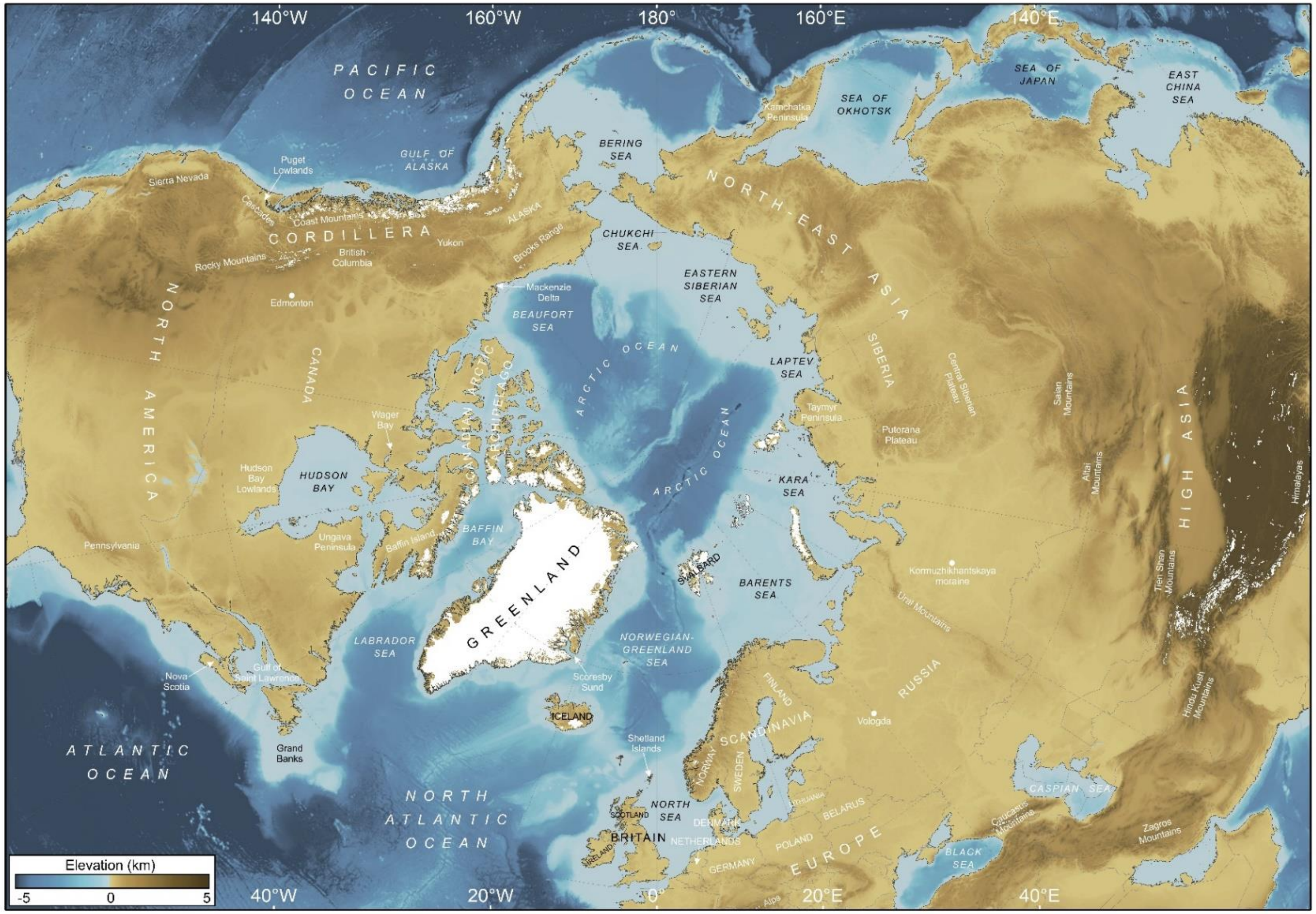

Supplementary Figure 1. Location map showing the places that are referred to in this document. Background is ETOPO1 1 arc-minute global relief model of Earth's surface[1].

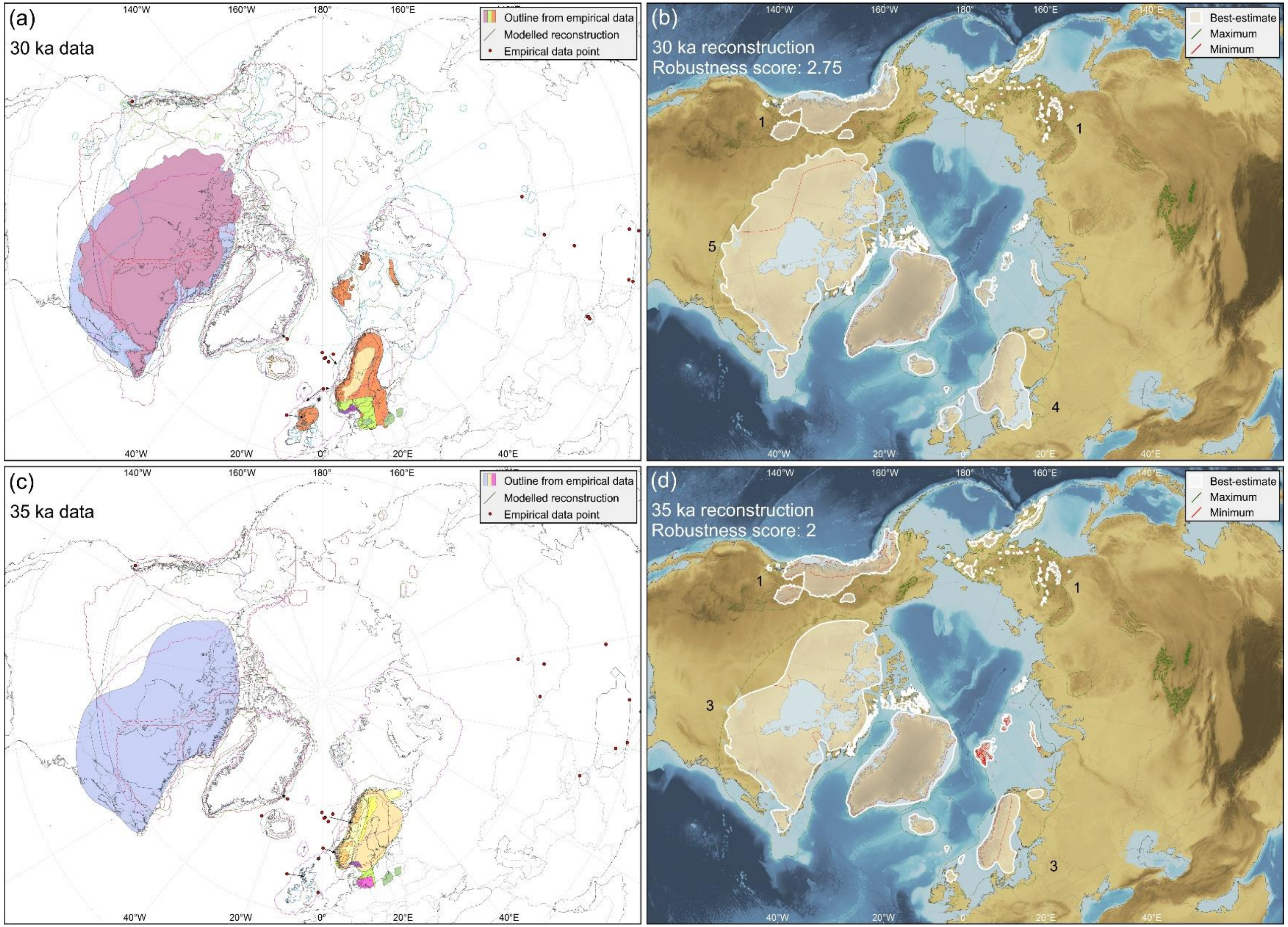

Supplementary Figure 2. Reconstructions of NH ice-sheet extent at 30 ka and 35 ka. **a,** compilation of previously published data on ice-sheet extent at 30 ka. **b,** maximum, minimum and best-estimate ice-sheet reconstruction for 30 ka. **c,** compilation of previously published data on ice-sheet extent at 35 ka. **d,** maximum, minimum and best-estimate ice-sheet reconstruction for 35 ka. Colours in **a** and **c** correspond with those in Supplementary Tables 1 and 2.

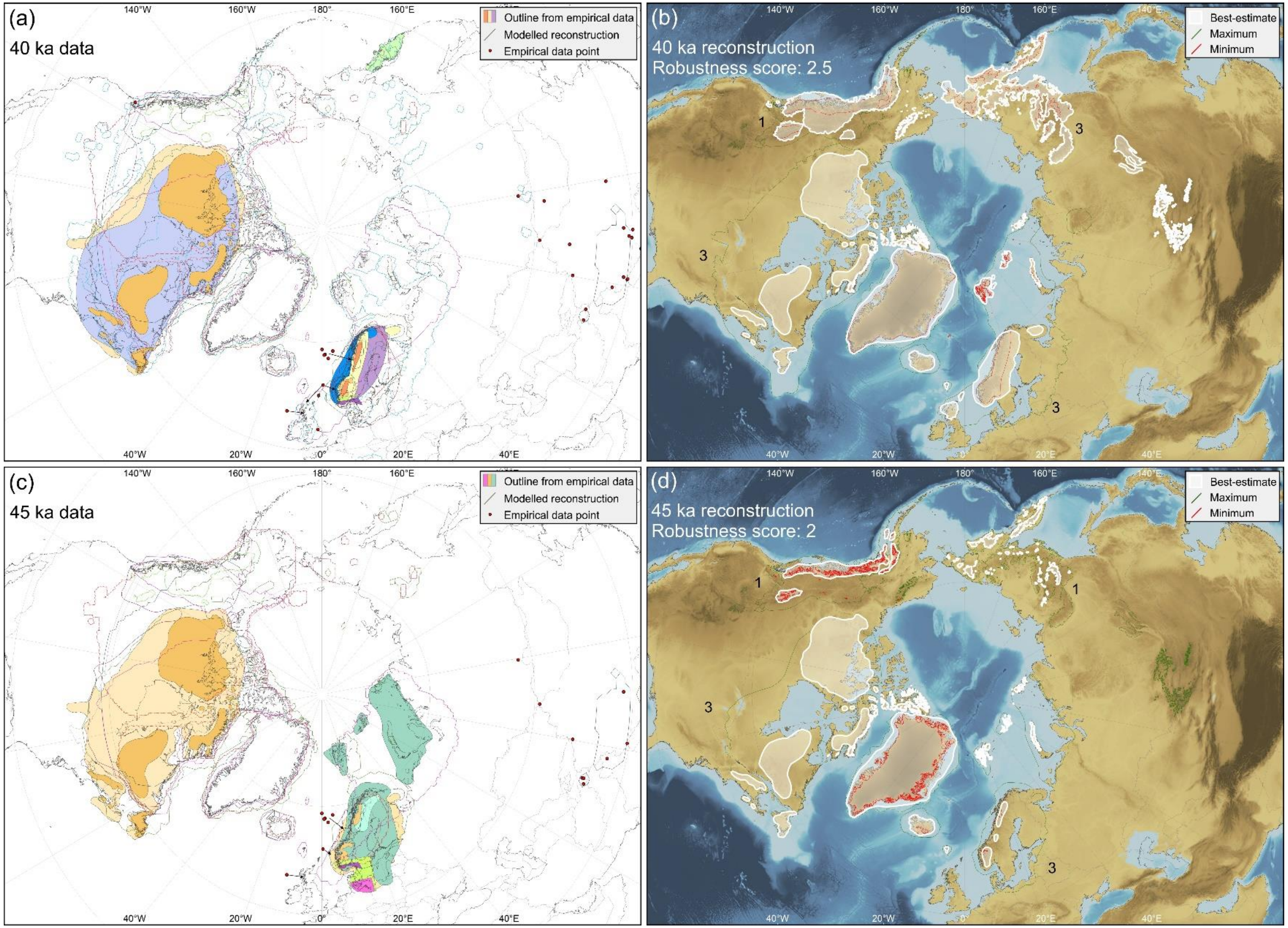

Supplementary Figure 3. Reconstructions of NH ice-sheet extent at 40 ka and 45 ka. See Supplementary Tables 3 and 4 for key. **a,** compilation of published data on ice-sheet extent at 40 ka. **b,** maximum, minimum and best-estimate ice-sheet reconstruction for 40 ka. **c,** compilation of previously published data on ice-sheet extent at 45 ka. **d,** maximum, minimum and best-estimate ice-sheet reconstruction for 45 ka.

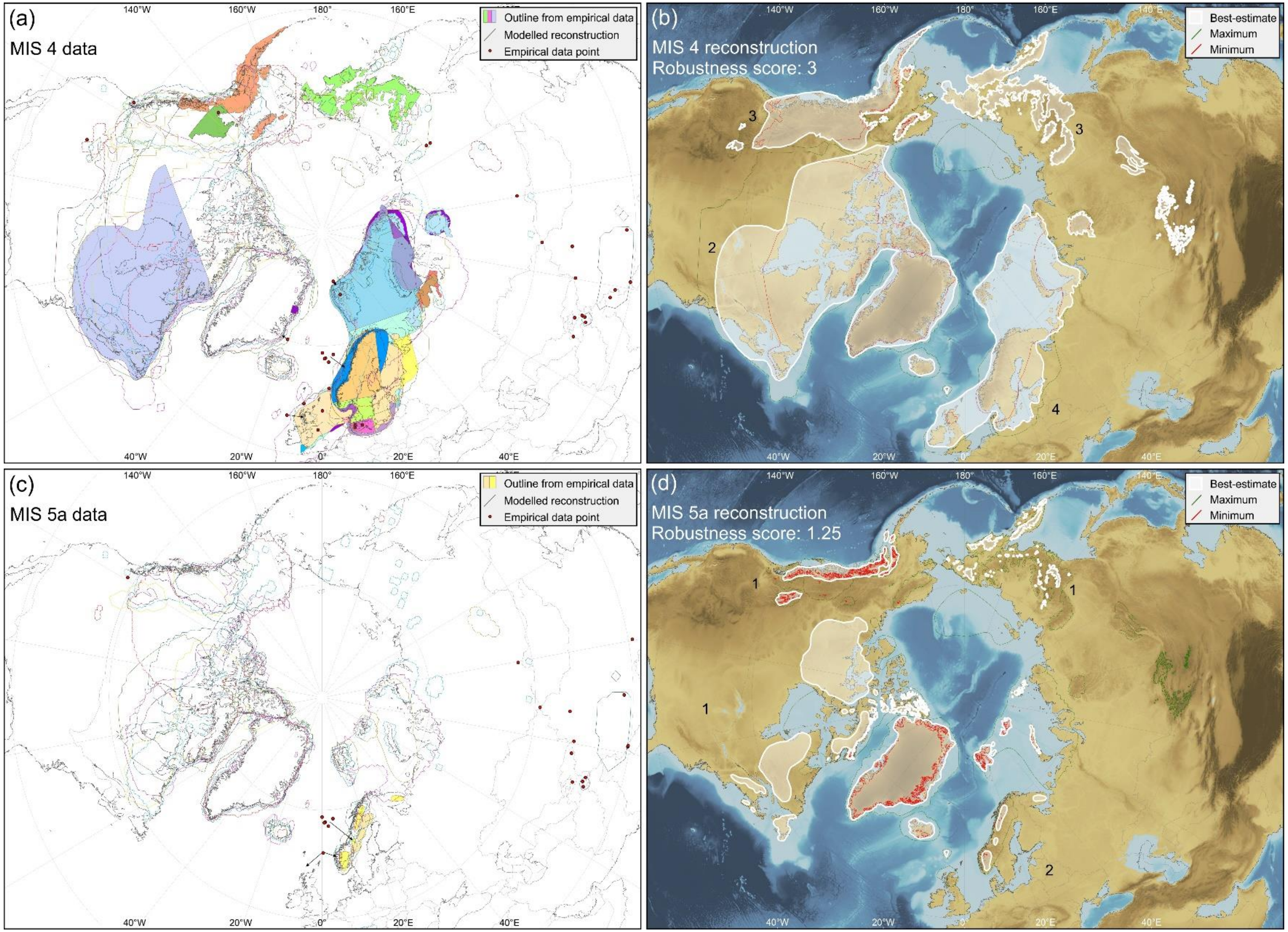

Supplementary Figure 4. Reconstructions of NH ice-sheet extent during MIS 4 (58–72 ka) and MIS 5a (72–86 ka). See Supplementary Tables 5 and 6 for key. **a,** compilation of previously published data on ice-sheet extent during MIS 4. **b,** maximum, minimum and best-estimate ice-sheet reconstruction for MIS 4. **c,** compilation of previously published data on ice-sheet extent during MIS 5a. **d,** maximum, minimum and best-estimate ice-sheet reconstruction for MIS 5a.

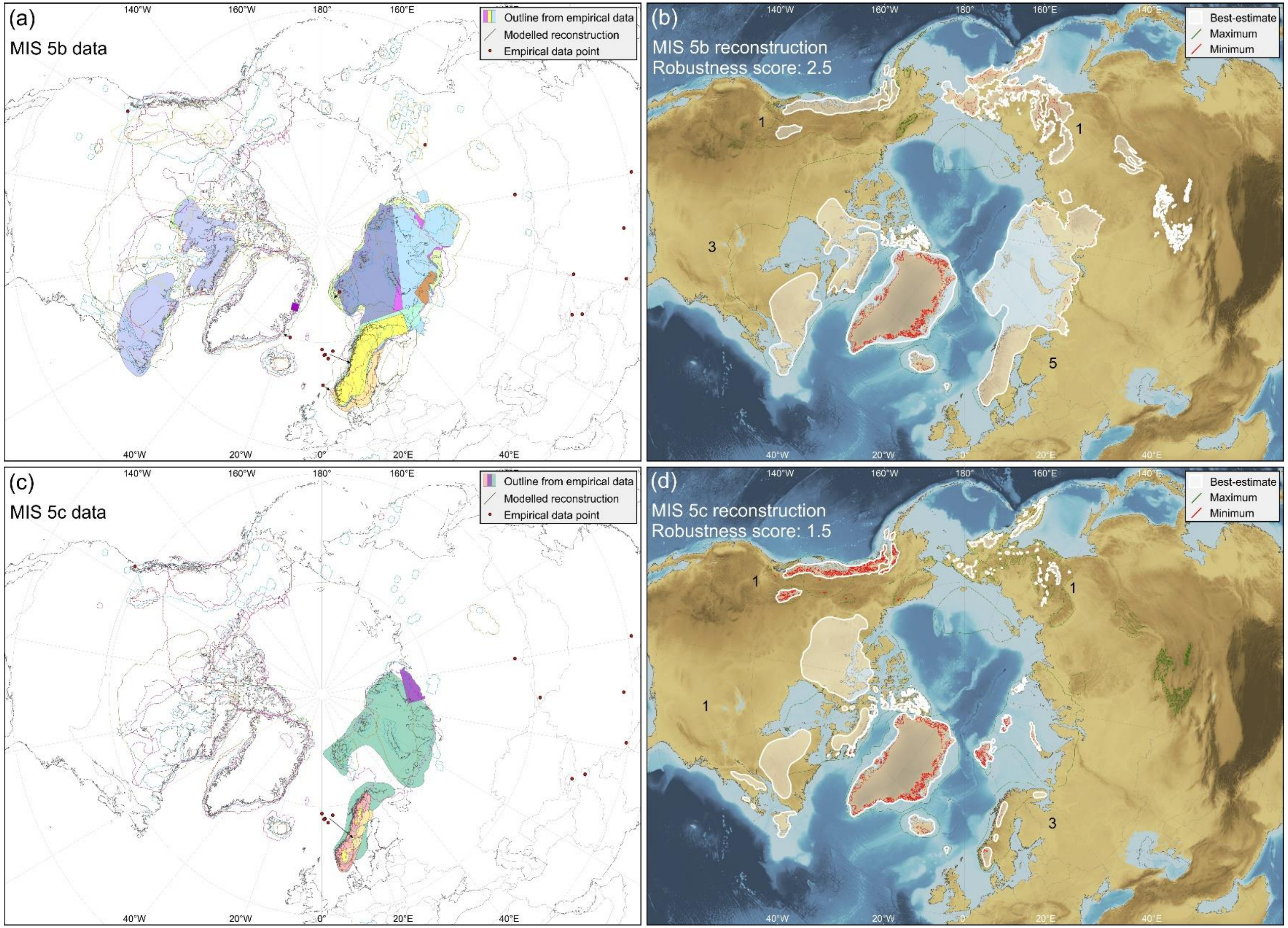

Supplementary Figure 5. Reconstructions of NH ice-sheet extent during MIS 5b (86–92 ka) and MIS 5c (92–108 ka). See Supplementary Tables 7 and 8 for key. **a,** compilation of previously published data on ice-sheet extent during MIS 5b. **b,** maximum, minimum and best-estimate ice-sheet reconstruction for MIS 5b. **c,** compilation of previously published data on ice-sheet extent during MIS 5c. **d,** maximum, minimum and best-estimate ice-sheet reconstruction for MIS 5c.

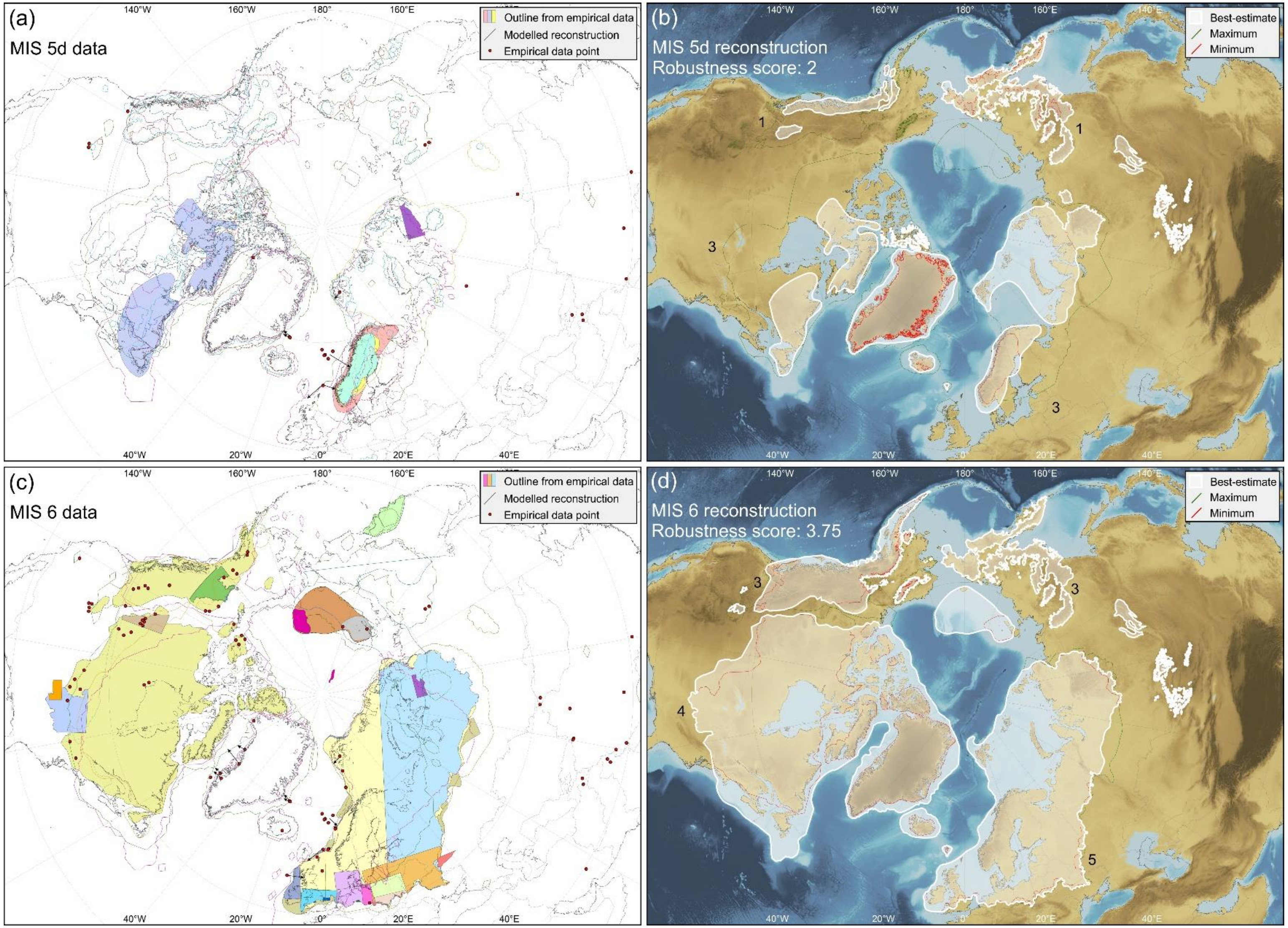

Supplementary Figure 6. Reconstructions of NH ice-sheet extent during MIS 5d (108–117 ka) and MIS 6 (132–190 ka). See Supplementary Tables 9 and 10 for key. **a,** compilation of previously published data on ice-sheet extent during MIS 5d. **b,** maximum, minimum and best-estimate ice-sheet reconstruction for MIS 5d. **c,** compilation of previously published data on ice-sheet extent during MIS 6. **d,** maximum, minimum and best-estimate ice-sheet reconstruction for MIS 6.

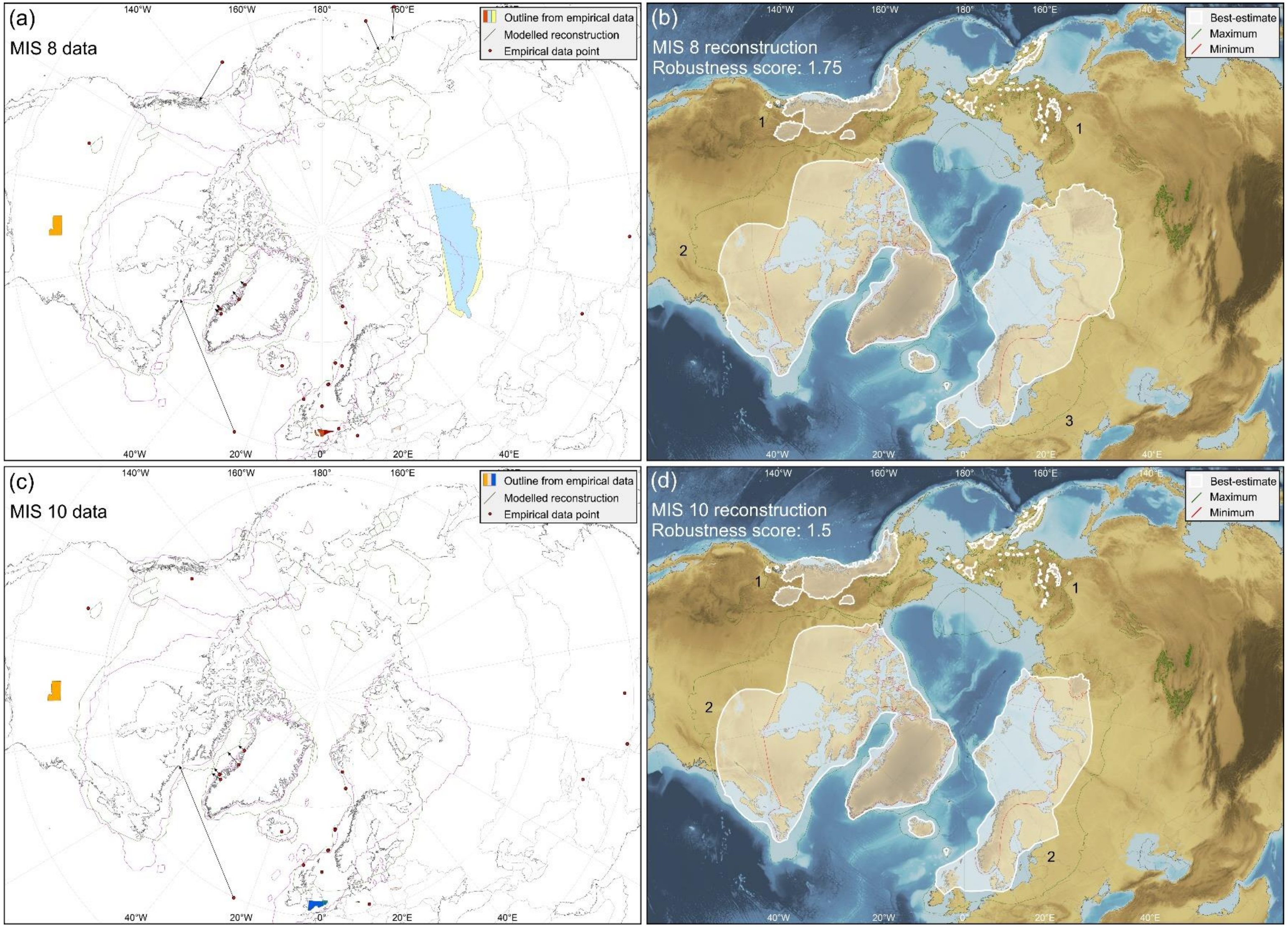

Supplementary Figure 7. Reconstructions of NH ice-sheet extent during MIS 8 (243–279 ka) and MIS 10 (337–365 ka). See Supplementary Tables 11 and 12 for key. **a,** compilation of previously published data on ice-sheet extent during MIS 8. **b,** maximum, minimum and best-estimate ice-sheet reconstruction for MIS 8. **c,** compilation of previously published data on ice-sheet extent during MIS 10. **d,** maximum, minimum and best-estimate ice-sheet reconstruction for MIS 10.

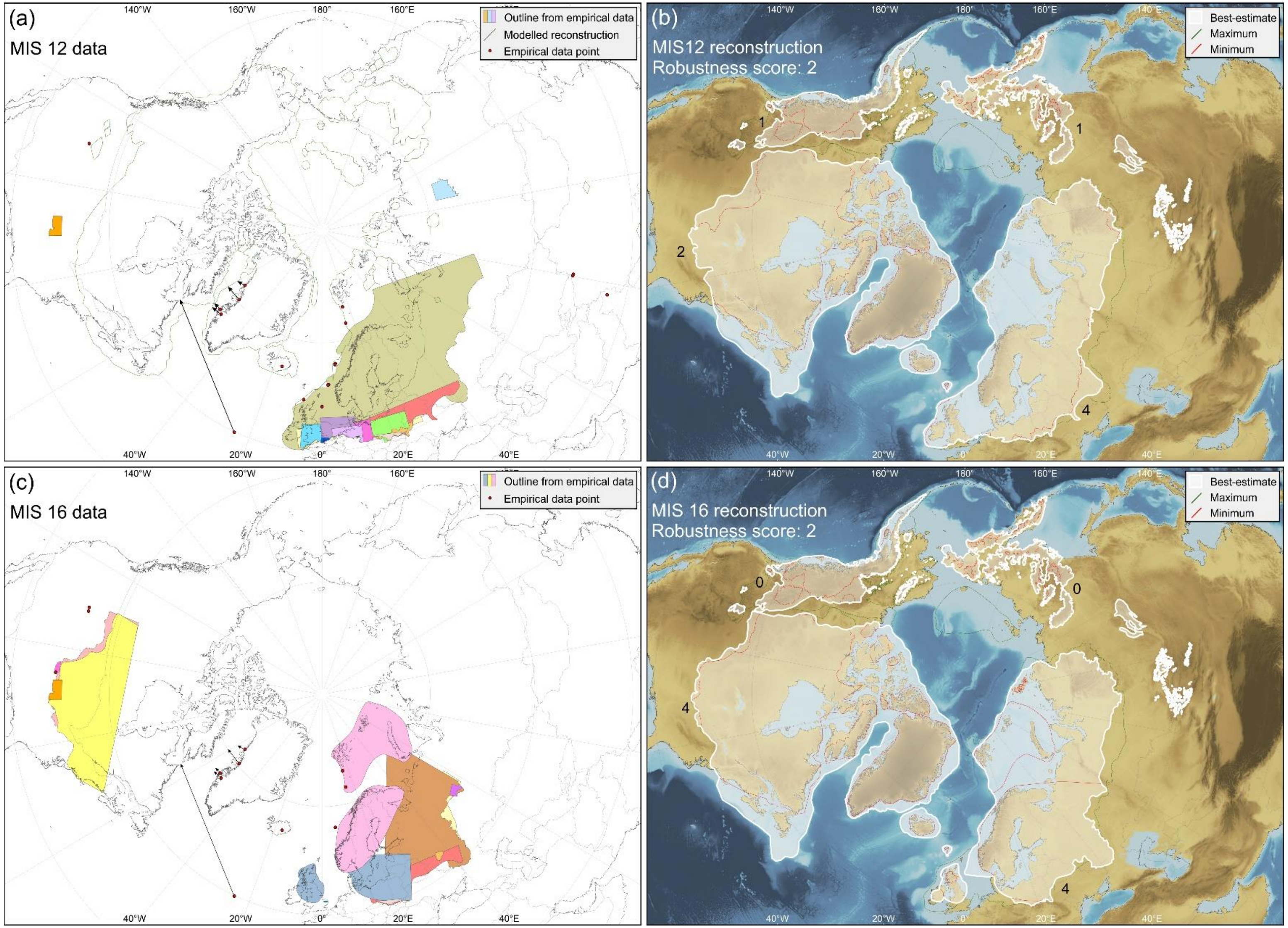

Supplementary Figure 8. Reconstructions of NH ice-sheet extent during MIS 12 (429–477 ka) and MIS 16 (622–677 ka). See Supplementary Tables 13 and 14 for key. **a,** compilation of previously published data on ice-sheet extent during MIS 12. **b,** maximum, minimum and best-estimate ice-sheet reconstruction for MIS 12. **c,** compilation of previously published data on ice-sheet extent during MIS 16. **d,** maximum, minimum and best-estimate ice-sheet reconstruction for MIS 16.

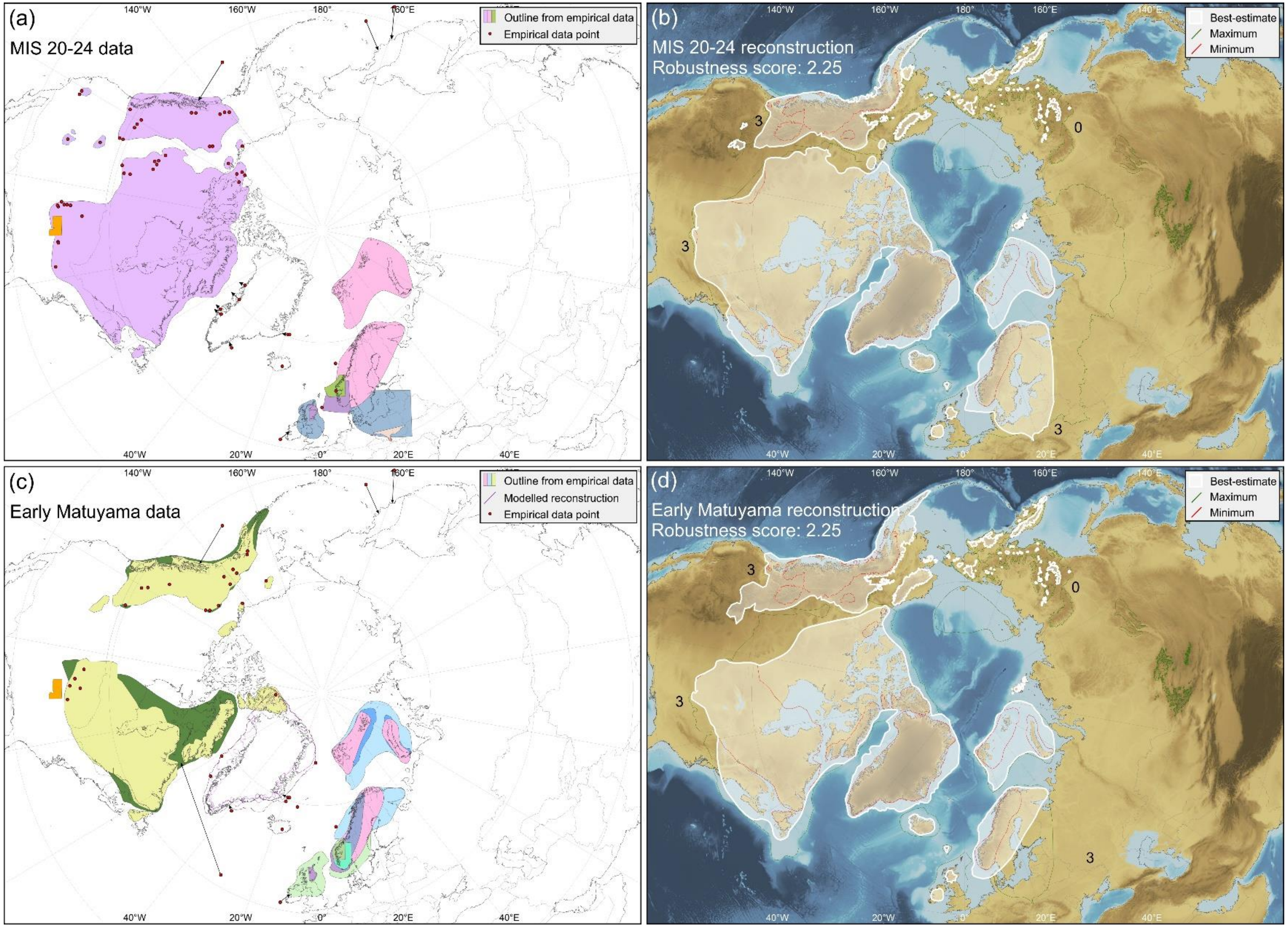

Supplementary Figure 9. Reconstructions of NH ice-sheet extent during MIS 20–24 (790–928 ka) and the early Matuyama magnetic Chron (1.78–2.6 Ma). See Supplementary Tables 15 and 16 for key. **a,** compilation of previously published data on ice-sheet extent during MIS 20–24. **b,** maximum, minimum and best-estimate ice-sheet reconstruction for MIS 20-24. **c,** compilation of previously published data on ice-sheet extent during the early Matuyama Chron. **d,** maximum, minimum and best-estimate ice-sheet reconstruction for the early Matuyama Chron.

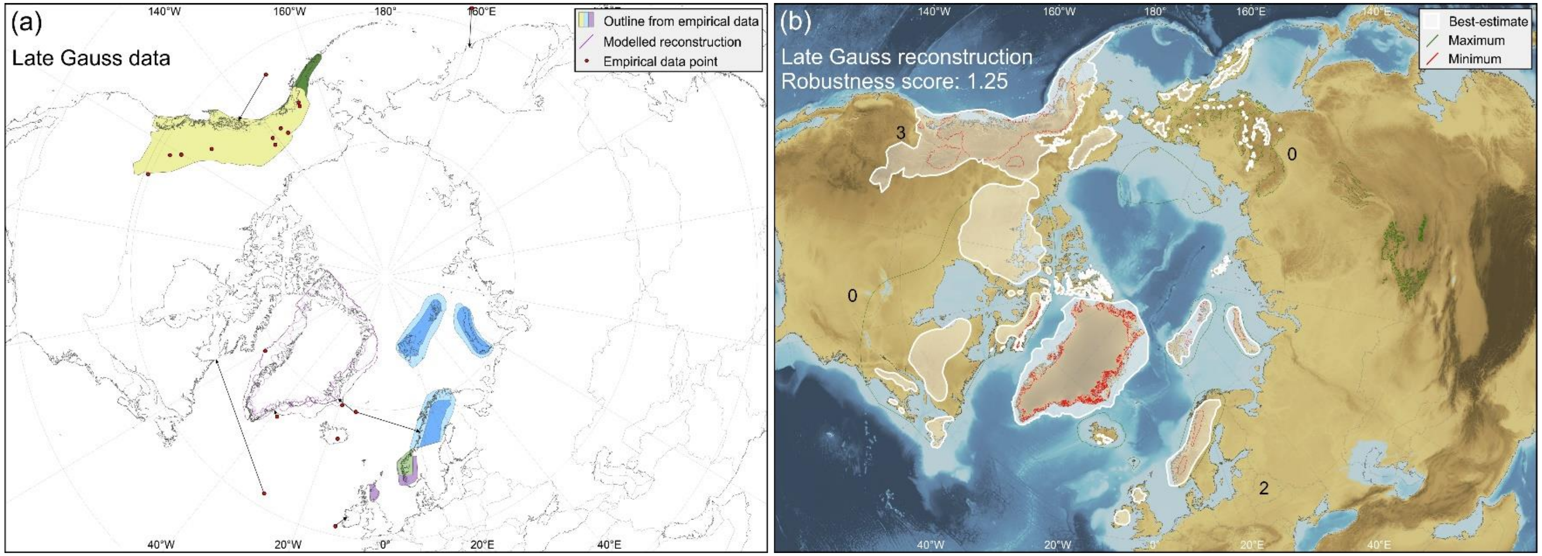

Supplementary Figure 10. Reconstructions of NH ice-sheet extent during the late Gauss magnetic Chron (2.6–3.59 Ma). See Supplementary Table 17 for key. **a**, compilation of previously published data on ice-sheet extent during the late Gauss Chron. **b**, maximum, minimum and best-estimate ice-sheet reconstruction for the late Gauss Chron.

Supplementary Tables

30 ka

Key	Reference	Data type	Details
	Dyke *et al.*, 2002[2]	Empirical outline	LIS; only main ice sheet shown
	Houmark-Nielsen, 2010[3]	Empirical outline	Western EIS; spans MIS 3
	Hughes *et al.*, 2016[4]	Empirical outline	EIS; 30–32 ka
	Kleman *et al.*, 2010[5]	Empirical outline	LIS; late MIS 3
	Larsen *et al.*, 2009[6]	Empirical outline	Western EIS; 29–30 ka
	Marks, 2012[7]	Empirical outline	EIS; tentative outlines for 29 ka and 33–37 ka
	Olsen *et al.*, 2013[8]	Empirical outline	Western EIS; 29–30 ka
	Bonelli *et al.*, 2009[9]	Model	NH; spans 0–126 ka with 1 ka increments. Model driven by variations in orbital parameters and CO_2 concentration.
	de Boer *et al.*, 2014[10]	Model	NH; spans 0–410 ka with 1 ka increments. Ice volume and temperature derived from benthic δ^{18}O stack.
	Ganopolski and Calov, 2011[11]	Model	NH; spans 0–800 ka with 1 ka increments. Model driven by variations in orbital parameters and CO_2 concentration.
	Heinemann *et al.*, 2014[12]	Model	NH; spans 0–78 ka with 1 ka increments. Model driven by variations in orbital parameters and CO_2 concentration.
	Hubbard *et al.*, 2009[13]	Model	Western EIS; timeslices from 35.95–11.65 ka. Model driven by NGRIP ice core δ^{18}O curve and sea-level reconstruction.
	Lambeck *et al.*, 2010[14]	Model	Western EIS. Model constructed using existing empirical data.
	Patton *et al.*, 2017[15]	Model	Iceland; 31 ka. Model driven by temperature, precipitation and sea-level perturbations.
	Seguinot *et al.*, 2016[16]	Model	CIS; 30 ka GRIP. Model driven by temperature offsets from proxy records and calibrated against existing empirical data.
	Stokes *et al.*, 2012[17]	Model	CIS and LIS. Model calibrated against existing empirical data.
	Zweck and Huybrechts, 2005[18]	Model	NH; 30–120 ka with 10 k increments. Model parameters chosen to match empirical LGM ice extent.
	Abramowski *et al.*, 2006[19]	Point-source	High Asia; ^{10}Be dating
	Arzhannikhov *et al.*, 2015[20]	Point-source	NE Asia; ^{10}Be dating
	Baumann *et al.*, 1995[21]	Glacial curve	Western EIS; IRD. 5 data points shown.
	Chevalier *et al.*, 2011[22]	Point-source	High Asia; ^{10}Be dating
	Hall, 2013[23]	Point-source	Western EIS; geomorphology suggests ice cap over Shetland Islands between 10 and 40 ka
	Hibbert *et al.*, 2010[24]	IRD	Western EIS

Key	Reference	Data type	Details
●	Lehmkuhl, 1998[25]	Point-source	High Asia; TL dating
●	Lekens *et al.*, 2009[26]	Glacial curve	Western EIS; based on seismic data and IRD
●	Li *et al.*, 2014[27]	Point-source	High Asia; ^{10}Be dating
●	Owen and Dortch, 2014[28]	Point-source	High Asia; TCN, OSL and ^{14}C dating. 2 data points shown.
●	Owen *et al.*, 2010[29]	Point-source	High Asia; ^{10}Be dating
●	Stein *et al.*, 1996[30]	IRD	East GIS; IRD, δ^{18}O and ^{14}C dating
●	Stübner *et al.*, 2017[31]	Point-source	High Asia; ^{10}Be dating
●	Thackray, 2008[32]	Point-source	LIS; ^{14}C and ^{36}Cl dating

Supplementary Table 1. Published evidence for the spatial extent of Northern Hemisphere (NH) glaciation at 30 ka. IRD = ice-rafted debris; OSL = optically-stimulated luminescence; TCN = terrestrial cosmogenic nuclide; TL = thermoluminescence. Key corresponds with colours in Supplementary Figure 2a.

35 ka

Key	Reference	Data type	Details
	Arnold *et al.*, 2002[33]	Empirical outline	Western EIS; minimum extent during MIS 3
	Houmark-Nielsen, 2010[3]	Empirical outline	Western EIS; spans MIS 3
	Hughes *et al.*, 2016[4]	Empirical outline	EIS; 34–38 ka; min and max versions
	Kleman *et al.*, 2010[5]	Empirical outline	LIS
	Larsen *et al.*, 2009[6]	Empirical outline	Western EIS; 30–50 ka
	Mangerud *et al.*, 2011[34]	Empirical outline	Northern EIS; 35–38 ka
	Marks, 2012[7]	Empirical outline	EIS; tentative outline for 33–37 ka
	Obst *et al.*, 2017[35]	Empirical outline	EIS; 30–34k
	Olsen *et al.*, 2013[8]	Empirical outline	Western EIS; 33 ka
	Arnold *et al.*, 2002[33]	Model	Western EIS; 36 ka. Model driven by GISP $\delta^{18}O$ ice core record.
	Bonelli *et al.*, 2009[9]	Model	NH; spans 0–126 ka with 1 ka increments. Model driven by variations in orbital parameters and CO_2 concentration.
	de Boer *et al.*, 2014[10]	Model	NH; spans 0–410 ka with 1 ka increments. Ice volume and temperature derived from benthic $\delta^{18}O$ stack.
	Ganopolski and Calov, 2011[11]	Model	NH; spans 0–800 ka with 1 ka increments. Model driven by variations in orbital parameters and CO_2 concentration.
	Heinemann *et al.*, 2014[12]	Model	NH; spans 0–78 ka with 1 ka increments. Model driven by variations in orbital parameters and CO_2 concentration.
	Hubbard *et al.*, 2009[13]	Model	Western EIS; timeslices from 11.65–35.95 ka. Model driven by NGRIP ice core $\delta^{18}O$ curve and sea-level reconstruction.
	Lambeck *et al.*, 2010[14]	Model	Western EIS. Model constructed using existing empirical data.
●	Abramowski *et al.*, 2006[19]	Point-source	High Asia; ^{10}Be dating
●	Arzhannikhov *et al.*, 2015[20]	Point-source	NE Asia; ^{10}Be dating
●	Baumann *et al.*, 1995[21]	Glacial curve	Western EIS; IRD. 5 data points shown.
●	Chevalier *et al.*, 2011[22]	Point-source	High Asia; ^{10}Be dating
●	Hall, 2013[23]	Point-source	Western EIS; geomorphology suggests ice cap over Shetland Islands between 10 and 40 ka
●	Hibbert *et al.*, 2010[24]	IRD	Western EIS
●	Lehmkuhl, 1998[25]	Point-source	High Asia; TL dating
●	Lekens *et al.*, 2009[26]	Glacial curve	Western EIS; based on seismic data and IRD
●	Murton, 2017[36]	Point-source	Western EIS; OSL and palaeomagnetic dating
●	Owen *et al.*, 2003[37]	Point-source	High Asia; TCN and TL dating
●	Owen *et al.*, 2009[38]	Point-source	High Asia; TCN and OSL dating
●	Owen *et al.*, 2010[29]	Point-source	High Asia; ^{10}Be dating

Key	Reference	Data type	Details
●	Rother *et al.*, 2014[39]	Point-source	NE Asia; TCN dating
●	Stein *et al.*, 1996[30]	IRD	East GIS; IRD, $\delta^{18}O$ and ^{14}C dating.
●	Syvitski *et al.*, 1999[40]	Seismic data	Iceland; seismic data
●	Thackray, 2008[41]	Point-source	LIS; ^{14}C and ^{36}Cl dating

Supplementary Table 2. Published evidence for the spatial extent of NH glaciation at 35 ka. Key corresponds with colours in Supplementary Figure 2c.

40 ka

Key	Reference	Data type	Details
	Arnold *et al.*, 2002[33]	Empirical outline	Western EIS; minimum extent during MIS 3
	Barr and Solomina, 2015[41]	Empirical outline	NE Asia
	Dredge and Thorleifson, 1987[42]	Empirical outline	LIS; hypotheses for MIS 3
	Houmark-Nielsen, 2010[3]	Empirical outline	Western EIS; spans MIS 3
	Hughes *et al.*, 2016[4]	Empirical outline	EIS; 34–38 ka; min and max versions
	Kleman *et al.*, 2010[5]	Empirical outline	LIS
	Larsen *et al.*, 2009[6]	Empirical outline	Western EIS; 30–50 ka
	van Andel and Tzedakis, 1996[43]	Empirical outline	EIS; min and max versions
	Arnold *et al.*, 2002[33]	Model	Western EIS; 36 ka. Model driven by GISP $\delta^{18}O$ ice core record.
	Bonelli *et al.*, 2009[9]	Model	NH; spans 0–126 ka with 1 ka increments. Model driven by variations in orbital parameters and CO_2 concentration.
	de Boer *et al.*, 2014[10]	Model	NH; spans 0–410 ka with 1 ka increments. Ice volume and temperature derived from benthic $\delta^{18}O$ stack.
	Ganopolski and Calov, 2011[11]	Model	NH; spans 0–800 ka with 1 ka increments. Model driven by variations in orbital parameters and CO_2 concentration.
	Heinemann *et al.*, 2014[12]	Model	NH; spans 0–78 ka with 1 ka increments. Model driven by variations in orbital parameters and CO_2 concentration.
	Lambeck *et al.*, 2010[14]	Model	Western EIS. Model constructed using existing empirical data.
	Marshall *et al.*, 2000[44]	Model	LIS and CIS. Model driven by GRIP $\delta^{18}O$ ice core record and general circulation model.
	Seguinot *et al.*, 2016[16]	Model	CIS; 42.9 ka. Model driven by temperature offsets from proxy records and calibrated against existing empirical data.
	Stokes *et al.*, 2012[17]	Model	LIS and CIS. Model calibrated against existing empirical data.
	Zweck and Huybrechts, 2005[18]	Model	NH; 30–120 ka with 10 ka increments. Model parameters chosen to match empirical LGM ice extent.
●	Abramowski *et al.*, 2006[19]	Point-source	High Asia; ^{10}Be dating
●	Arzhannikhov *et al.*, 2015[20]	Point-source	NE Asia; ^{10}Be dating
●	Baumann *et al.*, 1995[21]	Glacial curve	Western EIS; IRD. 5 data points shown.
●	Chevalier *et al.*, 2011[22]	Point-source	High Asia; ^{10}Be dating
●	Hall, 2013[23]	Point-source	Western EIS; geomorphology suggests ice cap over Shetland Islands between 10 and 40 ka

Key	Reference	Data type	Details
●	Hibbert *et al*., 2010[24]	IRD	Western EIS
●	Lekens *et al*., 2009[26]	Glacial curve	Western EIS; based on seismic data and IRD
●	Li *et al*., 2014[27]	Point-source	High Asia; ^{10}Be dating
●	Murton, 2017[36]	Point-source	Western EIS; OSL and palaeomagnetic dating
●	Owen and Dortch, 2014[28]	Point-source	High Asia; TCN, OSL and ^{14}C dating
●	Owen *et al*., 2003[37]	Point-source	High Asia; TCN and TL dating
●	Owen *et al*., 2009[38]	Point-source	High Asia; TCN and OSL dating
●	Owen *et al*., 2010[29]	Point-source	High Asia; ^{10}Be dating
●	Rother *et al*., 2014[39]	Point-source	NE Asia; TCN dating
●	Stübner *et al*., 2017[31]	Point-source	High Asia; ^{10}Be dating
●	Thackray, 2008[32]	Point-source	LIS; ^{14}C and ^{36}Cl dating
●	Zhao *et al*., 2009[45]	Point-source	High Asia; ESR dating
●	Zhao *et al*., 2013[46]	Point-source	High Asia; ESR and OSL dating

Supplementary Table 3. Published evidence for the spatial extent of NH glaciation at 40 ka. ERS = electron spin resonance. Key corresponds with colours in Supplementary Figure 3a.

45 ka

Key	Reference	Data type	Details
	Arnold *et al.*, 2002[33]	Empirical outline	Western EIS; minimum extent during MIS 3
	Dredge and Thorleifson, 1987[42]	Empirical outline	LIS; hypotheses for MIS 3
	Helmens, 2014[47]	Empirical outline	Western EIS; early MIS 3
	Houmark-Nielsen, 2010[3]	Empirical outline	Western EIS; spans MIS 3
	Larsen *et al.*, 2006[46]	Empirical outline	EIS; 45–55 ka
	Larsen *et al.*, 2009[6]	Empirical outline	Western EIS; 30–50 ka
	Obst *et al.*, 2017[35]	Empirical outline	Western EIS; 46–56 ka
	Olsen *et al.*, 2013[8]	Empirical outline	Western EIS; 44 ka
	Arnold *et al.*, 2002[31]	Model	Western EIS; 36 ka. Model driven by GISP $\delta^{18}O$ ice core record.
	Bonelli *et al.*, 2009[9]	Model	NH; spans 0–126 ka with 1 ka increments. Model driven by variations in orbital parameters and CO_2 concentration.
	de Boer *et al.*, 2014[10]	Model	NH; spans 0–410 ka with 1 ka increments. Ice volume and temperature derived from benthic $\delta^{18}O$ stack.
	Ganopolski and Calov, 2011[11]	Model	NH; spans 0–800 ka with 1 ka increments. Model driven by variations in orbital parameters and CO_2 concentration.
	Heinemann *et al.*, 2014[12]	Model	NH; spans 0–78 ka with 1 ka increments. Model driven by variations in orbital parameters and CO_2 concentration.
	Lambeck *et al.*, 2010[14]	Model	Western EIS. Model constructed using existing empirical data.
	Seguinot *et al.*, 2016[16]	Model	CIS; 45.9 ka. Model driven by temperature offsets from proxy records and calibrated against existing empirical data.
	Stokes *et al.*, 2012[17]	Model	LIS and CIS. Model calibrated against existing empirical data.
●	Abramowski *et al.*, 2006[19]	Point-source	High Asia; ^{10}Be dating
●	Arzhannikhov *et al.*, 2015[20]	Point-source	NE Asia; ^{10}Be dating
●	Baumann *et al.*, 1995[21]	Glacial curve	Western Norway; IRD. 5 data points shown.
●	Hibbert *et al.*, 2010[24]	IRD	Western EIS
●	Lekens *et al.*, 2009[26]	Glacial curve	Western EIS; based on seismic data and IRD
●	Owen and Dortch, 2014[28]	Point-source	High Asia; TCN, OSL and ^{14}C dating. 2 data points shown.
●	Owen *et al.*, 2006[49]	Point-source	High Asia; TCN dating
●	Owen *et al.*, 2010[29]	Point-source	High Asia; ^{10}Be dating
●	Stübner *et al.*, 2017[31]	Point-source	High Asia; ^{10}Be dating
●	Zhao *et al.*, 2009[45]	Point-source	High Asia; ESR dating
●	Zhao *et al.*, 2013[46]	Point-source	High Asia; ESR and OSL dating

Supplementary Table 4. Published evidence for the spatial extent of NH glaciation at 45 ka. IRSL = infrared stimulated luminescence. Key corresponds with colours in Supplementary Figure 3c.

MIS 4 (58–72 ka)

Key	Reference	Data type	Details
	Arnold *et al.*, 2002[33]	Empirical outline	Western EIS
	Astakhov, 2018[52]	Empirical outline	Eastern EIS; 70–90 ka
	Astakhov *et al.*, 2016[53]	Empirical outline	Eastern EIS; 50–60 ka
	Carr *et al.*, 2006[54]	Empirical outline	Western EIS; tentative extent
	Glushkova, 2011[55]	Empirical outline	NE Asia
	Helmens, 2014[47]	Empirical outline	EIS
	Hjort, 1981[56]	Empirical outline	Northeast GIS; tentative glacial limits
	Houmark-Nielsen, 2010[3]	Empirical outline	Western EIS; 46–56 ka
	Kaufman *et al.*, 2011[57]	Empirical outline	CIS; outline may be MIS 6 in places
	Kleman *et al.*, 2010[5]	Empirical outline	LIS; includes extrapolations based on topography
	Kleman *et al.*, 2013[58]	Empirical outline	EIS
	Larsen *et al.*, 2006[48]	Empirical outline	EIS; 65–70 ka
	Larsen *et al.*, 2009[6]	Empirical outline	Western EIS; 50–55 ka
	Lundqvist, 2004[59]	Empirical outline	Western EIS; 65 ka
	Mangerud *et al.*, 2011[34]	Empirical outline	Western EIS; 55–60 ka
	Möller *et al.*, 2015[60]	Empirical outline	Eastern EIS
	Obst *et al.*, 2017[35]	Empirical outline	Western EIS; 46–56 ka
	Olsen *et al.*, 2013[8]	Empirical outline	Western EIS; 65 ka
	Rolfe *et al.*, 2012[61]	Empirical outline	Western EIS; tentative extent
	Svendsen *et al.*, 2004[62]	Empirical outline	EIS
	Svendsen *et al.*, 2014[63]	Empirical outline	Eastern EIS
	Turner *et al.*, 2016[64]	Empirical outline	CIS; MIS 4 and MIS 6 extents not differentiated
	van Andel and Tzedakis, 1996[43]	Empirical outline	Western EIS; includes min and max versions
	Bonelli *et al.*, 2009[9]	Model	NH; spans 0–126 ka with 1 ka increments. Model driven by variations in orbital parameters and CO_2 concentration.
	de Boer *et al.*, 2014[10]	Model	NH; spans 0–410 ka with 1 ka increments. Ice volume and temperature derived from benthic $\delta^{18}O$ stack.
	Ganopolski and Calov, 2011[11]	Model	NH; spans 0–800 ka with 1 ka increments. Model driven by variations in orbital parameters and CO_2 concentration.
	Heinemann *et al.*, 2014[12]	Model	NH; spans 0–78 ka with 1 ka increments. Model driven by variations in orbital parameters and CO_2 concentration.
	Kleman *et al.*, 2002[65]	Model	LIS and CIS; 70 ka. Model driven by GRIP $\delta^{18}O$ ice core record and tuned to fit existing empirical data.
	Kleman *et al.*, 2013[58]	Model	NH; 64 ka. Model constrained by existing empirical data.
	Lambeck *et al.*, 2010[14]	Model	Western EIS. Model constructed using existing empirical data.

Key	Reference	Data type	Details
	Marshall *et al*., 2000[44]	Model	LIS and CIS; 60 ka. Model driven by GRIP δ^{18}O ice core record and general circulation model.
	Seguinot *et al*., 2016[16]	Model	CIS; 60 ka. Model driven by temperature offsets from proxy records and calibrated against existing empirical data.
	Stokes *et al*., 2012[17]	Model	CIS and LIS. Model calibrated against existing empirical data.
	Zweck and Huybrechts, 2005[18]	Model	NH; 30–120 ka with 10 ka increments. Model parameters chosen to match empirical LGM ice extent.
●	Abramowski *et al*., 2006[19]	Point-source	High Asia; ^{10}Be dating
●	Arzhannikhov *et al*., 2015[20]	Point-source	NE Asia; ^{10}Be dating
●	Baumann *et al*., 1995[21]	Glacial curve	Western EIS; IRD. 5 data points shown.
●	Chevalier *et al*., 2011[22]	Point-source	High Asia; ^{10}Be dating
●	Davies, 2008[66]	Point-source	Western EIS; OSL dating
●	Eccleshall *et al*., 2016[67]	Glacial curve	EIS; OSL dating
●	Grin *et al*., 2016[68]	Point-source	High Asia; overview of regional glaciations
●	Hall and Shroba, 1995[69]	Point-source	US mountains; soil properties
●	Hibbert *et al*., 2010[24]	IRD	Western EIS
●	Houmark-Nielsen, 2010[3]	Point-source	Western EIS; OSL and ^{14}C dating. 3 data points shown.
●	Li *et al*., 2014[27]	Point-source	High Asia; ^{10}Be dating
●	Owen and Dortch, 2014[28]	Point-source	High Asia; TCN, OSL and ^{14}C dating. 2 data points shown.
●	Owen *et al*., 2006[49]	Point-source	High Asia; TCN dating
●	Owen *et al*., 2010[29]	Point-source	High Asia; ^{10}Be dating
●	Sejrup *et al*., 2000[70]	Glacial curve	Western EIS; from seismic data
●	Sejrup *et al*., 2005[71]	Glacial curve	Western EIS; glacial curves from seismic profiles
●	Stauch and Lehmkuhl, 2010[50]	Point-source	NE Asia; IRSL dating
●	Stein *et al*., 1996[30]	IRD	East GIS; IRD, δ^{18}O and ^{14}C dating.
●	Stewart and Lonergan, 2011[72]	Seismic data	Western EIS; seismic data
●	Stübner *et al*., 2017[31]	Point-source	High Asia; ^{10}Be dating
●	Thackray, 2008[32]	Point-source	LIS; ^{14}C and ^{36}Cl dating
●	Ward *et al*., 2007[51]	Point-source	CIS; TCN dating
●	Winkelmann *et al*., 2008[73]	Glacial curve	EIS; based on IRD
●	Zech *et al*., 2011[74]	Point-source	NE Asia; IRSL dating
●	Zech *et al*., 2013[75]	Point-source	High Asia; ^{10}Be dating
●	Zhao *et al*., 2009[45]	Point-source	High Asia; ERS dating
●	Zhao *et al*., 2013[46]	Point-source	High Asia; ERS and OSL dating

Supplementary Table 5. Published evidence for the spatial extent of NH glaciation during MIS 4. Key corresponds with colours in Supplementary Figure 4a.

MIS 5a (72–86 ka)

Key	Reference	Data type	Details
	Mangerud *et al.*, 2011[34]	Empirical outline	Western EIS; Odderade interstadial, 80 ka
	Olsen *et al.*, 2013[8]	Empirical outline	Western EIS; 80 ka
	Bonelli *et al.*, 2009[9]	Model	NH; spans 0–126 ka with 1 ka increments. Model driven by variations in orbital parameters and CO_2 concentration.
	de Boer *et al.*, 2014[10]	Model	NH; spans 0–410 ka with 1 ka increments. Ice volume and temperature derived from benthic $\delta^{18}O$ stack.
	Ganopolski and Calov, 2011[11]	Model	NH; spans 0–800 ka with 1 ka increments. Model driven by variations in orbital parameters and CO_2 concentration.
	Heinemann *et al.*, 2014[12]	Model	NH; spans 0–78 ka with 1 ka increments. Model driven by variations in orbital parameters and CO_2 concentration.
	Kleman *et al.*, 2002[65]	Model	LIS and CIS; 84 ka. Model driven by GRIP $\delta^{18}O$ ice core record and tuned to fit existing empirical data.
	Lambeck *et al.*, 2006[76]	Model	Western EIS; 85 ka. Model constructed using existing empirical data.
	Marshall *et al.*, 2000[44]	Model	LIS and CIS; 80 ka. Model driven by GRIP $\delta^{18}O$ ice core record and general circulation model.
	Stokes *et al.*, 2012[17]	Model	CIS and LIS; 80 ka. Model calibrated against existing empirical data.
	Zweck and Huybrechts, 2005[18]	Model	NH; 30–120 ka with 10 ka increments. Model parameters chosen to match empirical LGM ice extent.
●	Abramowski *et al.*, 2006[19]	Point-source	High Asia; ^{10}Be dating
●	Arzhannikhov *et al.*, 2015[20]	Point-source	NE Asia; ^{10}Be dating
●	Baumann *et al.*, 1995[21]	Glacial curve	Western EIS; IRD. 5 data points shown.
●	Blomdin *et al.*, 2016[77]	Point-source	High Asia; ^{10}Be dating
●	Chevalier *et al.*, 2011[22]	Point-source	High Asia; ^{10}Be dating
●	Fu *et al.*, 2013[78]	Point-source	High Asia; ^{10}Be dating
●	Grin *et al.*, 2016[68]	Point-source	High Asia; overview of regional glaciations
●	Lekens *et al.*, 2009[26]	Glacial curve	Western EIS; based on seismic data and IRD
●	Li *et al.*, 2014[27]	Point-source	High Asia; ^{10}Be dating
●	Owen and Dortch, 2014[28]	Point-source	High Asia; TCN, OSL and ^{14}C dating. 2 data points shown.
●	Owen *et al.*, 2010[29]	Point-source	High Asia; ^{10}Be dating
●	Stübner *et al.*, 2017[31]	Point-source	High Asia; ^{10}Be dating
●	Thackray, 2008[32]	Point-source	LIS; ^{14}C and ^{36}Cl dating
●	Zhao *et al.*, 2013[46]	Point-source	High Asia; ERS and OSL dating
●	Zhao *et al.*, 2015[80]	Point-source	High Asia; ERS dating

Supplementary Table 6. Published evidence for the spatial extent of NH glaciation during MIS 5a. Key corresponds with colours in Supplementary Figure 4c.

MIS 5b (86–92 ka)

Key	Reference	Data type	Details
	Astakhov, 2004[81]	Empirical outline	Eastern EIS; Early Weichselian limit
	Astakhov, 2018[52]	Empirical outline	Eastern EIS; 70–90 ka
	Astakhov *et al.*, 2016[53]	Empirical outline	Eastern EIS; 80–90 ka
	Helmens, 2014[47]	Empirical outline	Western EIS
	Hjort, 1981[56]	Empirical outline	Tentative glacial limits in northern East GIS
	Kleman *et al.*, 2010[5]	Empirical outline	LIS; MIS 5b or d
	Mangerud *et al.*, 2011[34]	Empirical outline	EIS; 90 ka
	Olsen *et al.*, 2013[8]	Empirical outline	Western EIS; 90 ka
	Svendsen *et al.*, 2004[62]	Empirical outline	EIS; 90 ka
	Bonelli *et al.*, 2009[9]	Model	NH; spans 0–126 ka with 1 ka increments. Model driven by variations in orbital parameters and CO_2 concentration.
	de Boer *et al.*, 2014[10]	Model	NH; spans 0–410 ka with 1 ka increments. Ice volume and temperature derived from benthic $\delta^{18}O$ stack.
	Ganopolski and Calov, 2011[11]	Model	NH; spans 0–800 ka with 1 ka increments. Model driven by variations in orbital parameters and CO_2 concentration.
	Kleman *et al.*, 2002[65]	Model	LIS and CIS; 90 ka. Model driven by GRIP $\delta^{18}O$ ice core record and tuned to fit existing empirical data.
	Kleman *et al.*, 2013[58]	Model	NH; 86.2 ka. Model constrained by existing empirical data.
	Lambeck *et al.*, 2006[76]	Model	Western EIS; 94 ka. Model constructed using existing empirical data.
	Stokes *et al.*, 2012[17]	Model	CIS and LIS; 90 ka. Model calibrated against existing empirical data.
	Zweck and Huybrechts, 2005[18]	Model	NH; 30–120 ka with 10 ka increments. Model parameters chosen to match empirical LGM ice extent.
	Arzhannikhov *et al.*, 2015[20]	Point-source	NE Asia; ^{10}Be dating
	Baumann *et al.*, 1995[21]	Glacial curve	Western EIS; IRD. 5 data points shown.
	Eccleshall *et al.*, 2016[67]	Glacial curve	Western EIS; glacial curve based on OSL dating
	Fu *et al.*, 2013[78]	Point-source	High Asia; ^{10}Be dating
	Funder *et al.*, 1998[82]	Point-source	Eastern GIS; sedimentology, IRD and luminescence dating
	Grin *et al.*, 2016[68]	Point-source	High Asia; overview of regional glaciations
	Lekens *et al.*, 2009[26]	Glacial curve	Western EIS; based on seismic data and IRD
	Owen and Dortch, 2014[28]	Point-source	High Asia; TCN, OSL and ^{14}C dating. 2 data points shown.
	Owen *et al.*, 2010[29]	Point-source	High Asia; ^{10}Be dating
	Stauch and Lehmkuhl, 2010[50]	Point-source	NE Asia; IRSL dating
	Thackray, 2008[32]	Point-source	LIS; ^{14}C and ^{36}Cl dating

Key	Reference	Data type	Details
●	Zhao *et al.*, 2015[80]	Point-source	High Asia; ERS dating

Supplementary Table 7. Published evidence for the spatial extent of NH glaciation during MIS 5b. Key corresponds with colours in Supplementary Figure 5a.

MIS 5c (92–108 ka)

Key	Reference	Data type	Details
	Larsen *et al.*, 2006[48]	Empirical outline	EIS; 90–100 ka
	Lundqvist, 2004[59]	Empirical outline	EIS; 100 ka
	Mangerud *et al.*, 2011[34]	Empirical outline	EIS; 100 ka
	Möller *et al.*, 2015[60]	Empirical outline	Eastern EIS; MIS 5c–d
	Olsen *et al.*, 2013[8]	Empirical outline	Western EIS; 100 ka
	Bonelli *et al.*, 2009[9]	Model	NH; spans 0–126 ka with 1 ka increments. Model driven by variations in orbital parameters and CO_2 concentration.
	de Boer *et al.*, 2014[10]	Model	NH; spans 0–410 ka with 1 ka increments. Ice volume and temperature derived from benthic $\delta^{18}O$ stack.
	Ganopolski and Calov, 2011[11]	Model	NH; spans 0–800 ka with 1 ka increments. Model driven by variations in orbital parameters and CO_2 concentration.
	Lambeck *et al.*, 2006[76]	Model	EIS; 106 ka. Model constructed using existing empirical data.
	Stokes *et al.*, 2012[17]	Model	CIS and LIS; 100 ka. Model calibrated against existing empirical data.
	Zweck and Huybrechts, 2005[18]	Model	NH; 30–120 ka with 10 ka increments. Model parameters chosen to match empirical LGM ice extent.
●	Abramowski *et al.*, 2006[19]	Point-source	High Asia; ^{10}Be dating
●	Arzhannikhov *et al.*, 2015[20]	Point-source	NE Asia; ^{10}Be dating
●	Baumann *et al.*, 1995[21]	Glacial curve	Western EIS; IRD. 5 data points shown.
●	Fu *et al.*, 2013[78]	Point-source	High Asia; ^{10}Be dating
●	Grin *et al.*, 2016[68]	Point-source	High Asia; overview of regional glaciations
●	Lehmkuhl, 1998[25]	Point-source	High Asia; TL dating
●	Owen and Dortch, 2014[28]	Point-source	High Asia; TCN, OSL and ^{14}C dating
●	Owen *et al.*, 2010[29]	Point-source	High Asia; ^{10}Be dating
●	Thackray, 2008[32]	Point-source	LIS; ^{14}C and ^{36}Cl dating

Supplementary Table 8. Published evidence for the spatial extent of NH glaciation during MIS 5c. Key corresponds with colours in Supplementary Figure 5c.

MIS 5d (108–117 ka)

Key	Reference	Data type	Details
	Helmens, 2014[47]	Empirical outline	EIS
	Kleman *et al.*, 2010[5]	Empirical outline	LIS; MIS 5b or 5d
	Lundqvist, 2004[59]	Empirical outline	EIS; 110 ka
	Mangerud *et al.*, 2011[34]	Empirical outline	EIS; 110 ka
	Möller *et al.*, 2015[60]	Empirical outline	Eastern EIS; MIS 5c–d
	Olsen *et al.*, 2013[8]	Empirical outline	Western EIS; 110 ka
	Bonelli *et al.*, 2009[9]	Model	NH; spans 0–126 ka with 1 ka increments. Model driven by variations in orbital parameters and CO_2 concentration.
	de Boer *et al.*, 2014[10]	Model	NH; spans 0–410 ka with 1 ka increments. Ice volume and temperature derived from benthic δ^{18}O stack.
	Ganopolski and Calov, 2011[11]	Model	NH; spans 0–800 ka with 1 ka increments. Model driven by variations in orbital parameters and CO_2 concentration.
	Lambeck *et al.*, 2006[76]	Model	EIS; 106 ka. Model constructed using existing empirical data.
	Marshall *et al.*, 2000[44]	Model	LIS and CIS; 110 ka. Model driven by GRIP δ^{18}O ice core record and general circulation model.
	Stokes *et al.*, 2012[17]	Model	CIS and LIS; 110 ka. Model calibrated against existing empirical data.
	Zweck and Huybrechts, 2005[18]	Model	NH; 30–120 ka with 10 ka increments. Model parameters chosen to match empirical LGM ice extent.
	Arzhannikhov *et al.*, 2015[20]	Point-source	NE Asia; ^{10}Be dating
	Baumann *et al.*, 1995[21]	Glacial curve	Western EIS; IRD. 5 data points shown.
	Chadwick *et al.*, 1997[83]	Point-source	US mountains; ^{14}C and ^{36}Cl dating
	Eccleshall *et al.*, 2016[67]	Glacial curve	Western EIS; based on OSL dating
	Fu *et al.*, 2013[78]	Point-source	High Asia; ^{10}Be dating
	Funder, 1989[84]	Point-source	Northwest GIS; sedimentology, luminescence and ^{14}C dating.
	Funder *et al.*, 1998[82]	Point-source	Eastern GIS; glaciation at around 114 ka, from sedimentology, IRD and luminescence dating.
	Grin *et al.*, 2016[68]	Point-source	High Asia; overview of regional glaciations
	Karabanov *et al.*, 1998[85]	Point-source	Russia, TL dating
	Lekens *et al.*, 2009[26]	Glacial curve	Western EIS; based on seismic data and IRD
	Owen and Dortch, 2014[28]	Point-source	High Asia; TCN, OSL and ^{14}C dating. 2 data points shown.
	Owen *et al.*, 2010[29]	Point-source	High Asia; ^{10}Be dating
	Phillips *et al.*, 1997[86]	Point-source	US mountains; ^{14}C and ^{36}Cl dating
	Stauch and Lehmkuhl, 2010[50]	Point-source	NE Asia; IRSL dating
	Stein *et al.*, 1996[30]	IRD	East GIS; IRD, δ^{18}O and ^{14}C dating

Key	Reference	Data type	Details
●	Stübner *et al.*, 2017[31]	Point-source	High Asia; ^{10}Be dating
●	Thackray, 2008[32]	Point-source	LIS; ^{14}C and ^{36}Cl dating
●	Zech *et al.*, 2011[74]	Point-source	NE Asia; IRSL dating

Supplementary Table 9. Published evidence for the spatial extent of NH glaciation during MIS 5d. Key corresponds with colours in Supplementary Figure 6a.

MIS 6 (132–190 ka)

Key	Reference	Data type	Details
	Astakhov *et al.*, 2016[53]	Empirical outline	Eastern EIS
	Balco and Rovey, 2010[87]	Empirical outline	LIS; TCN dating suggests 3 ice advances between 0.2 and 0.75 Ma
	Barendregt *et al.*, 2014[88]	Empirical outline	LIS and CIS; constrained by palaeomagnetic data
	Barr and Solomina, 2015[41]	Empirical outline	NE Asia
	Basilian *et al.*, 2008[89]	Empirical outline	Arctic Ocean
	Böse *et al.*, 2012[90]	Empirical outline	Western EIS
	Curry *et al.*, 2011[91]	Empirical outline	LIS
	Ehlers *et al.*, 1990[92]	Empirical outline	EIS
	Ehlers *et al.*, 2011[93]	Empirical outline	EIS
	Eissmann, 2002[94]	Empirical outline	EIS
	Gibbard and Clark, 2011[95]	Empirical outline	Western EIS
	Gozhik *et al.*, 2010[96]	Empirical outline	EIS
	Hamblin *et al.*, 2005[97]	Empirical outline	Western EIS
	Hughes and Gibbard, 2018[98]	Empirical outline	EIS
	Jackson *et al.*, 2011[99]	Empirical outline	LIS
	Jakobsson *et al.*, 2008[100]	Empirical outline	Arctic Ocean
	Laban and van der Meer, 2011[101]	Empirical outline	Western EIS
	Marks, 2005[102]	Empirical outline	EIS; Saalian 1 (Odranian)
	Marks, 2011[103]	Empirical outline	Eastern EIS
	Marks *et al.*, 2018[104]	Empirical outline	EIS
	Möller *et al.*, 2015[60]	Empirical outline	Eastern EIS; Urdachsk and Sampesa moraines
	Niessen *et al.*, 2013[105]	Empirical outline	Arctic Ocean
	Roskosch *et al.*, 2015[106]	Empirical outline	EIS
	Svendsen *et al.*, 2004[62]	Empirical outline	EIS
	Turner *et al.*, 2016[64]	Empirical outline	CIS; MIS 4 and/or 6
	Colleoni *et al.*, 2016[107]	Model	NE Asia. Ice-sheet model forced by coupled atmosphere-ocean-sea-ice-land model.
	de Boer *et al.*, 2014[10]	Model	NH; spans 0–410 ka with 1 ka increments. Ice volume and temperature derived from benthic $\delta^{18}O$ stack.
	Ganopolski and Calov, 2011[11]	Model	NH; spans 0–800 ka with 1 ka increments. Model driven by variations in orbital parameters and CO_2 concentration.
	Lambeck *et al.*, 2006[76]	Model	EIS; 106 ka. Model constructed using existing empirical data.
	Peltier, 2004[108]	Model	LIS; Colleoni *et al.* (2016)[106] use the 13 ka LIS of Peltier (2004)[107] to show a small LIS during MIS 6.
	Anderson *et al.*, 2012[109]	Point-source	US Mountains; geomorphological mapping

Key	Reference	Data type	Details
●	Barendregt *et al*., 2014[88]	Point-source	LIS and CIS; spans 0.13–0.78 Ma. 37 data points shown.
●	Baumann *et al*., 1995[21]	Glacial curve	Western EIS; IRD. 5 data points shown.
●	Chadwick *et al*., 1997[83]	Point-source	US mountains; ^{14}C and ^{36}Cl dating
●	Chevalier *et al*., 2011[22]	Point-source	High Asia; ^{10}Be dating
●	Dahlgren *et al*., 2002[110]	Glacial curve	Western EIS; based on seismic data
●	Eccleshall *et al*., 2016[67]	Glacial curve	Western EIS; based on OSL dating
●	Eissmann, 2002[94]	Point-source	Western EIS; stratigraphy
●	Fu *et al*., 2013[78]	Point-source	High Asia; ^{10}Be dating
●	Funder, 1989[84]	Point-source	Northwest GIS; sedimentology, luminescence and ^{14}C dating.
●	Funder *et al*., 1998[82]	Point-source	Eastern GIS; sedimentology, IRD and luminescence dating.
●	Geirsdóttir *et al*., 2007[111]	Point-source	Iceland; sedimentology and K–Ar dating
●	Hall and Shroba, 1995[69]	Point-source	US mountains; soil properties
●	Hibbert *et al*., 2010[24]	IRD	Western EIS
●	Hjelstuen *et al*., 2005[112]	Seismic data	Western EIS; seismic stratigraphy
●	Kuhle, 2007[113]	Point-source	High Asia; geomorphological mapping
●	Lehmkuhl, 1998[25]	Point-source	High Asia; TL dating
●	Lekens *et al*., 2009[26]	Glacial curve	Western EIS; based on seismic data and IRD
●	Li *et al*., 2014[27]	Point-source	High Asia; ^{10}Be dating
●	Licciardi and Pierce, 2008[114]	Point-source	US mountains; ^{10}Be dating
●	Montelli *et al*., 2017[115]	Seismic data	Western EIS; seismic stratigraphy
●	Nielsen and Kuijpers, 2013[116]	Seismic data	Southwest GIS; seismic stratigraphy
●	Nikolskiy *et al*., 2017[117]	Point-source	NE Asia; <190–210 ka
●	O’Regan *et al*., 2017[118]	Seismic data	NE Asia; seismic stratigraphy
●	Owen and Dortch, 2014[28]	Point-source	High Asia; TCN, OSL and ^{14}C dating. 2 data points shown.
●	Owen *et al*., 2006[49]	Point-source	High Asia; TCN dating
●	Owen *et al*., 2010[29]	Point-source	High Asia; ^{10}Be dating
●	Phillips *et al*., 1997[86]	Point-source	US Mountains; ^{10}Be and ^{36}Cl dating
●	Sejrup *et al*., 2000[70]	Glacial curve	Western EIS; from seismic data
●	Sejrup *et al*., 2005[71]	Glacial curve	Western EIS; from seismic data
●	Stauch and Lehmkuhl, 2010[50]	Point-source	NE Asia; IRSL dating
●	Stein *et al*., 1996[30]	IRD	East GIS; IRD, δ^{18}O and ^{14}C dating.
●	Stewart and Lonergan, 2011[72]	Seismic data	Western EIS; seismic stratigraphy
●	Strunk *et al*., 2017[119]	Glacial curve	West GIS; modelling and ^{10}Be–^{26}Al dating. 4 data points shown.
●	Stübner *et al*., 2017[31]	Point-source	High Asia; ^{10}Be dating
●	Vorren and Laberg, 1997[120]	Seismic data	EIS
●	Zech *et al*., 2011[74]	Point-source	NE Asia; Sedimentology and IRSL dating

Key	Reference	Data type	Details
●	Zhao *et al*., 2009[45]	Point-source	High Asia; ESR dating
●	Zhao *et al*., 2013[46]	Point-source	High Asia; ESR and OSL dating
●	Zhao *et al*., 2015[80]	Point-source	High Asia; ESR dating

Supplementary Table 10. Published evidence for the spatial extent of NH glaciation during MIS 6. Key corresponds with colours in Supplementary Figure 6c.

MIS 8 (243–279 ka)

Key	Reference	Data type	Details
	Astakhov *et al.*, 2016[53]	Empirical outline	Eastern EIS; Samarovo limit
	Balco and Rovey, 2010[87]	Empirical outline	LIS; TCN dating suggests 3 ice advances between 0.2 and 0.75 Ma
	Hughes and Gibbard, 2018[98]	Empirical outline	EIS
	Marks, 2011[103]	Empirical outline	EIS; Krznanian limit
	White *et al.*, 2010[121]	Empirical outline	Western EIS
	White *et al.*, 2017[122]	Empirical outline	Western EIS
	de Boer *et al.*, 2014[10]	Model	NH; spans 0–410 ka with 1 ka increments. Ice volume and temperature derived from benthic δ^{18}O stack.
	Ganopolski and Calov, 2011[11]	Model	NH; spans 0–800 ka with 1 ka increments. Model driven by variations in orbital parameters and CO_2 concentration.
	Beets *et al.*, 2005[123]	Point-source	Western EIS; seismic profiles and AAR dating
	Chevalier *et al.*, 2011[22]	Point-source	High Asia; ^{10}Be dating
	Dahlgren *et al.*, 2002[110]	Glacial curve	Western EIS; based on seismic data
	Geirsdóttir *et al.*, 2007[111]	Point-source	Iceland; sedimentology and K–Ar dating
	Hjelstuen *et al.*, 2005[112]	Seismic data	Western EIS; seismic stratigraphy
	Hodell *et al.*, 2008[124]	IRD	LIS; age model from IRD and ^{14}C dating
	Krissek, 1995[125]	IRD	CIS and NE Asia; marine-calving margin at 0.27–0.29 Ma
	Montelli *et al.*, 2017[115]	Seismic data	Western EIS; seismic stratigraphy
	Owen and Dortch, 2014[28]	Point-source	High Asia; TCN, OSL and ^{14}C dating
	Phillips *et al.*, 1997[86]	Point-source	US Mountains; ^{10}Be and ^{36}Cl dating
	Roskosch *et al.*, 2015[106]	Point-source	Western EIS; OSL dating
	Sejrup *et al.*, 2000[70]	Glacial curve	Western EIS; from seismic data
	Sejrup *et al.*, 2005[71]	Glacial curve	Western EIS; from seismic profiles
	Stewart and Lonergan, 2011[72]	Seismic data	Western EIS; seismic stratigraphy
	Strunk *et al.*, 2017[119]	Glacial curve	West GIS; modelling and ^{10}Be–^{26}Al dating. 4 data points shown.
	Vorren and Laberg, 1997[120]	Seismic data	EIS

Supplementary Table 11. Published evidence for the spatial extent of NH glaciation during MIS 8. Key corresponds with colours in Supplementary Figure 7a.

MIS 10 (337–365 ka)

Key	Reference	Data type	Details
	Balco and Rovey, 2010[87]	Empirical outline	LIS; TCN dating suggests 3 ice advances between 0.2 and 0.75 Ma
	Böse *et al.*, 2012[90]	Empirical outline	Western EIS
	Hamblin *et al.*, 2005[97]	Empirical outline	Western EIS
	Marks, 2011[103]	Empirical outline	EIS; Krznanian limit
	Roskosch *et al.*, 2015[106]	Empirical outline	Western EIS
	de Boer *et al.*, 2014[10]	Model	NH; spans 0–410 ka with 1 ka increments. Ice volume and temperature derived from benthic δ^{18}O stack.
	Ganopolski and Calov, 2011[11]	Model	NH; spans 0–800 ka with 1 ka increments. Model driven by variations in orbital parameters and CO_2 concentration.
●	Dahlgren *et al.*, 2002[110]	Glacial curve	Western EIS; based on seismic data
●	Eissmann, 2002[94]	Point-source	Western EIS; stratigraphic sections
●	Geirsdóttir *et al.*, 2007[111]	Point-source	Iceland; sedimentology and K–Ar dating
●	Hjelstuen *et al.*, 2005[112]	Seismic data	Western EIS; seismic stratigraphy
●	Hodell *et al.*, 2008[124]	IRD	LIS; age model from IRD and ^{14}C dating
●	Montelli *et al.*, 2017[115]	Seismic data	Western EIS; seismic stratigraphy
●	Owen and Dortch, 2014[28]	Point-source	High Asia; TCN, OSL and ^{14}C dating. 2 data points shown.
●	Owen *et al.*, 2009[38]	Point-source	High Asia; TCN and OSL dating
●	Owen *et al.*, 2010[29]	Point-source	High Asia; TCN dating
●	Phillips *et al.*, 1997[86]	Point-source	US Mountains; ^{10}Be and ^{36}Cl dating
●	Sejrup *et al.*, 2000[70]	Glacial curve	Western EIS; from seismic data
●	Sejrup *et al.*, 2005[71]	Glacial curve	Western EIS; from seismic data
●	Spooner *et al.*, 1996[126]	Point-source	CIS; stratigraphy and palaeomagnetic data
●	Stewart and Lonergan, 2011[72]	Seismic data	Western EIS; seismic stratigraphy
●	Strunk *et al.*, 2017[119]	Glacial curve	West GIS; modelling and ^{10}Be–^{26}Al dating. 4 data points shown.
●	Vorren and Laberg, 1997[120]	Seismic data	EIS

Supplementary Table 12. Published evidence for the spatial extent of NH glaciation during MIS 10. Key corresponds with colours in Supplementary Figure 7c.

MIS 12 (429–477 ka)

Key	Reference	Data type	Details
	Astakhov *et al.*, 2016[53]	Empirical outline	Eastern EIS; Lebed glaciation
	Balco and Rovey, 2010[87]	Empirical outline	LIS; TCN dating suggests 3 ice advances between 0.2 and 0.75 Ma
	Böse *et al.*, 2012[90]	Empirical outline	Western EIS
	Ehlers *et al.*, 1990[92]	Empirical outline	EIS; older Saalian
	Ehlers *et al.*, 2011[93]	Empirical outline	EIS; Elsterian glaciation
	Eissmann, 2002[94]	Empirical outline	EIS; Don lobe is shown as MIS 12
	Gibbard and Clark, 2011[95]	Empirical outline	EIS
	Gozhik *et al.*, 2010[96]	Empirical outline	EIS
	Hamblin *et al.*, 2005[97]	Empirical outline	Western EIS
	Hughes and Gibbard, 2018[98]	Empirical outline	EIS
	Krzyszkowski *et al*, 2015[127]	Empirical outline	EIS; Elsterian T2 till
	Laban and van der Meer, 2011[101]	Empirical outline	EIS
	Marks, 2011[103]	Empirical outline	EIS; Sanian 2 limit
	Marks *et al.*, 2018[104]	Empirical outline	EIS; Elsterian, Sanian 2 and Berezinian limits
	Roskosch *et al.*, 2015[106]	Empirical outline	EIS
	Ganopolski and Calov, 2011[11]	Model	NH; spans 0–800 ka with 1 ka increments. Model driven by variations in orbital parameters and CO_2 concentration.
●	Dahlgren *et al.*, 2002[110]	Glacial curve	Western EIS; based on seismic data
●	Geirsdóttir *et al.*, 2007[111]	Point-source	Iceland; sedimentology and K–Ar dating
●	Hjelstuen *et al.*, 2005[112]	Seismic data	Western EIS; seismic stratigraphy
●	Hodell *et al.*, 2008[124]	IRD	LIS; age model from IRD and ^{14}C dating
●	Montelli *et al.*, 2017[115]	Seismic data	Western EIS; seismic stratigraphy
●	Owen *et al.*, 2006[49]	Point-source	High Asia; TCN dating
●	Phillips *et al.*, 1997[86]	Point-source	US Mountains; ^{10}Be and ^{36}Cl dating
●	Sejrup *et al.*, 2000[70]	Glacial curve	Western EIS; from seismic data
●	Sejrup *et al.*, 2005[71]	Glacial curve	Western EIS; glacial curves from seismic profiles
●	Stewart and Lonergan, 2011[72]	Seismic data	Western EIS; seismic stratigraphy
●	Strunk *et al.*, 2017[119]	Glacial curve	West GIS; modelling and ^{10}Be–^{26}Al dating. 4 data points shown.
●	Vorren and Laberg, 1997[120]	Seismic data	EIS
●	Zhao *et al.*, 2009[45]	Point-source	High Asia; ESR dating
●	Zhao *et al.*, 2015[80]	Point-source	High Asia; ERS dating

Supplementary Table 13. Published evidence for the spatial extent of NH glaciation during MIS 12. Key corresponds with colours in Supplementary Figure 8a.

MIS 16 (622–677 ka)

Key	Reference	Data type	Details
	Aber, 1991[128]	Empirical outline	LIS; Pre-Illinoian glaciation
	Astakhov, 2004[81]	Empirical outline	Eastern EIS; Donian glaciation
	Astakhov *et al.*, 2016[53]	Empirical outline	Eastern EIS; Donian glaciation
	Balco and Rovey, 2010[87]	Empirical outline	LIS; TCN dating suggests 3 ice advances between 0.75 and 0.2 Ma
	Colgan, 1999[129]	Empirical outline	LIS; Pre-Illinoian glaciation
	Gozhik *et al.*, 2010[96]	Empirical outline	EIS; Donian/ Sanian 1 glaciation
	Hamblin *et al.*, 2005[97]	Empirical outline	Western EIS; Happisburgh Formation
	Hughes and Gibbard, 2018[98]	Empirical outline	EIS; Donian glaciation
	Marks, 2011[103]	Empirical outline	EIS; Sanian 1 glaciation
	Marks *et al.*, 2018[104]	Empirical outline	EIS; Donian/ Sanian 1 glaciation
	Olsen *et al.*, 2013[8]	Empirical outline	EIS; transitional phase at 0.5–1.5 Ma
	Toucanne *et al.*, 2009[130]	Empirical outline	EIS; pre-MIS 12 glaciations, based on IRD
●	Chadwick *et al.*, 1997[83]	Point-source	LIS; ^{10}Be and ^{36}Cl dating
●	Colgan, 1999[129]	Point-source	LIS; sedimentology and palaeomagnetism
●	Geirsdóttir *et al.*, 2007[111]	Point-source	Iceland; sedimentology and K–Ar dating
●	Hodell *et al.*, 2008[124]	IRD	LIS; age model from IRD and ^{14}C dating
●	Montelli *et al.*, 2017[115]	Seismic data	Western EIS; seismic stratigraphy
●	Phillips *et al.*, 1997[86]	Point-source	US Mountains; ^{10}Be and ^{36}Cl dating
●	Strunk *et al.*, 2017[119]	Glacial curve	West GIS; modelling and ^{10}Be–^{26}Al dating. 4 data points shown.
●	Vorren and Laberg, 1997[120]	Seismic data	EIS

Supplementary Table 14. Published evidence for the spatial extent of NH glaciation during MIS 16. Key corresponds with colours in Supplementary Figure 8c.

MIS 20–24 (790–928 ka)

Key	Reference	Data type	Details
	Andriashek and Barendregt, 2017[131]	Empirical outline	LIS; MIS 20, around 0.8 Ma. 40 data points shown.
	Balco and Rovey, 2010[87]	Empirical outline	LIS; TCN dating indicates ice advance around 0.8 Ma
	Batchelor *et al.*, 2017[132]	Empirical outline	Western EIS: hypothesised ice sheet *c.*1 Ma
	Gozhik *et al.*, 2010[96]	Empirical outline	EIS; Nidanian glaciation is MIS 20 or 22
	Marks, 2011[103]	Empirical outline	EIS; Nidanian glaciation, around 0.9 Ma
	Olsen *et al.*, 2013[8]	Empirical outline	EIS; transitional phase at 0.5–1.5 Ma
	Ottesen *et al.*, 2018[133]	Empirical outline	Western EIS; ice sheet *c.*1 Ma
	Toucanne *et al.*, 2009[130]	Empirical outline	EIS; pre-MIS 12 glaciations, based on IRD
●	Anderson *et al.*, 2012[109]	Point-source	US mountains; mapped glacial deposits
●	Andriashek and Barendregt, 2017[131]	Point-source	LIS; palaeomagnetic dating
●	Bierman *et al.*, 2016[134]	IRD	Southeast GIS; IRD peak at 0.8 Ma. 2 data points shown.
●	Geirsdóttir *et al.*, 2007[111]	Point-source	Iceland; sedimentology and K–Ar dating
●	Krissek, 1995[125]	IRD	CIS and NE Asia; marine-calving margin at 0.92–0.93 Ma. 3 data points shown.
●	Laberg *et al.*, 2013[135]	Seismic data	East GIS; multiple shelf-break glaciations between 0.8 and 1.8 Ma
●	Montelli *et al.*, 2017[115]	Seismic data	Western EIS; seismic stratigraphy
●	Sejrup *et al.*, 1991[136]	Point-source	Western EIS; palaeomagnetic dating suggests grounded ice sheet at around 0.85 Ma
●	Sejrup *et al.*, 2000[70]	Glacial curve	Western EIS; from seismic data
●	Strunk *et al.*, 2017[119]	Glacial curve	West GIS; modelling and ^{10}Be–^{26}Al dating. 4 data points shown.
●	Thierens *et al.*, 2012[137]	IRD	West EIS; 0.65–1.2 Ma

Supplementary Table 15. Published evidence for the spatial extent of NH glaciation during MIS 20–24. Key corresponds with colours in Supplementary Figure 9a.

Early Matuyama palaeomagnetic Chron (1.78–2.6 Ma)

Key	Reference	Data type	Details
	Balco and Rovey, 2010[87]	Empirical outline	LIS; TCN dating indicates ice advance around 2.4 Ma
	Barendregt and Duk-Rodkin, 2011[138]	Empirical outline	LIS and CIS; 1.78–2.6 Ma, palaeomagnetic dating
	Barendregt *et al.*, 2014[88]	Empirical outline	LIS and CIS; 1.78–2.6 Ma, palaeomagnetic dating
	Batchelor *et al.*, 2017[132]	Empirical outline	Western EIS: ice sheet at onset of Quaternary
	Dowdeswell and Ottesen, 2013[139]	Seismic data	Western EIS
	Kleman *et al.*, 2008[140]	Empirical outline	EIS; 1.0–2.6 Ma
	Knies *et al.*, 2009[141]	Empirical outline	EIS; maximum and minimum versions based on compilation of empirical data
	Olsen *et al.*, 2013[8]	Empirical outline	EIS; onshore phase at 1.5–2.5 Ma
	Ottesen *et al.*, 2018[133]	Empirical outline	Western EIS; ice sheet *c.*1.6 Ma
	Rea *et al.*, 2018[142]	Empirical outline	EIS; seismic data, from 2.53 Ma
	Solgaard *et al.*, 2011[143]	Model	GIS: 3 models for ice expansion at 2.4–3 Ma. Ice flow model constrained by geological observations and climate reconstructions.
●	Bailey *et al.*, 2013[144]	IRD	IRD peak at 2.52 Ma traced to Archaean basement rocks of GIS
●	Barendregt *et al.*, 2014[88]	Point-source	LIS; palaeomagnetic dating. 20 data points shown.
●	Berger and Jokat, 2009[145]	Seismic data	Northeast GIS; onset of margin progradation around 2.5 Ma
●	Bierman *et al.*, 2016[134]	IRD	Southeast GIS; IRD peak at 1.9 Ma. 2 data points shown.
●	Butt *et al.*, 2001[146]	Seismic data	East GIS; seismic data and palaeomagnetic dating
●	Geirsdóttir *et al.*, 2007[111]	Point-source	Iceland; sedimentology and K–Ar dating
●	Hidy *et al.*, 2013[147]	Point-source	CIS; TCN dating
●	Hofmann *et al.*, 2016[148]	Seismic data	West GIS; seismic stratigraphy
●	Jansen *et al.*, 2000[149]	IRD	West EIS; IRD peaks at 2.1 and 2.4 Ma
●	Krissek, 1995[125]	IRD	CIS and NE Asia; marine-calving margin at 2.6 Ma. 3 data points shown.
●	Laberg *et al.*, 2013[135]	Seismic data	East GIS; seismic data and palaeomagnetic dating
●	Montelli *et al.*, 2017[115]	Seismic data	Western EIS; seismic stratigraphy
●	Nielsen and Kuijpers, 2013[116]	Seismic data	Southwest GIS; age of 2.5 Ma suggested from seismic stratigraphy
●	Thierens *et al.*, 2012[137]	IRD	West EIS; marine-calving margin at 2.5 Ma

Supplementary Table 16. Published evidence for the spatial extent of NH glaciation during the early Matuyama Chron. Key corresponds with colours in Supplementary Figure 9c.

Late Gauss palaeomagnetic Chron (2.6–3.59 Ma)

Key	Reference	Data type	Details
	Barendregt and Duk-Rodkin, 2011[138]	Empirical outline	CIS; 1.78–2.6 Ma, palaeomagnetic dating
	Barendregt *et al.*, 2014[88]	Empirical outline	CIS; 2.6–3.6 Ma
	Batchelor *et al.*, 2017[132]	Empirical outline	Western EIS: ice sheet at onset of Quaternary
	Knies *et al.*, 2009[141]	Empirical outline	EIS; maximum and minimum versions based on compilation of empirical data
	Ottesen *et al.*, 2018[133]	Empirical outline	Western EIS; ice sheet at onset of Quaternary
	Solgaard *et al.*, 2011[143]	Model	GIS: 3 models for ice expansion at 2.4–3 Ma. Ice flow model constrained by geological observations and climate reconstructions.
	Bailey *et al.*, 2013[144]	IRD	IRD peak at 2.64 Ma traced to Archaean basement rocks of GIS
	Barendregt *et al.*, 2014[88]	Point-source	CIS; palaeomagnetic dating. 9 data points shown.
	Bierman *et al.*, 2016[134]	IRD	Southeast GIS; IRD peak at 2.8 Ma
	Butt *et al.*, 2001[146]	Seismic data	East GIS; seismic data and palaeomagnetic dating
	Geirsdóttir *et al.*, 2007[111]	Point-source	Iceland; sedimentology and K–Ar dating
	Hidy *et al.*, 2013[147]	Point-source	CIS; TCN dating
	Hofmann *et al.*, 2016[148]	Seismic data	West GIS; seismic stratigraphy
	Jansen *et al.*, 2000[149]	IRD	West EIS; IRD peaks at 3.3 and 2.74 Ma
	Krissek, 1995[125]	IRD	CIS and NE Asia; marine-calving margin at 2.6 Ma. 3 data points shown.
	Thierens *et al.*, 2012[137]	IRD	West EIS; marine-calving margin at 2.6 Ma

Supplementary Table 17. Published evidence for the spatial extent of NH glaciation during the late Gauss Chron. Key corresponds with colours in Supplementary Figure 10a.

Supplementary Notes

Supplementary Note 1: The Last Glacial Maximum (LGM)

The Last Glacial Maximum (LGM) best-estimate reconstruction is based on the LGM extent of Ehlers *et al.*[93], which was derived from a compilation of published empirical datasets. In this reconstruction, the Greenland Ice Sheet (GIS) is shown at the shelf break, following marine geophysical work that has identified subglacially formed landforms on the outermost shelf[151-153]. Grounded ice is also extended to the shelf break on Grand Banks and beyond Baffin Island, British Columbia and western Britain[4,154]. A lobe of the Cordilleran Ice Sheet (CIS) is shown to enter the Puget Lowlands during this time[155]. The LGM outline of Barr and Clark[156], which is more detailed than that of Ehlers *et al.*[93], is used in north-east (NE) Asia.

Robustness score

Mean robustness score: 5 (ice-sheet-wide empirical outlines)

Supplementary Note 2: 30 ka

The reader should refer to Supplementary Figure 2a for a map of previously published data on ice-sheet extent at 30 ka, Supplementary Figure 2b for a map of the maximum, minimum and best-estimate ice-sheet reconstructions, and Supplementary Table 1 for details of the data sources used to inform these reconstructions.

Maximum estimate of the 30 ka ice-sheet extent

The maximum ice extent in Europe at 30 ka is based mainly on empirical data and the ice-sheet extent at the LGM. The empirical outlines of Larsen *et al.*[6], Hughes *et al.*[4] and Marks[7] are followed over western Europe and Scandinavia. The EIS is extended to the east to include Finland because of the geometry of the ice in northern Poland. Hughes *et al.*[4] keep Finland ice-free in their reconstruction for 30–32 ka, but note that they find it likely that ice expansion to the south was matched by ice growth to the east. Over northern Siberia, the LGM extent is used for the Barents-Kara Sea, and the maximum MIS 4 extent is used for the Putorana Plateau in central Siberia. Ice is shown to extend to the shelf break beyond northern Britain and Norway, as suggested by ice-rafted debris (IRD) records[21,25]. Ice is shown in the North Sea, and ice in Greenland and Iceland is shown at the shelf break. Ice is also shown at the shelf break along the northern, northwestern and eastern margin of the Laurentide Ice Sheet (LIS). The southern and western margin of the LIS is the larger of the two empirically derived outlines of Dyke *et al.*[2] and Kleman *et al.*[5]. The CIS is shown at its LGM extent[93]. The maximum Quaternary ice-sheet extent template is used in NE Asia, which is a combination of the maximum Quaternary limits of Glushkova[55] and Barr and Clark[156] (see Methods).

Minimum estimate of the 30 ka ice-sheet extent

The minimum ice extent in Europe at 30 ka is based on the outline of Hughes *et al.*[4] for 30–32 ka, which is a compilation of empirical evidence. Ice in Greenland and Iceland is shown at the present-day coastline. The LIS is the smaller of the two empirically derived outlines of Dyke *et al.*[2] and Kleman *et al.*[5]. The minimum ice extent was further reduced in west-central Canada by ~500 km to account for the possibility of an ice-free interval in that area as indicated by thermoluminescence dating of non-glacial sediments[157]. The CIS extent is based on the 30 ka regional model of Seguinot *et al.*[16]. The LGM extent of Barr and Clark[156] is used in NE Asia.

Best-estimate of the 30 ka ice-sheet extent

The minimum ice extent in Europe at 30 ka, which is the empirically derived reconstruction of Hughes *et al*.[4], is generally used as the best-estimate for the 30 ka ice-sheet reconstruction. The exception is that the ice sheet is extended to the shelf break in the northern North Sea to account for the probable operation of the Norwegian Channel Ice Stream during this time[26,70,158]. Our best-estimate reconstruction does not include the tentative outlines of Marks[7] in Poland and Lithuania, which span 33–37 ka. Ice in Greenland is shown in a mid-shelf position, following the suggestion that the ice sheet was on the continental shelf during this time[82]. Ice in Iceland is shown at the present-day coastline. The detailed empirically derived reconstruction of Dyke *et al*.[2] is followed for the best-estimate LIS at 30 ka. Although the 1-sigma errors on Berger and Nielsen's[157] geochronological data overlap with the 30 ka interval, it is more likely that this part of the Hudson Bay Lowlands was ice-free closer to the 40 ka interval, as supported by various radiocarbon dates[159]. To be conservative, the best-estimate for the CIS and ice in NE Asia at 30 ka is the same as the minimum.

Robustness scores for the 30 ka ice-sheet reconstruction

EIS 4 (empirical outlines constrain much of the ice margin)
LIS 5 (ice-sheet-wide empirical outlines)
CIS 1 (modelled outlines)
NE Asia 1 (modelled outlines)
Mean robustness score: 2.75

Supplementary Note 3: 35 ka

The reader should refer to Supplementary Figure 2c for a map of previously published data on ice-sheet extent at 35 ka, Supplementary Figure 2d for a map of the maximum, minimum and best-estimate ice-sheet reconstructions, and Supplementary Table 2 for details of the data sources used to inform these reconstructions.

Maximum estimate of the 35 ka ice-sheet extent

For the maximum European Ice Sheet (EIS) at 35 ka, the empirical outlines of Houmark-Nielsen[3], Obst *et al*.[35] and Marks[7] in northern Germany and Poland are merged with the outline of Olsen *et al*.[8] in Scandinavia and the LGM ice extent in the Barents-Kara Sea. The maximum MIS 4 ice extent is used for the Putorana Plateau in central Siberia. Ice in Greenland and Iceland is shown at the present-day shelf break. For the maximum LIS, ice is shown at the shelf break along the northern and eastern margin. The empirically derived late MIS 3 outline of Kleman *et al*.[5] is used to the south. The western LIS margin is the modelled outline of Ganopolski and Calov[11], which keeps the LIS and CIS separate during this time[160]. The LGM ice extent is used for the CIS, and the maximum Quaternary ice-extent template[55,156] is used for NE Asia (see Methods).

Minimum estimate of the 35 ka ice-sheet extent

For the minimum EIS at 35 ka, the larger of the two empirically derived outlines provided by Hughes *et al*.[4] for the period 34–38 ka is used. Ice in Greenland and Iceland is shown at the coastline. A schematic ice cap is shown over Scotland, which is based on the minimum modelled ice extent in Britain during MIS 4[18]. The empirically derived late MIS 3 outline of Kleman *et al*.[5] is used for the minimum LIS at 35 ka, but is not allowed to extend beyond the detailed empirical reconstruction of Dyke *et al*.[2] for 30 ka. The minimum ice extent was further reduced in west-central Canada by ~200 km to account for the possibility of an ice-free interval in the Hudson Bay Lowlands as indicated by thermoluminescence work on non-glacial sediments[157]. The minimum LIS extent was also reduced in the Ungava Peninsula, Canada, by ~200 km to account for the possibility of an ice-free interval as indicated by various radiocarbon ages on non-glacial sediments[161]. Because of an absence of data, only coastal mountain glaciers are shown for the CIS and no ice is shown in NE Asia.

<u>Best-estimate</u> of the 35 ka ice-sheet extent

To be conservative, the minimum ice-sheet extents over Britain and Iceland are used for the 35 ka best-estimate. The tentative outlines of Obst *et al.*[35], Marks[7] and Olsen *et al.*[8], which show the EIS extending into northern Germany, Poland and Finland during this time, are not included. Instead, the ice sheet is shown following the present-day coastline around Norway and Sweden, in agreement with the empirical reconstruction of Houmark-Nielsen[3] and IRD records off southern and western Norway[21,26]. Ice in Greenland is shown in a mid-shelf position, following Funder *et al.*[82], who suggest that the ice sheet was on the continental shelf during this time. The minimum LIS at 35 ka is used as the best-estimate in most areas. This outline is based on the empirically derived late MIS 3 outline of Kleman *et al.*[5] and the detailed empirical reconstruction of Dyke *et al.*[2] for 30 ka. Although the 1-sigma errors on Berger and Nielsen's[157] geochronological data overlap with the 35 ka interval, it is more likely that these sites in the Hudson Bay Lowlands were ice-free closer to the 40 ka interval, as supported by various radiocarbon dates[159]. The Ungava Peninsula in Canada is shown as ice-covered. Although there is no evidence to rule out the possibility that this region was ice-free at 35 ka, Guyard *et al.*[161] suggest that their radiocarbon ages may represent a minimum age estimate owing to the suspected mixing of older and younger carbon in the sample. To be conservative, the best-estimate 30 ka ice extent is used for the CIS[16], and the LGM of Barr and Clark[156] is used for NE Asia.

Robustness scores for the 35 ka ice-sheet reconstruction

EIS 3 (regional empirical outlines of contrasting extent)
LIS 3 (single broad-scale empirical outline)
CIS 1 (modelled outlines)
NE Asia 1 (modelled outlines)
Mean robustness score: 2

Supplementary Note 4: 40 ka

The reader should refer to Supplementary Figure 3a for a map of previously published data on ice-sheet extent at 40 ka, Supplementary Figure 3b for a map of the maximum, minimum and best-estimate ice-sheet reconstructions, and Supplementary Table 3 for details of the data sources used to inform these reconstructions.

Maximum estimate of the 40 ka ice-sheet extent

For the maximum EIS at 40 ka, the maximum modelled ice extent over Europe is used, because the outline of Arnold *et al*.[33] is a minimum estimate and the outline of Houmark-Nielsen[3] depicts ice at 34–46 ka. The modelled outlines selected are not allowed to be larger than at the LGM. This means that the LGM limit is used everywhere except for Britain and the North Sea[10], Poland[10, 18] and western Russia[18]. Ice in Greenland and Iceland is shown at the present-day shelf break. Ice is also shown at the shelf break for the northern and eastern margin of the LIS. For the southern LIS margin, the largest modelled outline is used[18] but is not allowed to be larger than at the LGM. The modelled outline of Ganopolski and Calov[11] is used for the western LIS margin because it keeps the LIS and CIS separate[160]. Because of an absence of empirical data, the LGM extent[93] is used for the CIS. For NE Asia, the maximum Quaternary ice-extent template (based on Glushkova[55] and Barr and Clark[156]), is used to account for the large ice-sheet outline of Barr and Solomina[41].

Minimum estimate of the 40 ka ice-sheet extent

For the minimum EIS at 40 ka, ice is not shown in Britain, as in Hughes *et al*.[4]. The outline of Houmark-Nielsen[3] is combined with the maximum outline of Hughes *et al*.[4] (34–38 ka) over Scandinavia. The present-day ice cover is used for the islands of the Barents and Kara seas, and ice is shown to the coastline for Greenland and Iceland. For the LIS, hypothesis 2 of Dredge and Thorleifson[42] is used, which shows small ice-dispersal areas. This minimum ice extent is supported by geochronological data from the Hudson Bay Lowlands, Canada, which show the development of peatlands and boreal forests in this region at ~40 ka[159]. Coastal mountain glaciers are shown for the CIS, and the LGM extent of Barr and Clark[156] is used for NE Asia.

Best-estimate of the 40 ka ice-sheet extent

We note that the 40 ka interval immediately preceded a time of rapid ice growth in the NH[162,163.]. For the best-estimate EIS at 40 ka, the empirical outlines of Houmark-Nielsen[3], Hughes *et al*.[4] and van Andel and Tzedakis[43] are used but are not allowed to extend beyond the 35 ka best-estimate along the eastern margin. The ice sheet is shown on the continental shelf off southern and western Norway, in agreement with IRD records[21,26] and suggestions that the southern Fennoscandian Ice Sheet (FIS) extended beyond the coastline around 42 ka[43,164,165]. To be conservative, the minimum ice extent is followed in the Barents and Kara seas and Iceland. As a mid-point between our minimum and maximum reconstructions, a schematic ice cap is placed over Scotland, which is based mainly on the minimum modelled ice extent in Britain during MIS 4[18]. Ice is extended onto the continental shelf to the north of Scotland, as suggested by Lekens *et al*.[26]. However, it is worth noting that Hughes *et al*.[4] do not include any ice over Britain in their reconstruction for 34–38 ka. Ice in Greenland is shown in a mid-shelf position, following Funder *et al*.[82] who suggest that the ice sheet was on the continental shelf during this time.

Over North America, the minimum outline, based on hypothesis 2 of Dredge and Thorleifson[42], is used for the LIS. This ice extent is supported by geochronological data from the Hudson Bay Lowlands, Canada, showing the development of peatlands and boreal forests in this region at ~40 ka[159]. Recently, the feasibility of such a reduced ice extent was demonstrated by reconciling geological data from the Hudson Bay Lowlands with estimates of sea level and isostatic adjustment for this area[166]. Deglaciation of Hudson Bay at ~40 ka is also supported by 8 radiocarbon dates on shells from Wager Bay[167]. The 30 ka ice-extent template (see Methods) is used for the CIS[16], and the Quaternary maximum ice-extent template is used in NE Asia[55,156].

Robustness scores for the 40 ka ice-sheet reconstruction

EIS 3 (ice-sheet-wide empirical outlines of contrasting extent)

LIS 3 (ice-sheet-wide empirical outlines of contrasting extent)

CIS 1 (modelled outlines)

NE Asia 3 (regional empirical outline and modelled outlines).

Mean robustness score: 2.5

Supplementary Note 5: 45 ka

The reader should refer to Supplementary Figure 3c for a map of previously published data on ice-sheet extent at 45 ka, Supplementary Figure 3d for a map of the maximum, minimum and best-estimate ice-sheet reconstructions, and Supplementary Table 4 for details of the data sources used to inform these reconstructions.

Maximum estimate of the 45 ka ice-sheet extent

The maximum limit of the empirical data is used for the maximum EIS at 45 ka. It should be noted that, due to the spread of the ages, some of these outlines[3,35,48] probably relate to MIS 4 rather than the peak warmth of MIS 3. This maximum outline accounts for the possibility of a second Weichselian glaciation in Finland at 40–45 ka[168]. A schematic ice cap is shown over Scotland, which is based on the minimum modelled ice extent in Britain during MIS 4[18]. Ice in Greenland and Iceland is shown at the shelf break.

Over North America, the best-estimate LIS at 35 ka, which is based on the empirically derived late MIS 3 outline of Kleman *et al.*[5] and the detailed empirical reconstruction of Dyke *et al.*[2] for 30 ka, is used for the maximum LIS at 45 ka. This outline is extended by around 150 km in the northwest and southeast to include areas covered by ice in hypothesis 2 of Dredge and Thorleifson[42]. The LGM outline of Ehlers *et al.*[93] is used for the CIS. Because of an absence of empirical data, the maximum Quaternary ice-extent template (derived from Glushkova[55] and Barr and Clark[156]) is used for NE Asia.

Minimum estimate of the 45 ka ice-sheet extent

The present-day ice cover is used as the minimum ice extent over Europe, Greenland, the North American Cordillera and NE Asia at 45 ka. Hypothesis 2 of Dredge and Thorleifson[42], which shows small ice-dispersal centres, is used for the LIS. These minimal ice outlines are supported by geochronological work (radiocarbon, OSL) on sub-till sediments from the Hudson Bay Lowlands[159].

Best-estimate of the 45 ka ice-sheet extent

For the best-estimate EIS at 45 ka, we include small ice caps over high areas of Norway and Svalbard. We note that our reconstruction tries to capture the peak warmth of MIS 3, whereas some of the empirical outlines shown for 45 ka may relate to MIS 4[3,35,48] or the suggested expansion of the FIS around 42 ka[8]. Ice in Greenland is shown in a mid-shelf

position, following Funder *et al*.[82] who suggest that the ice sheet was on the continental shelf during this time. Ice in Iceland is shown at the present-day coastline.

Over North America, the minimum LIS extent, which is based on hypothesis 2 of Dredge and Thorleifson[42] is used as the best-estimate. This minimum ice extent is supported by geochronological data from the Hudson Bay Lowlands, Canada, which show the development of peatlands and boreal forests in this region at ~40 ka[159]. Recently, the feasibility of such a reduced ice extent was demonstrated by reconciling geological data from the Hudson Bay Lowlands with estimates of sea level and isostatic adjustment for this area[166]. Coastal mountain glaciers are shown for the CIS, and the LGM ice-extent template of Barr and Clark[156] is used for NE Asia (Methods).

Robustness scores for the 45 ka ice-sheet reconstruction

EIS 3 (ice-sheet-wide empirical outlines of contrasting extent)

LIS 3 (ice-sheet-wide empirical outlines of contrasting extent)

CIS 1 (modelled outlines)

NE Asia 1 (modelled data)

Mean robustness score: 2

Supplementary Note 6: MIS 4 (58–72 ka)

The reader should refer to Supplementary Figure 4a for a map of previously published data on ice-sheet extent during MIS 4, Supplementary Figure 4b for a map of the maximum, minimum and best-estimate ice-sheet reconstructions, and Supplementary Table 5 for details of the data sources used to inform these reconstructions.

<u>Maximum</u> estimate of the MIS 4 (58–72 ka) ice-sheet extent

The maximum empirical data extent is used for the maximum EIS during MIS 4. Ice in Greenland, Iceland and northern Britain is shown at the shelf break. The reconstruction of Helmens[47] is used for the southern margin of the British Irish Ice Sheet (BIIS). Over North America, as the empirical reconstruction of Kleman *et al.*[5] is extrapolated from flow lines and topography, the maximum modelled outline is used for the southern LIS, which is based on Stokes *et al.*[17]. The western margin of the LIS is the same as the maximum reconstruction for MIS 6. The MIS 6 outline is derived from the empirical data of Barendregt *et al.*[88] (modified from Barendregt and Duk-Rodkin[138]), the empirically derived outline of Jackson *et al.*[99] and the modelled outlines of Peltier[108] and de Boer *et al.*[10]. This reconstruction leaves Edmonton ice free during MIS 4, as suggested by Young *et al.*[160]. The maximum Quaternary ice-extent template is used for the maximum CIS during MIS 4. This uses the pre-Reid limit of Kaufman *et al.*[57] in Alaska, the pre-Reid limit of Turner *et al.*[64] in the Yukon, and the MIS 6 modelled outline of Ganopolski and Calov[11] for the southern CIS (see Methods). The Quaternary maximum ice extent of Glushkova[55] and Barr and Clark[156] is used in NE Asia, and extensive grounded ice is shown in the Arctic Ocean[100,105].

<u>Minimum</u> estimate of the MIS 4 (58–72 ka) ice-sheet extent

For the minimum EIS during MIS 4, the minimum empirical ice extent is followed over Scandinavia[59] and the Barents-Kara Sea[48]. Ice is not included in the North Sea, and northwest Denmark is left ice-free after Houmark-Nielsen[3]. The smallest modelled ice extent[18] is shown over Scotland. Ice in Greenland and Iceland is shown to the present-day coastline. The empirically derived outline of Kleman *et al.*[5] is broadly used for the LIS. This minimum ice extent was further reduced in central and eastern Canada by 500–1000 km to account for the possibility of an ice-free interval in these areas. Optically stimulated luminescence dating, uranium-thorium dating and thermoluminescence dating of non-glacial deposits in eastern Canada allow for the possibility that these areas were ice-free during MIS

4[169-174]. The minimum ice extent was further reduced in the Hudson Bay Lowlands by 500–1000 km to account for the possibility of an ice-free interval in that area as indicated by optically stimulated luminescence and uranium-thorium dating on non-glacial materials[175,176]. The LGM ice-extent template is used for the CIS[93] (see Methods). The Quaternary maximum ice-extent template is used for NE Asia, which includes the empirically derived outline of Glushkova[55] for MIS 4.

Best-estimate of the MIS 4 (58–72 ka) ice-sheet extent

The outline of Svendsen *et al.*[62], which is based on a compilation of empirical data, is broadly used for the best-estimate EIS in its northern and western margins during MIS 4. This may correspond with the Ristinge Advance of around 50 ka into eastern Denmark[3,35,48]. Ice is shown in northeast Germany in the best-estimate, as suggested by Möller[60], but we note that this is an area of uncertainty. Where there is a difference between the outlines of Svendsen *et al.*[62] and Mangerud *et al.*[34] in northwest Russia, we follow the more detailed, less extensive reconstruction of Svendsen *et al.*[62]. Glaciation of the Urals[52,62] is included in the MIS 4 best-estimate. The tentative outline of Carr *et al.*[54] is used for the southern margin of the BIIS. The BIIS is extended to the shelf break beyond Scotland as suggested by offshore evidence for ice-sheet expansion during this time[24,71]. Ice is shown in the North Sea[47,54,72]. To be conservative, ice in Greenland is shown in a mid-shelf position, and ice in Iceland is shown at the present-day coastline.

For North America, the empirically derived outline of Kleman *et al.*[5] is used as the best-estimate for the LIS. Empirical data from the Hudson Bay Lowlands are not incorporated into the best-estimate because of low precision of these ages, which leaves the possibility that they may reflect ice-free conditions during MIS 3 or MIS 5a[176]. The Reid ice-extent template of suggested MIS 4/MIS 6 age is used in Alaska (Kaufman *et al.*[57]) and Yukon (Turner *et al.*[64]) (Methods). The Quaternary maximum ice-extent template is used for NE Asia[55,156]. To be conservative, extensive grounded ice is not shown in the Arctic Ocean, but we note that grounded ice may have been present on bathymetric highs[100,105].

Robustness scores for the MIS 4 (58–72 ka) ice-sheet reconstruction

EIS 4 (ice-sheet-wide empirical outlines with some differences in ice extent)

LIS 2 (single broad-scale empirical outline)

CIS 3 (regional empirical outlines)

NE Asia 3 (regional empirical outlines)

Mean robustness score: 3.

Supplementary Note 7: MIS 5a (72–86 ka)

The reader should refer to Supplementary Figure 4c for a map of previously published data on ice-sheet extent during MIS 5a, Supplementary Figure 4d for a map of the maximum, minimum and best-estimate ice-sheet reconstructions, and Supplementary Table 6 for details of the data sources used to inform these reconstructions.

Maximum estimate of the MIS 5a (72–86 ka) ice-sheet extent

For the maximum EIS during MIS 5a, the maximum modelled outline is used but is not allowed to be larger than the best-estimate reconstruction for MIS 5b or 5d. We note that this outline is probably unrealistically extensive. Ice is shown on the continental shelf beyond Scotland to account for the suggestion that ice may have reached beyond the coastline around 80 ka[26]. To cover the maximum scenario, ice is shown to the shelf break beyond Greenland and Iceland. For the maximum LIS during MIS 5a, the modelled outline of Stokes *et al.*[17] is combined with hypothesis 2 of Dredge and Thorleifson[42] for MIS 3. The 30 ka ice-extent template[16] is used for the CIS, and the Quaternary maximum ice-extent template is used for NE Asia[55,156] (see Methods). Extensive grounded ice (following the empirically derived outlines for MIS 6[89,100,105]) is shown in the Arctic Ocean.

Minimum estimate of the MIS 5a (72–86 ka) ice-sheet extent

There is some evidence that global sea level during MIS 5a was close to that of the present-day[178]. To capture this uncertainty, the present-day ice cover is used for the minimum MIS 5a ice extent in Eurasia, Greenland, Iceland and North America. An ice cap, based on hypothesis 2 of Dredge and Thorleifson[42], is also included over Baffin Island.

Best-estimate of the MIS 5a (72–86 ka) ice-sheet extent

For the best-estimate EIS during MIS 5a, the empirically derived outline of Mangerud *et al.*[34] over Norway is combined with the best-estimate 30 ka ice extent (based on Hughes *et al.*[4]) for the islands of the Barents-Kara Sea. To be conservative, no ice is shown in Britain; it is possible that IRD evidence for shelf glaciation during this time[26] may relate to a colder period within MIS 4 or 5. Ice in Greenland and Iceland is shown at the present-day coastline. Hypothesis 2 of Dredge and Thorleifson[42], which is also used as the best-estimate for 45 ka, is followed for the best-estimate LIS during MIS 5a. This ice extent is supported by geochronological data that suggest that large areas of North America were ice-free at this

time[170,171,175]. A schematic outline showing coastal mountain glaciation is used in the North American Cordillera, and the LGM ice-extent template is suggested in NE Asia[156].

Robustness scores for the MIS 5a (72–86) ka ice-sheet reconstruction

EIS 2 (two empirical outlines and modelled outlines)
LIS 1 (modelled outlines; uses ice-sheet extent at 45 ka)
CIS 1 (modelled outlines)
NE Asia 1 (modelled outlines)
Mean robustness score: 1.25

Supplementary Note 8: MIS 5b (86–92 ka)

The reader should refer to Supplementary Figure 5a for a map of previously published data on ice-sheet extent during MIS 5b, Supplementary Figure 5b for a map of the maximum, minimum and best-estimate ice-sheet reconstructions, and Supplementary Table 7 for details of the data sources used to inform these reconstructions.

Maximum estimate of the MIS 5b (86–92 ka) ice-sheet extent

The maximum empirical outline[8,34,53,62,81] is used for the maximum EIS during MIS 5b. Ice is extended to the shelf break in the western Barents Sea, as suggested by Eccleshall *et al.*[67]. A schematic ice cap, based on the minimum modelled ice extent in Britain during MIS 4[18], is shown in Scotland. To cover the maximum scenario, ice in Greenland and Iceland is shown at the shelf break. For the LIS, the maximum modelled outline, which was derived by combining Zweck and Huybrechts[18] and Ganopolski and Calov[11], is used, but is not allowed to be larger than the best-estimate for MIS 4. This is because the global benthic δ^{18}O stack shows MIS 5b to have been significantly warmer than MIS 4[79]. The northwest margin of the LIS is further reduced from the MIS 4 extent in the Mackenzie Delta region, following work that suggests that there were only two ice-sheet advances into this region (probably during either the LGM and MIS 4, or the LGM and MIS 6[179]). The LGM extent of Ehlers *et al.*[93] is shown for the CIS and the maximum Quaternary ice-extent template[55,156] is used for NE Asia. Extensive grounded ice (following the empirically derived outlines for MIS 6[89,100,105]) is shown in the Arctic Ocean.

Minimum estimate of the MIS 5b (86–92 ka) ice-sheet extent

For the minimum EIS during MIS 5b, the minimum empirical outline is used over Europe, and the present-day ice extent is used for Greenland and Iceland. The empirically derived MIS 5b/5d outline of Kleman *et al.*[5] is used for the LIS, together with the present-day ice extent in the islands of the Canadian Arctic. The minimum LIS extent was further reduced in the Gulf of Saint Lawrence and off the coast of Nova Scotia by ~100 to ~200 km to account for the possibility of an ice-free interval in this area as indicated by optically stimulated luminescence and uranium-thorium dating of non-glacial sediments[170,174]. Coastal mountain glaciers are shown for the CIS, and the LGM extent of Barr and Clark[156] is used for NE Asia.

Best-estimate of the MIS 5b (86–92 ka) ice-sheet extent

The empirical outlines of Svendsen *et al*.[62] and Astakhov *et al.*[53] are used for the best-estimate EIS during MIS 5b. The exception is on the western margin of Svalbard, where the ice limit is extended to the shelf break, as suggested by the work of Eccleshall *et al*.[67]. Ice in Greenland is shown in an inner- to mid-shelf position, following the work of Funder *et al*.[82] who suggest that the eastern GIS extended to the Kap Brewster ridge off Scoresby Sund during MIS 5b. Ice in Iceland is extended to the present-day coastline. The minimum ice-sheet reconstruction for MIS 5b is used for the best-estimate in North America. The maximum ice-extent template is used for NE Asia, as suggested by IRSL dates of 80–90 ka on a moraine in this region[50]. To be conservative, grounded ice is not shown in the Arctic Ocean, but we note that grounded ice may have been present on bathymetric highs[100,105].

Robustness scores for the MIS 5b (86–92 ka) ice-sheet reconstruction

EIS 5 (ice-sheet-wide empirical outlines)
LIS 3 (single broad-scale empirical outline)
CIS 1 (modelled outlines)
NE Asia 1 (modelled outlines)
Mean robustness score: 2.5

Supplementary Note 9: MIS 5c (92–108 ka)

The reader should refer to Supplementary Figure 5c for a map of previously published data on ice-sheet extent during MIS 5c, Supplementary Figure 5d for a map of the maximum, minimum and best-estimate ice-sheet reconstructions, and Supplementary Table 8 for details of the data sources used to inform these reconstructions.

<u>Maximum</u> estimate of the MIS 5c (92–108 ka) ice-sheet extent

The maximum empirical outlines in Europe are used for the maximum EIS during MIS 5c. The modelled ice extent of Zweck and Huybrechts[18] for MIS 5a is used for the maximum ice extent on the Putorana Plateau during MIS 5c. This limit is slightly larger than their modelled outline for MIS 5c, and therefore captures a greater range of uncertainty. Ice in Greenland and Iceland is shown to the shelf break. For the maximum LIS, the modelled outline of Stokes *et al.*[17] is combined with hypothesis 2 of Dredge and Thorleifson[42] for MIS 3. The 30 ka ice-extent template[16] is used for the CIS, and the Quaternary maximum ice-extent template[55,156] is used for NE Asia (see Methods). Extensive grounded ice (following the empirically derived outlines for MIS 6[89,100,105]) is shown in the Arctic Ocean.

<u>Minimum</u> estimate of the MIS 5c (92–108 ka) ice-sheet extent

The present-day ice cover is used for the minimum MIS 5c ice extent in Eurasia, Greenland, Iceland and North America. An ice cap, based on hypothesis 2 of Dredge and Thorleifson[42], is also included over Baffin Island.

<u>Best-estimate</u> of the MIS 5c (92–108 ka) ice-sheet extent

The best-estimate for MIS 5a (which uses the MIS 5a empirical outline of Mangerud *et al.*[34] in Norway and the 30 ka ice extent of Hughes *et al.*[4] in the Barents-Kara Sea) is used for the best-estimate EIS during MIS 5c. Ice in Greenland and Iceland is shown at the present-day coastline. Hypothesis 2 of Dredge and Thorleifson[42], which is also used for the best-estimate of 45 ka and MIS 5a, is used for the best-estimate LIS during MIS 5c. This ice extent is supported by geochronological data that suggest that parts of eastern Canada[170,174] and the Hudson Bay Lowlands[175,180] were ice-free during this time. Coastal mountain glaciation is shown in the North American Cordillera, and the LGM ice-extent template[156] is suggested for NE Asia (Methods).

Robustness scores for the MIS 5c (92–108 ka) ice-sheet reconstruction

EIS 3 (ice-sheet-wide empirical outlines of contrasting extent)

LIS 1 (modelled outlines; uses ice-sheet extent during 45 ka)

CIS 1 (modelled outlines)

NE Asia 1 (modelled outlines)

Mean robustness score: 1.5

Supplementary Note 10: MIS 5d (108–117 ka)

The reader should refer to Supplementary Figure 6a for a map of previously published data on ice-sheet extent during MIS 5d, Supplementary Figure 6b for a map of the maximum, minimum and best-estimate ice-sheet reconstructions, and Supplementary Table 9 for details of the data sources used to inform these reconstructions.

Maximum estimate of the MIS 5d (108–117 ka) ice-sheet extent

For the maximum EIS during MIS 5d, the maximum empirical data over western Scandinavia[59] are combined with the maximum modelled outline in eastern Europe and western Siberia[11,76]. This outline is extended slightly farther south in Russia to account for the Kormuzhikhantskaya moraine, which has a suggested age of 100–117 ka[85,181,182]. Ice is shown on the continental shelf beyond Scotland to account for the suggestion that ice may have reached beyond the coastline during this time[26]. To cover the maximum scenario, ice in Greenland and Iceland is shown at the shelf break. For the LIS, the maximum modelled outline, which was derived by combining Ganopolski and Calov[11] and de Boer *et al.*[10], is used, but is not allowed to be larger than the best-estimate for MIS 4. The LGM ice-extent template[93] is shown for the CIS, and the maximum Quaternary ice-extent template[55,156] is used for NE Asia (see Methods). Extensive grounded ice (following the empirically derived outlines for MIS 6[89,100,105]) is shown in the Arctic Ocean.

Minimum estimate of the MIS 5d (108–117 ka) ice-sheet extent

For the minimum EIS during MIS 5d, the smallest empirical outline[47] is used over Scandinavia. The MIS 5c outline of Larsen *et al.*[48], adjusted to incorporate the MIS 5d outline of Möller *et al.*[60], is used for the Barents-Kara Sea. The present-day ice extent is used for Greenland and Iceland. The MIS 5b/5d empirically derived outline of Kleman *et al.*[5] is used for the LIS. The minimum LIS extent was further reduced in the Gulf of Saint Lawrence and off the coast of Nova Scotia by ~100 to 200 km to account for the possibility of an ice-free interval in that area as indicated by optically stimulated luminescence and uranium-thorium dating of non-glacial sediments[170,174]. Coastal mountain glaciers are shown for the CIS, and the LGM ice-extent template[156] is used for NE Asia (Methods).

<u>Best-estimate</u> of the MIS 5d (108–117 ka) ice-sheet extent

For the best-estimate ice sheet in the Barents-Kara Sea, the MIS 5d minimum estimate is combined with the outline of Möller *et al*.[60] for MIS 5d and Astakhov *et al*.[53] for MIS 5b. The ice extent over Scandinavia follows the maximum of the empirical outlines for MIS 5d[8,34,47,59.] To be conservative, the ice sheet is not extended to the Kormuzhikhantskaya moraine in Russia[85,181,182], and no ice is shown in Britain. Ice in Greenland is shown in an inner- to mid-shelf position, following the work of Funder *et al*.[82] who suggest that the eastern GIS extended to the Kap Brewster ridge off Scoresby Sund during MIS 5d. Ice in Iceland is extended to the present-day coastline. The minimum ice-sheet reconstruction for MIS 5d is used for the best-estimate in North America. The maximum ice-extent template[55,156] is used for NE Asia. To be conservative, grounded ice is not shown in the Arctic Ocean, but we note that grounded ice may have been present on bathymetric highs[105,183].

Robustness scores for the MIS 5d (108–117 ka) ice-sheet reconstruction

EIS 3 (ice-sheet-wide empirical outlines of contrasting extent)

LIS 3 (single broad-scale empirical outline)

CIS 1 (modelled outlines)

NE Asia 1 (modelled outlines)

Mean robustness score: 2

Supplementary Note 11: MIS 6 (132–190 ka)

The reader should refer to Supplementary Figure 6c for a map of previously published data on ice-sheet extent during MIS 6, Supplementary Figure 6d for a map of the maximum, minimum and best-estimate ice-sheet reconstructions, and Supplementary Table 10 for details of the data sources used to inform these reconstructions.

Maximum estimate of the MIS 6 (132–190 ka) ice-sheet extent

The maximum empirical data limit is used to define most of the maximum EIS during MIS 6. The empirically derived Samarovo limit of Astakhov *et al.*[53] in eastern Siberia is also included because of uncertainty about the timing of this event (MIS 6 or 8). Ice in Greenland and Iceland is shown at the shelf edge.

Over North America, the southern margin of the LIS is based on the empirical outlines of Balco and Rovey[87] and Curry *et al.*[91]. The western margin of the LIS is defined by the empirical data of Barendregt *et al.*[88] (modified from Barendregt and Duk-Rodkin[138]) the empirically derived outline of Jackson *et al.*[99] and the modelled outlines of Peltier[108] and de Boer *et al.*[10]. This reconstruction leaves Edmonton ice free during this time, as suggested by Young *et al.*[160]. The Quaternary maximum ice-extent templates are used for the CIS[57] and NE Asia[55,156] (see Methods). Extensive grounded ice is shown in the Arctic Ocean[89,100,105,118,183].

Minimum estimate of the MIS 6 (132–190 ka) ice-sheet extent

The minimum empirical data limit is used for the minimum EIS during MIS 6. The Urdachsk and Sampesa moraine limits on the Taymyr Peninsula[60] are not included as these may have been formed during MIS 5b–d. Ice in Greenland and Iceland is shown at the present-day coastline. For the LIS, the empirical outlines of Balco and Rovey[87], Curry *et al.*[91] and Jackson *et al.*[99], are combined with the empirical outline of Barendregt *et al.*[88] (modified from Barendregt and Duk-Rodkin[138]). The LGM ice-extent template is used for the CIS. The Quaternary maximum ice-extent template in NE Asia[55,156] is used to account for the extensive empirically derived ice-sheet outline of Barr and Solomina[41] on the Kamchatka Peninsula. Grounded ice is shown in the Eastern Siberian Sea[89].

Best-estimate of the MIS 6 (132–190 ka) ice-sheet extent

For the best-estimate EIS during MIS 6, the detailed empirical outlines of Ehlers *et al.*[93], Marks[102], Marks *et al.*[104] and Astakhov *et al.*[53] are used, which broadly agree with the

coarser outlines of Svendsen *et al.*[62] and Hughes and Gibbard[98]. The Samarovo limit is not included, since this is more likely to have been reached during MIS 8[53]. The depiction of the GIS at the shelf break is in agreement with work that has inferred extensive glaciation of East Greenland during MIS 6[82,116]. Shelf-break glaciation is also inferred beyond Britain[26,71], Iceland and the Canadian Arctic Archipelago.

Over North America, the empirical data of Balco and Rovey[87], Curry *et al.*[91] and Jackson *et al.*[99], are combined with the coarse ice-sheet outline of Barendregt *et al.*[88] (modified from Barendregt and Duk-Rodkin[138]) for the southern and western LIS margin. The LIS is extended to the shelf break at its southeastern and eastern margin, which is in agreement with modelled outlines[10,11]. For the CIS, the Reid ice-extent template of suggested MIS 4/MIS 6 age is used in Alaska[57] and Yukon[64] (Methods). The maximum Quaternary ice-extent template is used in NE Asia[55,156] to account for the large ice sheet suggested by Barr and Solomina[41] for MIS 6. The maximum inferred extent of grounded ice is shown in the Arctic Ocean[89,100,105,118,183].

Robustness scores for the MIS 6 (132–190 ka) ice-sheet reconstruction

EIS 5 (ice-sheet-wide empirical outlines)
LIS 4 (detailed regional empirical outlines and coarse ice-sheet-wide outline)
CIS 3 (detailed regional empirical outline and coarse ice-sheet-wide outline)
NE Asia 3 (regional empirical outline)
Mean robustness score: 3.75

Supplementary Note 12: MIS 8 (243–279 ka)

The reader should refer to Supplementary Figure 7a for a map of previously published data on ice-sheet extent during MIS 8, Supplementary Figure 7c for a map of the maximum, minimum and best-estimate ice-sheet reconstructions, and Supplementary Table 11 for details of the data sources used to inform these reconstructions.

Maximum estimate of the MIS 8 (243–279 ka) ice-sheet extent

For the maximum EIS during MIS 8, the available empirical outlines in eastern Russia[53,98] are combined with the best-estimate ice-sheet extent during MIS 6 in western Russia and the Barents-Kara Sea. Further to the west, the maximum ice limit includes the Krznanian limit of Marks[103] in Poland, the data of Roskosch *et al.*[106], who suggest two Saalian (MIS 6 and 8) advances into the Leine Valley in Germany, and the data of Beets *et al.*[123] in the North Sea. The maximum Quaternary ice-sheet extent (Anglian Stage limit of MIS 12[95]) is used for Britain, which encompasses the MIS 8 limit suggested by White *et al.*[121, 122]. Shelf-break glaciation is shown for Greenland and Iceland.

Over North America, the northern margin of the LIS is shown at the present-day shelf break. For the southern LIS margin, the maximum of the two modelled outlines of Ganopolski and Calov[11] and de Boer *et al.*[10] is extended to account for the outline of Balco and Rovey[87] that has been suggested for 0.2–0.75 Ma. The maximum reconstruction for MIS 6 is used for the western LIS, which keeps the LIS and CIS separate following the work of Young *et al.*[160]. The maximum Quaternary ice-extent templates are used for the CIS[57] and NE Asia[55,156] (Methods). Extensive grounded ice (following the empirically derived outlines for MIS 6[89,100,105]) is shown in the Arctic Ocean.

Minimum estimate of the MIS 8 (243–279 ka) ice-sheet extent

For the minimum EIS during MIS 8, the Samarovo glaciation limit of Astakhov *et al.*[53] is used in western Siberia and otherwise the minimum estimate for MIS 4 is followed (because of the similar benthic $\delta^{18}O$ records for MIS 4 and 8[79]). To be conservative, this outline is reduced further over Finland and Sweden. Because of uncertainty about the timing of these events, the tentative MIS 8 limits of White *et al.*[121, 122], Marks[103] and Roskosch *et al.*[106] are not included in the minimum reconstruction, and ice is not shown in Britain or in the North Sea. Ice in Greenland and Iceland is shown at the present-day coastline. The

minimum ice-sheet extent for MIS 4 is used for the LIS. Because of an absence of empirical data, the 30 ka ice-extent template[16] is used for the CIS, and no ice is shown in NE Asia.

<u>Best-estimate</u> of the MIS 8 (243–279 ka) ice-sheet extent

Our best-estimate ice-sheet extents for MIS 8 have high uncertainty. They should not be used to indicate the position of the ice-sheet margin, only as an indication of the likely amount of ice present in the NH during this time. For the best-estimate EIS during MIS 8, the empirically derived Samarovo limit of Astakhov *et al*.[53] is used in eastern Europe. Ice is extended to Vologda city, Russia, where it may correlate with the Vologda glaciation[98]. Ice is shown to the shelf break in the Barents-Kara Sea and on the mid-Norwegian shelf[115].

The extent of ice in Britain during MIS 8 is controversial; some researchers suggest that the BIIS reached a similar position to that during the LGM[121,122], whereas others suggest that no unequivocal physical evidence of glaciation during MIS 8 has been identified from the UK[98]. In our best-estimate reconstruction, we show an intermediate-sized ice sheet over Britain that extends to the shelf break beyond Scotland[71] and covers part of the North Sea[72,123,130]. The modelled outline of Ganopolski and Calov[11] is used for the southern and western BIIS margin. To be conservative, ice is not shown extending into central Germany and Poland[103,106] during this time. Ice in Greenland is shown in an intermediate, mid-shelf position, with the exception of part of the western Greenland margin where shelf-break glaciation has been suggested during this time[119]. The minimum ice extent is used for Iceland.

Over North America, for the LIS, the best-estimate for MIS 4 is used (because of the similar benthic δ^{18}O records for MIS 4 and 8[79]), but this is not allowed to be larger than the maximum MIS 8 limit. The exception is the northwest margin of the LIS, where the best-estimate for MIS 6 is used to prevent ice from extending into the Mackenzie Delta region. The range of ages provided for the southern LIS margin by Balco and Rovey[87] span MIS 8, but the three ice advances that are proposed between 0.2 and 0.75 Ma most likely occurred in MIS 6, 12 and 16 because these were the most extensive glaciations in this time span according to the benthic δ^{18}O record[79]. To be conservative, the 30 ka ice-extent template[16] is used for the CIS (although Huscroft *et al*.[184] suggest that the Reid glaciation may date to MIS 8 in some areas). The LGM ice-extent template[156] is used for NE Asia.

Robustness scores for the MIS 8 (243–279 ka) ice-sheet reconstruction

EIS 3 (regional empirical outlines and point-source data)

LIS 2 (small-scale empirical outline; uses ice-sheet extent during MIS 4)

CIS 1 (modelled outline)
NE Asia 1 (modelled outline)
Mean robustness score: 1.75

Supplementary Note 13: MIS 10 (337–365 ka)

The reader should refer to Supplementary Figure 7c for a map of previously published data on ice-sheet extent during MIS 10, Supplementary Figure 7d for a map of the maximum, minimum and best-estimate ice-sheet reconstructions, and Supplementary Table 12 for details of the data sources used to inform these reconstructions.

<u>Maximum</u> estimate of the MIS 10 (337–365 ka) ice-sheet extent

The maximum EIS during MIS 10 includes the empirical data points in western Europe[30,94 97,106]. The maximum reconstruction for MIS 8 is used in Poland, Belarus and western Russia, to account for the suggestion that ice in MIS 10 could have extended as far as the northern foreland of the South Polish Uplands[185]. As the ice-sheet extent during MIS 10 has been suggested to have been smaller than during both MIS 6 and 8[98], the smaller of the best-estimates for MIS 6 and 8 is followed in eastern Europe. Ice in Greenland and Iceland is shown at the present-day shelf break.

Over North America, for the LIS, the maximum modelled extent for MIS 10 is combined with the outline of Balco and Rovey[87]. Ice is shown to the shelf break along the northern and eastern margin of the LIS, as suggested by the modelled outlines of Ganopolski and Calov[11] and de Boer *et al.*[10]. The best-estimate for MIS 12 was followed over Pennsylvania to account for suggestions that this region was ice-covered during either MIS 10 or 12[186-190]. The rest of the southern LIS margin is the maximum modelled ice extent for MIS 10 combined with the empirically derived outline of Balco and Rovey[87]. The maximum reconstruction for MIS 6 is used for the western LIS, which keeps the LIS and CIS separate following the work of Young *et al.*[160]. The maximum Quaternary ice-extent templates are used for the CIS[57] and NE Asia[55,156] (Methods). Extensive grounded ice (following the empirically derived outlines for MIS 6[89,100,107]) is shown in the Arctic Ocean.

<u>Minimum</u> estimate of the MIS 10 (337–365 ka) ice-sheet extent

For the minimum EIS during MIS 10, the minimum reconstruction for MIS 8 is used in Scandinavia and Britain, and the minimum reconstruction for MIS 4 is applied elsewhere. We note that the eastern EIS extent is probably unrealistically small. Ice in Greenland and Iceland is shown at the present-day coastline. The minimum outline for MIS 4 is used for the LIS. The 30 ka ice-extent template[16] is used for the CIS and no ice is shown in NE Asia.

Best-estimate of the MIS 10 (337–365 ka) ice-sheet extent

Because of a lack of empirical data, our best-estimate ice-sheet extents for MIS 10 are highly uncertain. As a result, they should not be used to indicate the position of the ice-sheet margin, only as an indication of the likely amount of ice present in the NH during this time. For the best-estimate EIS reconstruction for MIS 10, the best-estimate for MIS 8 is used for the northern and western limits. To be conservative, ice is not shown extending into central Germany and Poland during this time[94,103,106]. We note that these areas are shown as ice-covered in the maximum reconstruction. In eastern Europe and western Siberia, due to an absence of information, an approximate mid-point between the best-estimates for MIS 8 and 4 is used.

The extent of ice in Britain during MIS 10 is controversial; some researchers have suggested that the BIIS during MIS 10 reached a similar position as during the Elsterian glaciation (MIS 12)[90,97], whereas others have questioned these data, largely because the relationships between dated sand deposits and glacial extents can be ambiguous[98]. In our best-estimate reconstruction, we show an intermediate sized ice sheet over Britain that extends to the shelf break beyond Scotland[71] and covers part of the North Sea[72]. The modelled outline of Ganopolski and Calov[11] is used for the southern and western BIIS margin. Ice in Greenland is shown in a mid-shelf position, with the exception of part of the western Greenland margin where shelf-break glaciation has been suggested during this time[119]. The minimum ice extent is used for Iceland.

Over North America, the best-estimate for MIS 4 is used for the LIS. This is because MIS 4 and MIS 10 have broadly similar values in the benthic δ^{18}O stack[79], although we note that MIS 10 has a lower value than both MIS 4 and MIS 8 and therefore had more ice. The exception is the NW margin of the LIS, where we use the best-estimate for MIS 6 to prevent ice from extending into the Mackenzie Delta region. The range of ages provided for the southern LIS margin by Balco and Rovey[87] spans MIS 10, but the three ice advances that are suggested to have occurred between 0.2 and 0.75 Ma most likely occurred in MIS 6, 12 and 16 because these were the most extensive glaciations in this time span according to the benthic δ^{18}O record[79]. The 30 ka ice-extent template[16] is used for the CIS, and the LGM ice-extent template[156] is used for NE Asia (Methods).

Robustness scores for the MIS 10 (337–365 ka) ice-sheet reconstruction

EIS 2 (regional empirical outlines, point-source data and modelled outlines)

LIS 2 (regional empirical outline and modelled outlines; uses ice-sheet extent during MIS 4)

CIS 1 (modelled outlines)

NE Asia 1 (modelled outlines)

Mean robustness score: 1.5

Supplementary Note 14: MIS 12 (429–477 ka)

The reader should refer to Supplementary Figure 8a for a map of previously published data on ice-sheet extent during MIS 12, Supplementary Figure 8b for a map of the maximum, minimum and best-estimate ice-sheet reconstructions, and Supplementary Table 13 for details of the data sources used to inform these reconstructions.

Maximum estimate of the MIS 12 (429–477 ka) ice-sheet extent

The maximum empirical outlines over Europe are followed for the maximum EIS during MIS 12. The outline of Eissmann[94], which shows part of the Don lobe as MIS 12, is included. The southern limit, between the Urals and the empirical outline of Astakhov *et al.*[53], is the all-time Quaternary maximum, which, in this region, is the maximum reconstruction of MIS 8. Ice is extended to the shelf break in the Barents-Kara Sea, off Norway and beyond Greenland and Iceland. For the LIS, the maximum reconstruction for MIS 6 is used, which includes the empirically derived outline of Balco and Rovey[87]. The maximum Quaternary ice-extent templates are used for the CIS[57] and NE Asia[55,156] (Methods). Extensive grounded ice (following the empirically derived outlines for MIS 6[89,100,105]) is shown in the Arctic Ocean.

Minimum estimate of the MIS 12 (429–477 ka) ice-sheet extent

For the minimum EIS and LIS during MIS 12, empirical outlines[87,93,95,96,101,104] are used where available and the minimum reconstruction of MIS 6 is used where there are gaps in empirical data coverage. Ice in Greenland and Iceland is shown at the present-day coastline. The 30 ka ice-extent template[16] is used for the CIS, and the LGM ice-extent template[156] is used in NE Asia (Methods).

Best-estimate of the MIS 12 (429–477 ka) ice-sheet extent

The detailed empirical outlines of Gibbard and Clark[95] in Britain, Laban and van der Meer[101] in the Netherlands, and Ehlers *et al.*[93] in Germany are followed for the best-estimate EIS during MIS 12. The empirical outlines of Marks *et al.*[104] and Gozhik *et al.*[96] are adopted in eastern Europe, and the outline of Astakhov *et al.*[53] is followed in central Siberia. Due to the lack of empirical data between these regions, the best-estimate of MIS 6 is used to delimit the southern margin of the EIS. Ice in Greenland and Iceland is shown at the shelf break. The best-estimate for MIS 6 is used for the LIS, which includes the outline of Balco and Rovey[87].

The LGM ice-extent template is used for the CIS, and the maximum Quaternary ice-extent template[55,156] is used for NE Asia (Methods).

Robustness scores for the MIS 12 (429-477 ka) ice-sheet reconstruction

EIS 4 (many regional empirical outlines)

LIS 2 (regional empirical outline; uses ice-sheet extent from MIS 6)

CIS 1 (modelled outline; LGM empirically derived template)

NE Asia 1 (modelled outline)

Mean robustness score: 2

Supplementary Note 15: MIS 16 (622–677 ka)

The reader should refer to Supplementary Figure 8c for a map of previously published data on ice-sheet extent during MIS 16, Supplementary Figure 8d for a map of the maximum, minimum and best-estimate ice-sheet reconstructions, and Supplementary Table 14 for details of the data sources used to inform these reconstructions.

<u>Maximum</u> estimate of the MIS 16 (622–677 ka) ice-sheet extent

For the maximum EIS during MIS 16, the maximum empirical data[53,96,98] are used for the southern ice-sheet margin. The maximum reconstruction for MIS 12 is used for Britain, western Scandinavia and the Barents-Kara Sea because of the similar benthic $\delta^{18}O$ records for MIS 16 and 12[79]. This incorporates the empirical data of Hamblin *et al.*[97] in Britain, and the suggestion of shelf-break glaciation on the mid-Norwegian margin[115]. Ice is shown at the shelf break beyond Greenland and Iceland. Ice is also shown at the shelf break along the northern and eastern margin of the LIS. The southern LIS follows the empirical data for MIS 16[87,128], and the western margin uses the maximum reconstructed ice limit for MIS 6. The maximum Quaternary ice-extent templates are used for the CIS[57] and NE Asia[55,156] (Methods). Extensive grounded ice (following the empirically derived outlines for MIS 6[89,100,105]) is shown in the Arctic Ocean.

<u>Minimum</u> estimate of the MIS 16 (622–677 ka) ice-sheet extent

For the minimum EIS during MIS 16, we use the empirically derived outlines of Olsen *et al.*[8] in Scandinavia and the Barents-Kara Sea, Toucanne *et al.*[130] in Denmark, Germany and Poland, and Astakhov *et al.*[53] in Russia. Note that our minimum reconstruction for the EIS in Siberia shows virtually no ice, which is likely to be unrealistic even for a minimum estimate. To be conservative in our minimum estimate, grounded ice is not included in the North Sea. Schematic ice caps are shown over Scotland and Ireland, as in the minimum reconstruction for MIS 20–24. Ice in Greenland and Iceland is shown at the present-day coastline. For the minimum LIS during MIS 16, the minimum empirical data[129] is used where they are available, and the minimum of MIS 6 is used where empirical data for MIS 16 are lacking. Owing to a lack of data, the 30 ka ice-extent template[16] is used for the CIS, and the LGM ice-extent template[156] is used in NE Asia (Methods).

<u>Best-estimate</u> of the MIS 16 (622–677 ka) ice-sheet extent

For the best-estimate EIS for MIS 16, the empirically derived outlines of Toucanne *et al.*[130], Gozhik *et al.*[96], Astakhov *et al.*[53] and Marks *et al.*[104] are used for the southern limit in Europe. East of the Urals, in western Siberia, the best-estimate of MIS 6 is used to provide a realistic ice-sheet extent given the relatively extensive glaciation of Russia during MIS 16[53]. The EIS is extended to the shelf break on the mid-Norwegian margin[115]. Further, our best-estimate for MIS 16 shows the EIS extending into the central North Sea, as in the best-estimate for MIS 20–24. The EIS and BIIS are not joined during this time, following the suggestion of Toucanne *et al.*[130]. Ice in Greenland and Iceland is shown at the shelf break.

To produce the best-estimate of MIS 16 ice over North America for the LIS, the empirical outlines of Aber[128] and Colgan[129] are used to delimit the southern extent. For the remainder, the best-estimate from MIS 6 is used. In western North America, the LGM ice-extent template is used for the CIS[93], and the maximum Quaternary ice-extent template[55,156] is adopted for NE Asia (Methods).

Robustness scores for the MIS 16 (622–677 ka) ice-sheet reconstruction

EIS 4 (regional empirical outlines; uses ice-sheet extents from MIS 6 and 12)

LIS 4 (regional empirical outlines; uses ice-sheet extents from MIS 6 and 12)

CIS 0 (no data)

NE Asia 0 (no data)

Mean robustness score: 2

Supplementary Note 16: MIS 20–24 (790–928 ka)

The reader should refer to Supplementary Figure 9a for a map of previously published data on ice-sheet extent during MIS 20–24, Supplementary Figure 9b for a map of the maximum, minimum and best-estimate ice-sheet reconstructions, and Supplementary Table 15 for details of the data sources used to inform these reconstructions.

Maximum estimate of the MIS 20–24 (790–928 ka) ice-sheet extent

For the maximum EIS during MIS 20–24, tentative empirical outlines for the Nidanian glaciation[96,103] are merged with the maximum estimates of MIS 8 and 10 combined (because of the similar benthic $\delta^{18}O$ values for MIS 8, 10 and 20–24[79]). Ice in Greenland and Iceland is shown to the shelf break. Over North America, for the LIS, the outline of Balco and Rovey[87] is combined with the maximum estimate for MIS 8 and 10. Over western North America and NE Asia, to capture the maximum scenario, the maximum Quaternary ice-extent templates are used for the CIS[57] and in NE Asia[55,156] (see Methods). Finally, extensive grounded ice (following the empirically derived outlines for MIS 6[89,100,105]) is shown in the Arctic Ocean.

Minimum estimate of the MIS 20–24 (790–928 ka) ice-sheet extent

For the minimum EIS during MIS 20–24, we use the same ice-sheet extent as for the minimum ice-sheet reconstruction for the early Matuyama magnetic Chron (1.78–2.6 Ma). The minimum reconstruction for the early Matuyama Chron follows the smaller of the two empirically derived reconstructions of Knies *et al.*[141] over the Barents-Kara Sea, and the minimum of the empirical outlines[132,139,141] for the early Matuyama Chron over Scandinavia. Schematic ice caps are shown over Scotland and Ireland to account for IRD evidence for marine-terminating glaciers during this time[137]. Ice in Greenland and Iceland is shown at the present-day coastline. Over North America, the reconstruction of Andriashek and Barendregt[131] is used for the LIS, and the 30 ka ice-extent template[16] is used for the CIS. Finally, no ice is shown in NE Asia or the Arctic Ocean.

Best-estimate of the MIS 20–24 (790–928 ka) ice-sheet extent

Our MIS 20–24 time-slice spans part of the Mid-Pleistocene Transition, which was a time of generally expanded NH ice sheets. In our MIS 20–24 best-estimate reconstruction, the EIS is extended into central Europe to incorporate the suggested outlines for the Nidanian

glaciation of around 0.9 Ma[96,103]. At this time, we also interpolate an ice margin between Scandinavia and central Europe, linking the limit of Olsen *et al.*[8] with those of Gozhik *et al.*[96] and Marks[103]. The expansion of the EIS into central Europe around 1 Ma has been inferred to have led to excavation of the Baltic Basin, causing the Baltic (Eridanos) river system, which had operated in the Miocene, Pliocene and Early Pleistocene, to lose its connection to the Scandinavian and Baltic headwaters[191,192]. The empirically derived outline of Olsen *et al.*[8] is used for the best-estimate ice sheet in the Barents-Kara Sea. The EIS is extended to the shelf break off western Norway[115] and into the central North Sea[132,133,136]. We note that there is also evidence for the FIS extending into the central North Sea slightly earlier, around 1.1–1.2 Ma[193,194]. To be conservative, ice in Iceland is shown at the present-day coastline, and the minimum ice-sheet extent is used for Britain and Ireland. The reconstruction of the GIS at the shelf break during MIS 20–24 is in agreement with an increase in IRD at around 0.8 Ma[134], and seismic evidence for multiple cross-shelf glaciations between 0.78 and 1.77 Ma[135].

To produce the best-estimate reconstruction of MIS 20–24 ice over North America, the LIS reconstruction of Andriashek and Barendregt[131] is combined with the best-estimate for MIS 8 at the southeast and northeast ice-sheet margin. This is because the reconstruction of Andriashek and Barendregt[131] is a schematic outline around sites that they interpret to have been covered by ice as suggested by palaeo-magnetic dating, and is therefore a minimum extent. Our best-estimate of MIS 20–24 ice also smooths an irregular ice margin in the southwest by extending the outline by about 100 km. The minimum reconstruction is followed for the northwest LIS in the Mackenzie Delta region. To account for the relatively large empirically derived outline of Andriashek and Barendregt[131], the Reid ice-sheet template of MIS 4/6 is used for the CIS[57,64]. Finally, the LGM ice-extent template[156] is shown in NE Asia (Methods) and no ice is shown in the Arctic Ocean.

Robustness scores for the MIS 20–24 (790–928 ka) ice-sheet reconstruction

EIS 3 (regional empirical outlines of contrasting extent)

LIS 3 (regional empirical outline and coarse ice-sheet-wide outline; uses ice-sheet extent from MIS 8)

CIS 3 (coarse ice-sheet-wide outline and empirical data points)

NE Asia 0 (no data)

Mean robustness score: 2.25

Supplementary Note 17: Early Matuyama palaeomagnetic Chron (1.78–2.6 Ma)

The reader should refer to Supplementary Figure 9c for a map of previously published data on ice-sheet extent during the early Matuyama palaeomagnetic Chron, Supplementary Figure 9d for a map of the maximum, minimum and best-estimate ice-sheet reconstructions, and Supplementary Table 16 for details of the data sources used to inform these reconstructions.

<u>Maximum</u> estimate of the early Matuyama palaeomagnetic Chron (1.78–2.6 Ma) ice-sheet extent

Our reconstructions aim to show the *maximum* extent of the NH ice sheets within the long (0.8 Ma) period of the early Matuyama Chron (Methods). For the maximum EIS during this period, the best-estimate reconstruction for MIS 20–24 is used in most cases; this includes the proposed extent of the Narewian glaciation of Germany and Poland[103], which has been suggested to be *c*.1.4 Ma in age, because of uncertainty in dating older sediments. However, the maximum outline differs from the MIS 20–24 reconstruction in the North Sea. For the maximum ice extent in the early Matuyama Chron, we show the EIS extending westward into the northern North Sea, but it is not merged with the BIIS because the central North Sea was a deep basin during the Early Pleistocene[133]. We also show an ice sheet over Scotland and Ireland to account for IRD and seismic evidence for marine-terminating glaciers during this time[137,142]. Ice in Greenland and Iceland is shown to the shelf break.

Over North America, for the LIS, the empirical data of Balco and Rovey[87] is combined with the maximum reconstruction for MIS 6. Over western North America, the lack of available evidence means that the maximum Quaternary ice-extent templates are used for the CIS[57] and NE Asia[55,156] (Methods). Finally, extensive grounded ice (following the empirically derived outlines for MIS 6[89,100,105]) is shown in the Arctic Ocean.

<u>Minimum</u> estimate of the early Matuyama palaeomagnetic Chron (1.78–2.6 Ma) ice-sheet extent

For the minimum EIS during the early Matuyama magnetic Chron, the smaller of the two empirically derived outlines of Knies *et al.*[141] is used for the islands of the Barents-Kara Sea. The minimum empirical outlines[132,139,141] are used over Scandinavia, which show the ice sheet extending to the present-day coastline. Schematic ice caps are shown over Scotland and

Ireland to account for IRD evidence for marine-terminating glaciers during this time[137]. Ice in Greenland and Iceland is shown at the present-day coastline. Over North America, for the LIS, the empirically derived outlines of Balco and Rovey[87] and Barendregt *et al*.[88] (modified from Barendregt and Duk-Rodkin[138]) are used. The 30 ka ice-extent template[16] is used for the CIS and no ice is shown in NE Asia (Methods).

Best-estimate of the early Matuyama palaeomagnetic Chron (1.78–2.6 Ma) ice-sheet extent

For the best-estimate EIS during the early Matuyama Chron, we use the larger of the two empirically derived outlines of Knies *et al*.[141] in the Barents-Kara Sea and northern Scandinavia. This follows evidence that the Barents-Kara Ice Sheet developed to a moderate size during a transitional growth phase between around 2.4 and 1 Ma[141,146,150,195-197]. The outlines of Knies *et al*.[141], Ottesen *et al.*[133] and Rea *et al*.[142] are followed in southern Scandinavia and the North Sea. Ice is extended to the present-day shelf break on the mid-Norwegian margin, although we note that the shelf break has prograded several tens of kilometres in a seaward direction through the Quaternary[115]. To be conservative, the minimum ice-sheet extent is used over Britain, which shows ice in Scotland and Ireland reaching sea level[137].

The GIS is shown at the shelf break in our best-estimate reconstruction for the early Matuyama Chron. This follows seismic stratigraphic investigations and modelling studies that suggest that the GIS extended to the shelf break during the Late Pliocene to Early Pleistocene, between around 2.5 and 3 Ma[116,134,143,145,148,150,198,199]. An expanded GIS during this time is also suggested from IRD records[144,149]. The GIS probably advanced to the shelf break during several glacial periods within the early Matuyama palaeo-magnetic Chron[135]. Although our best-estimate reconstruction aims to capture the *maximum* ice-sheet extent within this long timeslice, we note that there is evidence for a reduced GIS during an Early Pleistocene warm period around 2.4 Ma[200,201].

Over North America, for the LIS, we adopt the best-estimate for MIS 4 combined with the empirical outlines of Balco and Rovey[87] and Barendregt *et al*.[88] (modified from Barendregt and Duk-Rodkin[138]). The maximum Quaternary ice-extent template is used for the CIS[57], following suggestions that the CIS reached its maximum extent during the early Matuyama palaeo-magnetic Chron[88,138]. Because of the lack of data for NE Asia during this time-slice, the best-estimate uses the LGM ice-sheet template[156], which is a mid-point between our minimum and maximum reconstructions.

Robustness scores for the early Matuyama palaeomagnetic Chron (1.78–2.6 Ma) ice-sheet reconstruction

EIS 3 (regional empirical outlines of contrasting extent)

LIS 3 (regional empirical outline and coarse ice-sheet-wide empirical outline; uses ice-sheet extent of MIS 4)

CIS 3 (coarse ice-sheet-wide outline and empirical data points)

NE Asia 0 (no data)

Mean robustness score: 2.25

Supplementary Note 18: Late Gauss palaeomagnetic Chron (2.6–3.59 Ma)

The reader should refer to Supplementary Figure 10a for a map of previously published data on ice-sheet extent during the late Gauss palaeomagnetic Chron, Supplementary Figure 10b for a map of the maximum, minimum and best-estimate ice-sheet reconstructions, and Supplementary Table 17 for details of the data sources used to inform these reconstructions.

<u>Maximum</u> estimate of the late Gauss palaeomagnetic Chron (2.6–3.59 Ma) ice-sheet extent

In this section, it should be noted that we show the *maximum* ice extent during the late Gauss magnetic Chron (2.6–3.59 Ma) that probably dates to around 2.6 Ma, when NH glaciations became more extensive. Over northern Europe and the Barents-Kara Sea, the larger of the hypothesised outlines of Knies *et al.*[141] are used. Our maximum ice outline for Scandinavia is also extended westward into the northern North Sea, following evidence that the FIS had a marine-terminating margin since around 2.7 Ma[133]. An ice sheet is shown over Scotland and Ireland to account for IRD evidence for marine-terminating glaciers[137]. Ice in Greenland and Iceland is shown to the shelf break.

Over North America, for the LIS, the generalised schematic outline of Kleman *et al.*[5] for 35 ka and 40 ka is used for this maximum outline. Also included in this outline are the empirical data from hypothesis 2 of Dredge and Thorleifson[42] for MIS 3, which show ice cover over Nova Scotia and northwestern Canada. For the CIS, because of an absence of empirical data, the maximum Quaternary ice-extent template is used, which is based mainly on Kaufman *et al.*[57] and Turner *et al.*[64] (Methods), to account for the large empirically derived outline of Barendregt *et al.*[88] (modified from Barendregt and Duk-Rodkin[138]). The maximum Quaternary ice-extent template is used in NE Asia[55,156]. Finally, the maximum reconstruction shows extensive grounded ice (following the empirically derived outlines for MIS 6[89,100,105]) in the Arctic Ocean.

<u>Minimum</u> estimate of the late Gauss palaeomagnetic Chron (2.6–3.59 Ma) ice-sheet extent

For the minimum EIS during the late Gauss palaeo-magnetic Chron, the present-day ice extent is combined with schematic ice caps in Norway and the Barents-Kara Sea (based on Hughes *et al.*[4] for 35 ka). The present-day ice extent is also adopted for Greenland,

Iceland and the LIS. The 30 ka ice-extent template[16] is used for the CIS and no ice is shown in NE Asia or the Arctic Ocean.

<u>Best-estimate</u> of the late Gauss palaeomagnetic Chron (2.6–3.59 Ma) ice-sheet extent

For the best-estimate EIS during the late Gauss palaeo-magnetic Chron, the minimum reconstruction of Knies *et al*.[141] is used in northern Norway and the Barents-Kara Sea. This outline is adjusted slightly to cover our minimum reconstruction for the late Gauss palaeomagnetic Chron, which is based on the schematic ice caps of Hughes *et al*.[4] for 35 ka. Following seismic and IRD evidence for a marine-terminating ice margin at around 2.7 Ma[149,202], we also extend this ice margin to the coastline of mid-Norway. The FIS is also extended into the northern North Sea, following evidence of a marine-terminating ice margin from around 2.7 Ma[133]. This evidence includes features interpreted as glacigenic debris-flow deposits on palaeo-slope surfaces[132,203], IRD in sediment cores[202,204], and iceberg ploughmarks preserved on early Quaternary surfaces[139,142]. Our best-estimate FIS is shown at the former shelf break in the northern North Sea, which was located around 80 km beyond the present-day coastline during the Late Pliocene/ Early Pleistocene when the North Sea was a deep basin[133]. The best-estimate reconstruction also shows ice caps over Scotland and Ireland to account for IRD evidence for marine-terminating glaciers during this time[137].

The GIS is shown at the shelf break in our best-estimate reconstruction. This is in agreement with empirical and modelling work that suggests that the GIS extended to the shelf break during the Late Pliocene to Early Pleistocene, between around 2.5 and 3 Ma[116,134,143,145,148,150,198,199]. An expanded GIS during this time is also suggested from IRD records[144,149]. It is noted that the late Gauss palaeo-magnetic Chron spans a period of Early Pliocene warmth (5.5–3 Ma), during which there is evidence for a reduced GIS[205-207]. To be conservative, the present-day ice extent is shown for Iceland.

Over North America, for the LIS, the best-estimate for 45 ka is used (based on hypothesis 2 of Dredge and Thorleifson[42]) and shows the main ice dispersal centres. Due to suggestions of an extensive CIS during this time[88,138,147], the maximum Quaternary ice-extent template[57] is used for the CIS. The LGM ice-extent template[156] is used for NE Asia, which is a mid-point between our maximum and minimum reconstructions, and accounts for IRD evidence that glaciers on the Kamchatka Peninsula reached at least sea level around 2.6 Ma[125,208].

Robustness scores for the late Gauss palaeomagnetic Chron (2.6–3.59 Ma) ice-sheet reconstruction

EIS 2 (regional empirical outlines)

LIS 0 (no data)

CIS 3 (coarse ice-sheet-wide outline and empirical data points)

NE Asia 0 (no data)

Mean robustness score: 1.25

Supplementary References

1. Amante, C. & Eakins, B. W. ETOPO1 1 Arc-Minute Global Relief Model: Procedures, Data Sources and Analysis. *NOAA Technical Memorandum NESDIS NGDC-24*. National Geophysical Data Center, NOAA (2009).

2. Dyke, A. S., Andrews, J. T., Clark, P. U., England, J. H., Miller, G. H., Shaw, J. & Veillette, J. J. The Laurentide and Innuitian ice sheets during the Last Glacial Maximum. *Quat. Sci. Rev.* **21**, 9–31 (2002).

3. Houmark-Nielsen, M. Extent, age and dynamics of Marine Isotope Stage 3 glaciations in the southwestern Baltic Basin. *Boreas* **39**, 343–359 (2010).

4. Hughes, A. L. C., Gyllencreutz, R., Lohne, Ø. S., Mangerud, J. & Svendsen, J. I. The last Eurasian ice sheets–a chronological database and time-slice reconstruction, DATED-1. *Boreas* **45**, 1–45 (2016).

5. Kleman, J., Jansson, K., De Angelis, H., Stroevem, A. P., Hättestrand, C., Alm, G. & Glasser, N. North American ice sheet build-up during the last glacial cycle, 115-21 kyr. *Quat. Sci. Rev.* **29**, 2036–2051 (2010).

6. Larsen, N. K., Knudsen, K. L., Krohn, C. F., Knonborg, C., Murray, A. S. & Nielsen, O. B. Late Quaternary ice sheet, lake and sea history of southwest Scandinavia – a synthesis. *Boreas* **38**, 732–761 (2009).

7. Marks, L. Timing of the Late Vistulian (Weichselian) glacial phases in Poland. *Quat. Sci. Rev.* **44**, 81–88 (2012).

8. Olsen, L., Sveian, H., Bergstrøm, B., Ottesen, D. & Rise, L. Quaternary glaciations and their variations in Norway and on the Norwegian continental shelf. In: Olsen, L., Fredin, O. & Olesen, O. (Eds.) Quaternary Geology of Norway, *Geological Survey of Norway Special Publication* **13**, 27–78 (2013).

9. Bonelli, S., Charbit, S., Kageyama, M., Woillez, M. -N., Ramstein, G., Dumas, C. & Quiquet, A. Investigating the evolution of major NH ice sheets during the last interglacial cycle. *Clim. Past* **5**, 329–345 (2009).

10. de Boer, B., Stocchi, P. & van de Wal, R. S. W. A fully coupled 3-D ice-sheet–sea-level model: algorithm and applications. *Geosci. Model Dev.* **7**, 2141–2156 (2014).

11. Ganopolski, A. & Calov, R. The role of orbital forcing, carbon dioxide and regolith in 100 kyr glacial cycles. *Clim. Past* **7**, 1415–1425 (2011).

12. Heinemann, M., Timmermann, A., Timm, O. E., Saito, F. & Abe-Ouchi, A. Deglacial ice sheet meltdown: orbital pacemaking and CO_2 effects. *Cim. Past* **10**, 1567–1579 (2014).

13. Hubbard, A., Bradwell, T., Golledge, N., Hull, A., Patton, H., Sugden, D., Cooper, R. & Stoker, M. Dynamic cycles, ice streams and their impact on the extent, chronology and deglaciation of the British-Irish ice sheet. *Quat. Sci. Rev*. **28**, 758–776 (2009).

14. Lambeck, K., Purcell, A., Zhao, J. & Svendsen, N-O. The Scandinavian Ice Sheet; from MIS 4 to the end of the Last Glacial Maximum. *Boreas* **39**, 410–435 (2010).

15. Patton, H., Hubbard, A., Bradwell, T. & Schomacker, A. The configuration, sensitivity and rapid retreat of the Late Weichselian Icelandic ice sheet. *Earth-Sci. Rev.* **166**, 223–245 (2017).

16. Seguinot, J., Rogozhina, I., Stroeven, A. P., Margold, M. & Kleman, J. Numerical simulations of the Cordilleran ice sheet through the last glacial cycle. *Cryosphere* **10**, 639–664 (2016).

17. Stokes, C. R., Tarasov, L. & Dyke, A. S. Dynamics of the North American Ice Sheet complex during its inception and build-up to the Last Glacial Maximum. *Quat. Sci. Rev.* **50**, 86–104 (2012).

18. Zweck, C. & Huybrechts, P. Modeling of the NH ice sheets during the last glacial cycle and glaciological sensitivity. *J. Geophys. Res.* **110**, D07103 (2005).

19. Abramowski, U., Bergau, A., Seebach, D., Zech, R., Glaser, B., Sosin, P., Kubin, P. W. & Zech, W. Pleistocene glaciations of Central Asia: results from ^{10}Be surface exposure ages of erratic boulders from the Pamir (Tajikistan), and the Alay-Turkestan range (Kyrgyzstan). *Quat. Sci. Rev.* **25**, 1080–1096 (2006).

20. Arzhannikhov, S. G., Braucher, R., Jolivet, M. & Arzhannikova, A. V. Late Pleistocene glaciations in southern East Sayan and detection of MIS 2 terminal moraines based on beryllium (^{10}Be) dating of glacier complexes. *Russ. Geol. Geophys.* **56**, 1509–1521 (2015).

21. Baumann, K-H., Lackschewitz, K. S., Mangerud, J., Spielhagen, R. F., Wolf-welling, T. C. W., Henrich, R. & Kassens, H. Reflection of Scandinavian Ice Sheet fluctuations in Norwegian Sea sediments during the past 150,000 years. *Quat. Res.* **43**, 185–197 (1995).

22. Chevalier, M-L., Hilley, G., Tapponnier, P., Van Der Woerd, J., Liu-Zeng, J., Finkel, R. C., Ryerson, F. J., Li, H. & Liu, X. Constraints on the late Quaternary glaciations

in Tibet from cosmogenic exposure ages of moraine surfaces. *Quat. Sci. Rev*. **30**, 528–554 (2011).

23. Hall, A. M. The last glaciation of Shetland: local ice cap or invasive ice sheet? *Norwegian Journal of Geology* **93**, 229–242 (2013).

24. Hibbert, F. D., Austin, W. E. N., Leng, M. J. & Gatliff, R. W. British Ice Sheet dynamics inferred from North Atlantic ice-rafted debris records spanning the last 175 000 years. *J. Quat. Sci.* **25**, 461–482 (2010).

25. Lehmkuhl, F. Quaternary glaciations in central and western Mongolia. *Quaternary Proceedings* **6**, 153–167 (1998).

26. Lekens, W. A. H., Haflidason, H., Sejrup, H. P., Nygard, A., Richter, T., Vogt, C. & Frederichs, T. Sedimentation history of the northern North Sea Margin during the last 150 ka. *Quat. Sci. Rev*. **28**, 469–483 (2009).

27. Li, Y. *et al*. Timing and extent of Quaternary glaciations in the Tianger Range, eastern Tian Shan, China, investigated using ^{10}Be surface exposure dating. *Quat. Sci. Rev.* **98**, 7–23 (2014).

28. Owen, L. A. & Dortch, J. M. Nature and timing of Quaternary glaciation in the Himalayan-Tibetan orogen. *Quat. Sci. Rev.* **88**, 14–54 (2014).

29. Owen, L. A., Yi, C., Finkel, R. C. & Davis, N. K. Quaternary glaciation of Gurla Mandhata (Naimon'anyi). *Quat. Sci. Rev.* **29**, 1817–1830 (2010).

30. Stein, R., Nam, S., Grobe, H. & Hubberten, H. Late Quaternary glacial history and short-term ice-rafted debris fluctuations along the East Greenland continental margin. *Geological Society, London, Special Publications* **111**, 135–151 (1996).

31. Stübner, K., Grin, E., Hidy, A. J., Schaller, M., Gold, R. D., Ratschbacher, L & Ehlers, T. Middle and Late Pleistocene glaciations in the southwestern Pamir and their effects on topography. *Earth Planet. Sci. Lett.* **466**, 181–194 (2017).

32. Thackray, G. D. Varied climatic and topographic influences on Late Pleistocene mountain glaciation in the western United States. *J. Quat. Sci.* **23**, 671–681 (2008).

33. Arnold, N. S., van Andel, T. H. & Valen, V. Extent and dynamics of the Scandinavian Ice Sheet during Oxygen Isotope Stage 3 (65,000-25,000 yr B.P.) *Quat. Res.* **57**, 38–48 (2002).

34. Mangerud, J., Gyllencreutz, R., Lohne, Ø. & Svendsen, J. I. Glacial History of Norway. In: Ehlers, J., Gibbard, P. L. & Hughes, P. D. (eds). Quaternary Glaciation Extent and Chronology: a closer look. *Developments in Quaternary Science* **15**, Elsevier, Amsterdam (2011).

35. Obst, K., Nachtweide, C. & Müller, U. Late Saalian and Weichselian glaciations in the German Baltic Sea documented by Pleistocene successions at the southeastern margin of the Arkona Basin. *Boreas* **46**, 18–33 (2017).

36. Murton, D. K. Late Pleistocene palaeoenvironmental change in the Vale of York and Humber gap. PhD thesis, University of Cambridge (2017).

37. Owen, L., Finkel, R. C., Haizhou, M., Spencer, J. Q., Derbyshire, E., Barnard, P. L. & Caffee, M. W. Timing and style of Late Quaternary glaciation in northeastern Tibet. *Geol. Soc. Am. Bull.* **115**, 1356–1364 (2003).

38. Owen, L. A., Robinson, R., Benn, D. I., Finkel, R. C., Davis, N. K., Yi, C., Putkonen, J., Li, D. & Murray, A. S. Quaternary glaciation of Mount Everest. *Quat. Sci. Rev.* **28**, 1412–1433 (2009).

39. Rother, H., Lehmkuhl, F., Fink, D. & Nottebaum, V. Surface exposure dating reveals MIS-3 glacial maximum in the Khangai Mountains of Mongolia. *Quat. Res.* **82**, 297–308 (2014).

40. Syvitski, J. P., Jennings, A. E. & Andrews, J. T. High-resolution seismic evidence for multiple glaciation across the Southwest Iceland shelf. *Arct. Antarct. Alp. Res.* **31**, 50–57 (1999).

41. Barr, I. D. & Solomina, O. Reprint of 'Pleistocene and Holocene glacier fluctuations upon the Kamchatka Peninsula'. *Glob. Planet. Change* **134**, 155–165 (2015).

42. Dredge, L. A. & Thorleifson, L. H. The Middle Wisconsinan history of the Laurentide Ice Sheet. *Géographie physique et Quaternaire* **41**, 215–235 (1987).

43. Van Andel, T. H. & Tzedakis, P. C. Palaeolithic landscapes of Europe and environs, 150,000-25,000 years ago: An overview. *Quat. Sci. Rev.* **15**, 481–500 (1996).

44. Marshall, S. J., Tarasov, L., Clarke, G. K. C. & Peltier, W. R. Glaciological reconstruction of the Laurentide Ice Sheet: physical processes and modelling challenges. *Can. J. Earth Sci.* **37**, 769–793 (2000).

45. Zhao, J., Liu, S., He, Y. & Song, Y. Quaternary glacial chronology of the Ateaoyinake River Valley, Tianshan Mountains, China. *Geomorphology* **103**, 276–284 (2009).

46. Zhao, J., Yin, X., Harbor, J. M., Lai, Z., Liu, S. & Li, Z. Quaternary glacial chronology of the Kansas River valley, Altai Mountains, China. *Quat. Int.* **311**, 44–53 (2013).

47. Helmens, K. F. The Last Interglacial-Glacial cycle (MIS 5-2) re-examined based on long proxy records from central and northern Europe. *Quat. Sci. Rev.* **86**, 115–143 (2014).

48. Larsen, E. *et al*. Late Pleistocene glacial and lake history of northwestern Russia. *Boreas* **35**, 394–424 (2006).

49. Owen, L. A., Caffee, M. W., Bovard, K. R., Finkel, R. C. & Sharma, M. C. Terrestrial cosmogenic nuclide surface exposure dating of the oldest glacial successions in the Himalayan orogen: Ladakh Range, northern India. *Geol. Soc. Am. Bull.* **118**, 383–392 (2006).

50. Stauch, G. & Lehmkuhl, F. Quaternary glaciations in the Verkhoyansk Mountains, Northeast Siberia. *Quat. Res.* **74**, 145–155 (2010).

51. Ward, B. C., Bond, J. D. & Gosse, J. C. Evidence for a 55-50 ka (early Wisconsin) glaciation of the Cordilleran Ice Sheet, Yukon Territory, Canada. *Quat. Res.* **68**, 141–150 (2007).

52. Astakhov, V. I. Late Quaternary glaciation of the northern Urals: a review and new observations. *Boreas* **47**, 379–389 (2018).

53. Astakhov, V., Shkatova, V., Zastrozhnov, A. & Chuyko, M. Glaciomorphological Map of the Russian Federation. *Quat. Int*. **420**, 4–14 (2016).

54. Carr, S. R., Holmes, R., van der Meer, J. J. M. & Rose, J. The Last Glacial Maximum in the North Sea Basin: micromorphological evidence of extensive glaciation. *J. Quat. Sci*. **21**, 131–153 (2006).

55. Glushkova, O. Y. Late Pleistocene glaciations in North-East Asia. In: Ehlers, J., Gibbard, P. L. & Hughes, P. D. (eds). Quaternary Glaciation Extent and Chronology: a closer look. *Developments in Quaternary Science* **15**, Elsevier, Amsterdam (2011).

56. Hjort, C. A glacial chronology for northern East Greenland. *Boreas* **10**, 259-274 (1981).

57. Kaufman, D. S., Young, N. E., Briner, J. P. & Manley, W. F. Alaska palaeo-glacier atlas (version 2). In: Ehlers, J., Gibbard, P. L. & Hughes, P. D. (eds). Quaternary Glaciation Extent and Chronology: a closer look. *Developments in Quaternary Science* **15**, Elsevier, Amsterdam (2011).

58. Kleman, J., Fastook, J., Ebert, K., Nilsson, J. & Caballero, R. Pre-LGM NH ice sheet topography. *Clim. Past* **9**, 2365–2378 (2013).

59. Lundqvist, J. Glacial history of Sweden. In: Ehlers, J. & Gibbard, P. L. (eds.) Quaternary Glaciations – Extent and Chronology, Volume 2. *Developments in Quaternary Science* **2**, Elsevier, Amsterdam (2004).

60. Möller, P., Alexanderson, H., Funder, S. & Hjort, C. The Taimyr Peninsula and the Severnaya Zemlya archipelago, Arctic Russia: a synthesis of glacial history and palaeo-environmental change during the Last Glacial cycle (MIS 5e-2). *Quat. Sci. Rev.* **107**, 149–181 (2015).

61. Rolfe, C. J., Hughes, P. H., Fenton, C. R., Schnabel, C., Xu, S. & Brown, A. G. Paired ^{26}Al and ^{10}Be exposure ages from Lundy: new evidence for the extent and timing of Devensian glaciation in the southern British Isles. *Quat. Sci. Rev.* **43**, 61–73 (2012).

62. Svendsen, J. I. *et al*. Late Quaternary ice sheet history of northern Eurasia. *Quat. Sci. Rev.* **23**, 1229–1271 (2004).

63. Svendsen, J. I., Krüger, L. C., Mangerud, J., Astakhov, V. I., Paus, A., Nazarov, D. & Murray, A. Glacial and vegetation history of the Polar Ural Mountains in northern Russia during the Last Ice Age, Marine Isotope Stages 5-2. *Quat. Sci. Rev.* **92**, 409–428 (2014).

64. Turner, D. G., Ward, B. C., Froese, D. G., Lamothe, M., Bond, J. D. & Bigelow, N. H. Stratigraphy of Pleistocene glaciations in the St Elias Mountains, southwest Yukon, Canada. *Boreas* **45**, 521–536 (2016).

65. Kleman, J., Fastook, J. & Stroeven, A. P. Geologically and geomorphologically constrained numerical model of Laurentide Ice Sheet inception and build-up. *Quat. Int.* **95–96**, 87–98 (2002).

66. Davies, B. J. British and Fennoscandian Ice-Sheet interactions during the Quaternary. PhD thesis, University of Durham (2008).

67. Eccleshall, S. V., Hormes, A., Hovland, A. & Preusser, F. Constraining the chronology of Pleistocene glaciations on Svalbard: Kapp Ekholm re-visited. *Boreas* **45**, 790–803 (2016).

68. Grin, E., Ehlers, T. A., Schaller, M., Sulaymonova, V., Ratschbacher, L. & Gloaguen, R. ^{10}Be surface-exposure age dating of the Last Glacial Maximum in the northern Pamir (Tajikistan). *Quat. Geochronol.* **34**, 47–57 (2016).

69. Hall, R. D. & Shroba, R. R. Soil evidence for a Glaciation Intermediate between the Bull Lake and Pinedale Glaciations at Fremont Lake, Wind River Range, Wyoming, U.S.A. *Arct. Antarct. Alp. Res.* **27**, 89–98 (1995).

70. Sejrup, H. P., Larsen, E., Landvik, J., King, E. L., Haflidason, H., Nesje, A. Quaternary glaciations in southern Fennoscandia: evidence from southwestern Norway and the northern North Sea region. *Quat. Sci. Rev.* **19**, 667–685 (2000).

71. Sejrup, H. P. *et al*. Pleistocene glacial history of the NW European continental margin. *Mar. Petrol. Geol*. **22**, 1111–1129 (2005).

72. Stewart, M. A. & Lonergan, L. Seven glacial cycles in the middle-late Pleistocene of northwest Europe: Geomorphic evidence from buried tunnel valleys. *Geology* **39**, 283–286 (2011).

73. Winkelmann, D., Schafer, C., Stein, R. & Mackensen, A. Terrigenous events and climate history of the Sophia Basin, Arctic Ocean. *Geochem. Geophys. Geosyst.* **9**, Q07023 (2008).

74. Zech, W., Zech, R., Zech, M., Leiber, K., Dippold, M., Frechen, M., Bussert, R. & Andreev, A. Obliquity forcing of Quaternary glaciation and environmental changes in NE Siberia. *Quat. Int.* **234**, 133–145 (2011).

75. Zech, R., Röhringer, I., Sosin, P., Kabgov, H., Merchel, S., Akhmadaliev, S. & Zech, W. Late Pleistocene glaciations in the Gissar Range, Tajikistan, based on ^{10}Be surface exposure dating. *Palaeogeogr., Palaeoclimatol., Palaeoecol.* **369**, 253–261 (2013).

76. Lambeck, K., Purcell, A., Funder, S., Kjær, K. H., Larsen, E. & Möller, P. Constraints on the Late Saalian to early Middle Weichselian ice sheet of Eurasia from field data and rebound modelling. *Boreas* **35**, 539–575 (2006).

77. Blomdin, R. *et al*. Evaluating the timing of former glacier expansions in the Tian Shan: A key step towards robust spatial correlations. *Quat. Sci. Rev*. **153**, 78–96 (2016).

78. Fu, P., Stroeven, A. P., Harbor, J. M., Hättestrand, C., Heyman, J., Caffee, M. W. & Zhou, L. Paleoglaciation of Shaluli Shan, southeastern Tibetan Plateau. *Quat. Sci. Rev*. **64**, 121–135 (2013).

79. Lisiecki, L. E. & Raymo, M. E. A Pliocene-Pleistocene stack of 57 globally distributed benthic δ^{18}O records. *Paleoceanography and Paleoclimatology* **20**, PA1003 (2005).

80. Zhao, J., Wang, J., Harbor, J. M., Liu, S., Yin, X. & Wu, Y. Quaternary glaciations and glacial landform evolution in the Tailan River valley, Tianshan Range, China. *Quat. Int.* **358**, 2–11 (2015).

81. Astakhov, V. Middle Pleistocene glaciations of the Russian North. *Quat. Sci. Rev.* **23**, 1285–1311 (2004).

82. Funder, S., Hjort, C., Landvik, J. Y., Nam, S-I., Reeh, N. & Stein, R. History of a stable ice margin – East Greenland during the Middle and Upper Pleistocene. *Quat. Sci. Rev.* **17**, 77–123 (1998).

83. Chadwick, O. A., Hall, R. D. & Phillips, F. M. Chronology of Pleistocene glacial advances in the central Rocky Mountains. *Geol. Soc. Am. Bull.* **109**, 1143–1452 (1997).

84. Funder, S. The Baffin Bay Region During the Last Interglaciation: Evidence from Northwest Greenland. *Géographie physique at Quaternaire* **43**, 255–262 (1989).

85. Karabanov, E. B., Prokopenko, A. A., Williams, D. F. & Colman, S. M. Evidence from Lake Baikal for Siberian Glaciation during Oxygen-Isotope Substage 5d. *Quat. Res.* **50**, 46–55 (1998).

86. Phillips, F. M., Zreda, M. G., Gosse, J. C., Klein, J., Evenson, E. B., Hall, R. D., Chadwick, O. A. & Sharma, P. Cosmogenic ^{36}Cl and ^{10}Be ages of Quaternary glacial and fluvial deposits of the Wind River Range, Wyoming. *Geol. Soc. Am. Bull.* **109**, 1453–1463 (1997).

87. Balco, G. & Rovey II, C. W. Absolute chronology for major Pleistocene advances of the Laurentide Ice Sheet. *Geology* **38**, 795–798 (2010).

88. Barendregt, R. W., Andriashek, L. & Jackson, L. E. Evidence for Early Pleistocene glaciation obtained from borecores collected in East-Central Alberta, Canada. *American Geophysical Union Meeting*, GP13A-3574, San Francisco, USA (2014).

89. Basilian, A. C., Nikolskiy, P. A. & Anisimov, M. A. Pleistocene glaciation of the New Siberian Islands - no more doubt. *IPY News* **12**, 7–9 (2008).

90. Böse, M., Lüthgens, C., Lee, J. R. & Rose, J. Quaternary glaciations of northern Europe. *Quat. Sci. Rev.* **44**, 1–25 (2012).

91. Curry, B. B., Grimley, D. A. & McKay III, E. D. Quaternary Glaciations in Illinois. In: Ehlers, J., Gibbard, P. L. & Hughes, P. D. (eds). Quaternary Glaciation Extent and Chronology: a closer look. *Developments in Quaternary Science* **15,** Elsevier, Amsterdam (2011).

92. Ehlers, J. Reconstructing the dynamics of the North-west European Pleistocene ice sheets. *Quat. Sci. Rev.* **9**, 71–83 (1990).

93. Ehlers, J., Gibbard, P. L. & Hughes, P. D. (eds). Quaternary Glaciation Extent and Chronology: a closer look. *Developments in Quaternary Science* **15**, Elsevier, Amsterdam (2011).

94. Eissmann, L. Quaternary geology of eastern Germany (Saxony, Saxon-Anhalt, South Brandenburg, Thuringia), type area of the Elsterian and Saalian Stages in Europe. *Quat. Sci. Rev.* **21**, 1275–1346 (2002).

95. Gibbard, P. L. & Clark, C. D. Pleistocene glaciation limits in Great Britain. In: Ehlers, J., Gibbard, P. L. & Hughes, P. D. (eds). Quaternary Glaciation Extent and Chronology: a closer look. *Developments in Quaternary Science* **15**, Elsevier, Amsterdam (2011).

96. Gozhik, P., Lindner, L. & Marks, L. Late Early and early Middle Pleistocene limits of Scandinavian glaciation in Poland and Ukraine. *Quat. Int.* **271**, 31–37 (2010).

97. Hamblin, R. J. O., Moorlock, B. S. P., Rose, J., Lee, J. R., Riding, J. B., Booth, S. J. & Pawley, S. M. Revised Pre-Devensian glacial stratigraphy in Norfolk, England, based on mapping and till provenance. *Neth. J. Geosci.* **84**, 77–85 (2005).

98. Hughes, P. D. & Gibbard, P. L. Global glacier dynamics during 100 ka Pleistocene glacial cycles. *Quat. Res.* **90**, 222–243 (2018).

99. Jackson, L. E., Andriashek, L. D. & Phillips, F. M. Limits of successive Middle and Late Pleistocene continental ice sheets, interior plains of southern and central Alberta and adjacent areas. In: Ehlers, J., Gibbard, P. L. & Hughes, P. D. (eds). Quaternary Glaciation Extent and Chronology: a closer look. *Developments in Quaternary Science* **15**, Elsevier, Amsterdam (2011).

100. Jakobsson, M., Polyak, L., Edwards, M., Kleman, J. & Coakley, B. Glacial geomorphology of the Central Arctic Ocean: the Chukchi Borderland and the Lomonosov Ridge. *Earth Surf. Process. Landforms* **33**, 526–545 (2008).

101. Laban, C. & van der Meer, J. J. M. Pleistocene Glaciation in The Netherlands. In: Ehlers, J., Gibbard, P. L. & Hughes, P. D. (eds). Quaternary Glaciation Extent and Chronology: a closer look. *Developments in Quaternary Science* **15**, Elsevier, Amsterdam (2011).

102. Marks, L. Pleistocene glacial limits in the territory of Poland. *Przegląd Geologiczny* **53**, 988–993 (2005).

103. Marks, L. Quaternary Glaciations in Poland. In: Ehlers, J., Gibbard, P. L. & Hughes, P. D. (eds). Quaternary Glaciation Extent and Chronology: a closer look. *Developments in Quaternary Science* **15**, Elsevier, Amsterdam (2011).

104. Marks, L. *et al*. Revised limit of the Saalian ice sheet in central Europe. *Quat. Int.* **478**, 59–74 (2018).

105. Niessen, F., Hong, J. K., Hegewald, A., Matthiessen, J., Stein, R., Kim, H., Kim, S., Jensen, L., Jokat, W., Nam, S-I. & Kang, S-H. Repeated Pleistocene glaciation of the East Siberian continental margin. *Nat. Geosci.* **6**, 842–846 (2013).

106. Roskosch, J., Winsemann, J., Polom, U., Brandes, C., Tsukamoto, S., Weitkamp, A., Bartholomäus, W. A., Henningsen, D. & Frechen, M. Luminescence dating of ice-marginal deposits in northern Germany: evidence for repeated glaciations during the Middle Pleistocene (MIS 12 to MIS 6). *Boreas* **44**, 103–126 (2015).

107. Colleoni, F., Wekerle, C., Näslund, J.-O., Brandefelt, J. & Masina, J. Constraint on the penultimate glacial maximum NH ice topography (= 140 kyrs BP). *Quat. Sci. Rev*. **137**, 97–112 (2016).

108. Peltier, W. R. Global glacial isostacy and the surface of the ice-age Earth: The ICE-5G (VM2) model and GRACE. *Annu. Rev. Earth Planet. Sci.* **32**, 111–149 (2004).

109. Anderson, R. S., Dühnforth, M., Colgan, W. & Anderson, L. Far-flung moraines: Exploring the feedback of glacial erosion on the evolution of glacier length. *Geomorphology* **179**, 269–285 (2012).

110. Dahlgren, K. I. T., Vorren, T. O. & Laberg, J. S. Late Quaternary glacial development of the mid-Norwegian margin – 65 to 68°N. *Mar. Pet. Geol.* **19**, 1089–1113 (2002).

111. Geirsdóttir, Á., Miller, G. H. & Andrews, J. T. Glaciation, erosion and landscape evolution of Iceland. *J. Geodyn*. **43**, 170–186 (2007).

112. Hjelstuen, B. O., Sejrup, H. P., Haflidason, H., Nygård, A., Ceramicola, S. & Bryn, P. Late Cenozoic glacial history and evolution of the Storegga Slide area and adjacent slide flank regions, Norwegian continental margin. In: Solheim, A., Bryn, P., Berg, K., Sejrup, H. P. & Mienert, J. *Ormen Lange - an Integrated Study for Safe Field Development in the Storegga Submarine Area*, 57–69 (2005).

113. Kuhle, M. The Pleistocene Glaciation (LGP and pre-LGP, Pre-LGM) of SE Iranian Mountains exemplified by the Kuh-i-Jupar, Kuh-i-Lalezar and Kuh-i-Hezar massifs in the Zagros. *Polarforschung* **77**, 71–88 (2007).

114. Licciardi, J. M. & Pierce, K. L. Cosmogenic exposure-age chronologies of Pinedale and Bull Lake glaciations in greater Yellowstone and the Teton Range, USA. *Quat. Sci. Rev.* **27**, 814–831 (2008).

115. Montelli, A., Dowdeswell, J. A., Ottesen, D. & Johansen, S. E. Ice-sheet dynamics through the Quaternary on the mid-Norwegian continental margin inferred from 3D seismic data. *Mar. Pet. Geol.* **80**, 228–242 (2017).

116. Nielsen, T. & Kuijpers, A. Only 5 southern Greenland shelf edge glaciations since the early Pliocene. *Sci. Rep.* **3**, 1875 (2013).

117. Nikolskiy, P. A., Basilyan, A. E. & Zazhigin, V. S. New data on the age of the glaciation in the New Siberian Islands (Russian Eastern Arctic). *Dokl. Earth Sci.* **475**, 748–752 (2017).

118. O'Regan, M. *et al*. The De Long Trough: a newly discovered glacial trough on the East Siberian continental margin. *Clim. Past* **13**, 1269–1284 (2017).

119. Strunk, A., Knudsen, M. F., Egholm, D. L., Jansen, J. D., Levy, L. B., Jacobsen, B. H. & Larsen, N. K. One million years of glaciation and denudation history in west Greenland. *Nat. Commun.* **8**, 14199 (2017).

120. Vorren, T. O. & Laberg, J. S. Trough mouth fans – palaeoclimate and ice-sheet monitors. *Quat. Sci. Rev.* **16**, 865–881 (1997).

121. White, T. S., Bridgland, D. R., Westaway, R., Howard, A. J. & White, M. J. Evidence from the Trent terrace archive, Lincolnshire, UK, for lowland glaciation of Britain during the Middle and Late Pleistocene. *P. Geologist. Assoc.* **121**, 141–153 (2010).

122. White, T. S., Bridgland, D. R., Westaway, R. & Straw, A. Evidence for late Middle Pleistocene glaciation of the British margin of the southern North Sea. *J. Quat. Sci.* **32**, 261–275 (2017).

123. Beets, D. J., Meijer, T., Beets, C. J., Cleveringa, P., Laban, C. & van der Spek, A. J. F. Evidence for a Middle Pleistocene glaciation of MIS 8 age in the southern North Sea. *Quat. Int.* **133-134**, 7–19 (2005).

124. Hodell, D. A., Channell, J. E. T., Curtis, J. H., Romero, O. E. & Röhl, U. Onset of 'Hudson Strait' Heinrich events in the eastern North Atlantic at the end of the middle Pleistocene transition (~640 ka)? *Paleoceanography* **23**, PA4218 (2008).

125. Krissek, L. A. Late Cenozoic ice-rafting records from Leg 145 sites in the North Pacific: Late Miocene onset, Late Pliocene intensification, and Pliocene-Pleistocene events. In: Rea, D. K., Basov, I. A., Scholl, D. W. & Allan, J. F. (Eds.). *Proceedings of the Ocean Drilling Program, Scientific Results* **145** (1995).

126. Spooner, I. S., Osborn, G. D., Barendregt, H. & Irving, E. A Middle Pleistocene (isotope stage 10) glacial sequence in the Stikine River valley, British Columbia. *Can. J. Earth Sci.* **33**, 1428–1438 (1996).

127. Krzyszkowski, D., Wachecka-Kotkowska, L., Wieczorek, D. & Stoiński, A. Petrography of glacial tills in the Szczerców outcrop, central Poland – problems of stratigraphic interpretation. *Studia Quaternaria* **32**, 99–108 (2015).

128. Aber, J. S. The glaciation of northeastern Kansas. *Boreas* **20**, 297–314 (1991).

129. Colgan, P. M. Early middle Pleistocene glacial sediments (780000-620000 BP) near Kansas City, northeastern Kansas and northwestern Missouri, USA. *Boreas* **28**, 477–489 (1999).

130. Toucanne, S. *et al*. A 1.2 Ma record of glaciation and fluvial discharge from the West European Atlantic margin. *Quat. Sci. Rev* **28**, 2974–2981 (2009).

131. Andriashek, L. D. & Barendregt, R. W. Evidence for Early Pleistocene glaciation from borecore stratigraphy in north-central Alberta, Canada. *Can. J. Earth Sci.* **54**, 445–460 (2017).

132. Batchelor, C. L., Ottesen, D. & Dowdeswell, J. A. Quaternary evolution of the northern North Sea margin through glacigenic debris-flow and contourite deposition. *J. Quat. Sci.* **32**, 416–426 (2017).

133. Ottesen, D., Batchelor, C. L., Dowdeswell, J. A. & Løseth, H. Morphology and pattern of Quaternary sedimentation in the North Sea Basin (52-62°N). *Mar. Petrol. Geol.* **98**, 836–859 (2018).

134. Bierman, P. R., Shakun, J. D., Corbett, L. B., Zimmerman, S. R. & Rood, D. H. A persistent and dynamic East Greenland Ice Sheet over the past 7.5 million years. *Nature* **540**, 256–260 (2016).

135. Laberg, J. S., Forwick, M., Husum, K. & Nielsen, T. A re-evaluation of the Pleistocene behaviour of the Scoresby Sund sector of the Greenland Ice Sheet. *Geology* **41**, 1231–1234 (2013).

136. Sejrup, H. P., Aarseth, I. & Haflidason, H. The Quaternary succession in the northern North Sea. *Mar. Geol.* **101**, 103–111 (1991).

137. Thierens, M. *et al*. Ice-rafting from the British-Irish ice sheet since the earliest Pleistocene (2.6 million years ago): Implications for long-term mid-latitudinal ice-sheet growth in the North Atlantic region. *Quat. Sci. Rev*. **44**, 229–240 (2012).

138. Barendregt, R. W. & Duk-Rodkin, A. Chronology and extent of late Cenozoic ice sheets in North America: a magnetostratigraphic assessment. In: Ehlers, J., Gibbard P.L. & Hughes P.D. (eds.), *Quaternary Glaciations - Extent and Chronology, Part IV: a closer look.* Developments in Quaternary Science **15**, Elsevier, Amsterdam (2011).

139. Dowdeswell, J. A. & Ottesen, D. Buried iceberg ploughmarks in the early Quaternary sediments of the central North Sea: a two-million-year record of glacial influence from 3D seismic data. *Mar. Geol.* **344**, 1–9 (2013).

140. Kleman, J., Stroeven, A. P. & Lundqvist, J. Patterns of Quaternary ice sheet erosion and deposition in Fennoscandia and a theoretical framework for explanation. *Geomorphology* **97**, 73–90 (2008).

141. Knies, J. *et al*. The Plio-Pleistocene glaciation of the Barents Sea-Svalbard region: a new model based on revised chronostratigraphy. *Quat. Sci. Rev*. **28**, 812–829 (2009).

142. Rea, B. R. *et al*. Extensive marine-terminating ice sheets in Europe from 2.5 million years ago. *Sci. Adv.* **4**, eaar8327 (2018).

143. Solgaard, A. M., Reeh, N., Japsen, P., Nielsen, T. Snapshots of the Greenland ice sheet configuration in the Pliocene to early Pleistocene. *J. Glaciol*. **57**, 871–880 (2011).

144. Bailey, I., Hole, G. M., Foster, G. L., Wilson, P. A., Storey, C. D., Trueman, C. N. & Raymo, M. E. An alternative suggestion for the Pliocene onset of major northern hemisphere glaciation based on the geochemical provenance of North Atlantic Ocean ice-rafted debris. *Quat. Sci. Rev*. **75**, 181–194 (2013).

145. Berger, D. & Jokat, W. Sediment deposition in the northern basins of the North Atlantic and characteristic variations in shelf sedimentation along the East Greenland margin. *Mar. Pet. Geol.* **26**, 1321–1337 (2009).

146. Butt, F. A., Elverhøi, A., Forsberg, C. F. & Solheim A. Evolution of the Scoresby Sund Fan, central East Greenland- evidence from ODP Site 987. *Norsk Geologisk Tidsskrift* **81**, 3–15 (2001).

147. Hidy, A. J., Gosse, J. C., Froese, D. G., Bond, J. D., Rood, D. H. A latest Pliocene age for the earliest and most extensive Cordilleran Ice Sheet in northwest Canada. *Quat. Sci. Rev.* **61**, 77–84 (2013).

148. Hofmann, J. C., Knutz, P. C., Nielsen, T. & Kuijpers, A. Seismic architecture and evolution of the Disko Bay trough-mouth fan, central West Greenland margin. *Quat. Sci. Rev.* **147**, 69–90 (2016).

149. Jansen, E., Fronval, T., Rack, F. & Channel, J. E. T. Pliocene-Pleistocene ice rafting history and cyclicity in the Nordic Seas during the last 3.5 Myr. *Paleoceanogr. Paleoclimatol.* **15**, 709–721 (2000).

150. Solheim, A., Faleide, J. I., Andersen, E. S., Elverhøi, A., Forsberg, C. F., Vanneste, K., Uenzelmann-Neben, G., Channell, J. E. T. Late Cenozoic seismic stratigraphy

and glacial geological development of the East Greenland and Svalbard-Barents Sea continental margins. *Quat. Sci. Rev.* **17**, 155–184 (1998).

151. Evans, J., Ó Cofaigh, C., Dowdeswell, J. A. & Wadhams, P. Marine geophysical evidence for former expansion and flow of the Greenland Ice Sheet across the north-east Greenland continental shelf. *J. Quat. Sci*. **24**, 279–293 (2009).

152. Dowdeswell, J. A., Evans, J. & Ó Cofaigh, C. Submarine landforms and shallow acoustic stratigraphy of a 400 km-long fjord-shelf-slope transect, Kangerlussuaq margin, East Greenland. *Quat. Sci. Rev.* **29**, 3359–3369 (2010).

153. Ó Cofaigh, C. *et al*. An extensive and dynamic ice sheet on the West Greenland shelf during the last glacial cycle. *Geology* **41**, 219–222 (2013).

154. Shaw, J., Piper, D. J. W., Fader, G. B. J., King, E. L., Todd, B. J., Bell, T., Batterson, M. J. & Liverman, D. G. E. A conceptual model of the deglaciation of Atlantic Canada. *Quat. Sci. Rev.* **25**, 2059–2081 (2006).

155. Dyke, A. S., Moore, A. & Robertson, L. Deglaciation of North America, Scale 1:7000000. Geological Survey of Canada, Open File 1574 (2003).

156. Barr, I. D. & Clark, C. D. Late Quaternary glaciations in Far NE Russia; combining moraines, topography and chronology to assess regional and global glaciation synchrony. *Quat. Sci. Rev*. **53**, 72–87 (2012).

157. Berger, G. W. & Nielsen, E. Evidence from thermoluminescence dating for Middle Wisconsinan deglaciation in the Hudson Bay Lowland of Manitoba. *Can. J. Earth Sci*. **28**, 240–249 (1991).

158. Larsen, E., Sejrup, H. P., Janocko, J., Landvik, J. Y., Stalsberg, K. & Steinsund, P. I. Recurrent interaction between the Norwegian Channel Ice Stream and terrestrial-based ice across southwest Norway. *Boreas* **29**, 185–203 (2000).

159. Dalton, A. S., Finkelstein, S. A., Barnett, P. J. & Forman, S. L. Constraining the Late Pleistocene history of the Laurentide Ice Sheet by dating the Missinaibi Formation, Hudson Bay Lowlands, Canada. *Quat. Sci. Rev.* **146**, 288–299 (2016).

160. Young, R. R., Burns, J. A., Smith, D. G., Arnold, L. D. & Rains, R. B. A single, late Wisconsin, Laurentide glaciation, Edmonton area and southwestern Alberta. *Geology* **22**, 683–686 (1994).

161. Guyard, H. *et al*. New insights into Late Pleistocene glacial and postglacial history of northernmost Ungava (Canada) from Pingualuit Crater Lake sediments. *Quat. Sci. Rev.* **30**, 3892–3907 (2011).

162. Carlson, A. E., Tarasov, L. & Pico, T. Rapid Laurentide ice-sheet advance towards southern last glacial maximum limit during marine isotope stage 3. *Quat. Sci. Rev.* **196**, 118–123 (2018).

163. Pico, T., Mitrovica, J. X., Braun, J. & Ferrier, K. L. Glacial isostatic adjustment deflects the path of the ancestral Hudson River. *Geology* **46**, 591–594 (2018).

164. Mangerud, J., Løvlie, R., Gulliksen, S., Hufthammer, K-A., Larsen E. & Valen, V. Paleomagnetic correlations between Scandinavian Ice-Sheet fluctuations and Greenland Dansgaard-Oeschger events, 45,000-25,000 yr B.P. *Quat. Res.* **59**, 213–222 (2003).

165. Sejrup, H. P. *et al*. Configuration, history and impact of the Norwegian Channel Ice Stream. *Boreas* **32**, 18–36 (2003).

166. Dalton, A. S., Finkelstein, S. A., Forman, S. L., Barnett, P. J., Pico, T, & Mitrovica, J. X. Was the Laurentide Ice Sheet significantly reduced during Marine Isotope Stage 3? *Geology* **47**, 111–114 (2019).

167. McMartin, I., Campbell, J. E. & Dredge, L. A. Middle Wisconsinan marine shells near Repulse Bay, Nunavut, Canada: implications for Marine Isotope Stage 3 ice-free conditions and Laurentide Ice Sheet dynamics in north-west Hudson Bay. *J. Quat. Sci*. **34**, 64–75 (2019).

168. Johansson, P., Lunkka, J. P. & Sarala, P. The glaciation of Finland. In: Ehlers, J., Gibbard, P. L. & Hughes, P. D. (eds). Quaternary Glaciation Extent and Chronology: a closer look. *Developments in Quaternary Science* **15**, Elsevier, Amsterdam (2011).

169. Lamothe, M. Apparent thermoluminescence ages of St-Pierre sediments at Pierreville, Quebec, and the problem of anomalous fading. *Can. J. Earth Sci.* **21**, 1406–1409 (1984).

170. de Vernal, A., Causse, C., Hillaire-Marcel, C., Mott, R. J. & Occhietti, S. Palynostratigraphy and Th/U ages of upper Pleistocene interglacial and interstadial deposits on Cape Breton Island, eastern Canada. *Geology* **14**, 554–557 (1986).

171. Berger, G. W. & Eyles, N. Thermoluminescence chronology of Toronto-area Quaternary sediments and implications for the extent of the midcontinent ice sheet(s). *Geology* **22**, 31–34 (1994).

172. Karrow, P. F., Dreimanis, A. & Barnett, P. J. A proposed diachronic revision of Late Quaternary time-stratigraphic classification in the eastern and northern Great Lakes area. *Quat. Res.* **54**, 1–12 (2000).

173. Munroe, J. S., Perzan, Z. M. & Amidon, W. H. Cave sediments constrain the latest Pleistocene advance of the Laurentide Ice Sheet in the Champlain Valley, Vermont, USA. *J. Quat. Sci*. **31**, 893–904 (2016).

174. Rémillard, A. M., St-Onge, G., Bernatchez, P., Hétu, B., Buylaert, J-P., Murray, A. S. & Lajeunesse, P. Relative sea-level changes and glacio-isostatic adjustment on the Magdalen Islands archipelago (Atlantic Canada) from MIS 5 to the late Holocene. *Quat. Sci. Rev.* **171**, 216–233 (2017).

175. Allard, G., Roy, M., Ghaleb, B., Richard, P. J. H., Larouche, A. C., Veillette, J. J. & Parent, M. Constraining the age of the last interglacial-glacial transition in the Hudson Bay lowlands (Canada) using U-Th dating or buried wood. *Quat. Geochronol*. **7**, 37–47 (2012).

176. Dalton, A. S, Finkelstein, S. A., Barnett, P. J., Väliranta, M. & Forman, S. L. Late Pleistocene chronology, palaeoecology and stratigraphy at a suite of sites along the Albany River, Hudson Bay Lowlands, Canada. *Palaeogeogr., Palaeoclimatol., Palaeoecol* **492**, 50–63 (2018).

177. Ehlers, J. & Gibbard, P. L. Quaternary Glaciations – Extent and Chronology. *Developments in Quaternary Science* **2**. Elsevier, Amsterdam (2004).

178. Dorale, J. A., Onac, B. P., Fornós, J. J., Ginés, J., Ginés, A., Tuccimei, P. & Peate, D. W. Sea-level highstand 81,000 years ago in Mallorca. *Science* **327**, 860–863 (2010).

179. Batchelor, C. L., Dowdeswell, J. A. & Pietras, J. T. Variable history of Quaternary ice-sheet advance across the Beaufort Sea margin, Arctic Ocean. *Geology* **41**, 131–134 (2013).

180. Dubé-Loubert, H., Roy, M., Allard, G., Lamothe, M. & Veillette, J. J. Glacial and nonglacial events in the eastern James Bay lowlands, Canada. *Can. J. Earth Sci.* **50**, 379–396 (2013).

181. Arkhipov, S. A. Pleistocene chronostratigrapy in northern Siberia. Proceedings of the First International Colloqium on Quaternary stratigraphy of Asia and Pacific Area, Osaka, 163–177 (1987).

182. Arkhipov, S. A. Chronostratigraphy of Pleistocene in northern Siberia. *Russ. Geol. Geophys.* **6**, 13–21 (1989).

183. Jakobsson, M. *et al*. Evidence for an ice shelf covering the central Arctic Ocean during the penultimate glaciation. *Nat. Commun.* **7**, 10365 (2016).

184. Huscroft, C. A., Ward, B. C., Barendregt, R. W., Jackson, L. E. & Opdyke, N. D. Pleistocene volcanic damming of Yukon River and the maximum age of the Reid Glaciation, west-central Yukon. *Can. J. Earth Sci.* **41**, 151–164 (2004).

185. Lindner, L., Bogucki, A., Chlebowski, R. & Gożik, P. The importance of glacial till occurrence in loess type sections of Poland and Ukraine. *Przegląd Geologiczny* **52**, 331–335 (2004).

186. Shepps, V. C., White, G. W., Droste, J. B. & Sitler, R. F. Glacial geology of northwestern Pennsylvania. *Pennsylvania Geological Survey, 4th Series, Bulletin G* **32**, 59 (1959).

187. White, G. W. Pleistocene deposits of the north-western Allegheny Plateau, USA. *Quaterly Jour. Geo. Soc. London* **124**, 131–151 (1968).

188. Marchand, D., Ciolkosz, E. J., Bucek, M. E. & Crowl, G. H. *Quaternary Deposits and Soils of the Central Susquehanna Valley of Pennsylvania*. Agronomy Department, Pennsylvania State University, University Park (1978).

189. Braun, A., Kuo, C-Y., Shum, C. K., Wu, P., van der Wal, W. & Fotopoulos, G. Glacial isostatic adjustment at the Laurentide ice sheet margin: Models and observations in the Great Lake region. *J. Geodyn*. **46**, 165–173 (2008).

190. Braun, D. D. The glaciation of Pennsylvania, USA. In: Ehlers, J., Gibbard, P. L. & Hughes, P. D. (eds). Quaternary Glaciation Extent and Chronology: a closer look. *Developments in Quaternary Science* **15,** Elsevier, Amsterdam (2011).

191. Gibbard, P. L. The history of the great northwest European rivers during the past three million years. *Phil. Trans. Royal Soc. London* **318**, 559–602 (1988).

192. Cohen, K. M., Gibbard, P. L. & Weerts, H. J. T. North Sea palaeogeographical reconstructions for the last 1 Ma. *Neth. J. Geosci.* **93**, 7–29 (2014).

193. Sejrup, H. P., Aarseth, I., Haflidason, H., Løvlie, R., Bratten, Å., Tjøstheim, G. & Forsberg, C. F. Quaternary of the Norwegian Channel; paleoceanography and glaciation history. *Nor. Geol. Tidsskr.* **75**, 65–87 (1995).

194. Reinardy, B. T. I., Hjelstuen, B. O., Sejrup, H. P., Augedal, H. & Jørstad, A. Late Pliocene-Pleistocene environments and glacial history of the northern North Sea. *Quat. Sci. Rev*. **158**, 107–126 (2017).

195. Faleide, J. I., Solheim, A., Fiedler, A., Hjelstuen, B. O., Andersen, E. S. & Vanneste, K. Late Cenozoic evolution of the western Barents Sea-Svalbard continental margin. *Glob. Planet. Change* **12**, 53–74 (1996).

196. Andreassen, K., Nilssen, L. C., Rafaelsen, B. & Kuilman, L. Three-dimensional seismic data from the Barents Sea margin reveal evidence of past ice stream and their dynamics. Geology **32**, 729–732 (2004).

197. Andreassen, K., Glad Nilssen, E. & Ødegård, C.M. Analysis of shallow gas and fluid migration within the Plio-Pleistocene sedimentary succession of the SW Barents Sea continental margin using 3D seismic data. *Geo-Mar. Lett.* **27**, 155–171 (2007).

198. Knutz, P. C., Hopper, J. R., Gregersen, U., Nielsen T. & Japsen, P. A contourite drift system on the Baffin Bay–West Greenland margin linking Pliocene Arctic warming to poleward ocean circulation. *Geology* **43**, 907–910 (2015).

199. Pérez, L. F., Nielsen, T., Knutz, P. C., Kuijpers, A. & Damm, V. Large-scale evolution of the central-east Greenland margin: new insights to the North Atlantic glaciation history. *Glob. Planet. Change* **163**, 141–157 (2018).

200. Funder, S., Bennike, O., Böcher, J., Isrealson, C., Petersen, K. S. & Símonarson, L. A. Late Pliocene Greenland – the Kap København Formation in North Greenland. *Bull. Geol. Soc. Denmark* **48**, 117–134 (2001).

201. Bennike, O., Knudsen, K. L., Abrahamsen, N., Böcher, J., Cremer, H. & Wagner, B. Early Pleistocene sediments on Store Koldewey, northeast Greenland. Boreas 39, 603–619 (2010).

202. Ottesen, D., Rise, L., Andersen, E. S., Bugge, T. & Eidvin, T. Geological evolution of the Norwegian continental shelf between 61°N and 68°N during the last 3 million years. *Nor. J. Geol.* **89**, 251-265 (2009).

203. Ottesen, D., Dowdeswell, J. A. & Bugge, T. Morphology, sedimentary infill and depositional environments of the Early Quaternary North Sea Basin (56°-62°N). *Mar. Petrol. Geol.* **56**, 123–146 (2014).

204. Eidvin, T. & Rundberg, Y. Late Cainozoic stratigraphy of the Tampen area (Snorre and Visund fields) in the northern North Sea, with emphasis on the chronology of early Neogene sands. *Norsk Geologisk Tidsskrift* **81**, 119–160 (2001).

205. Dowsett, H., Thompson, R., Barron, J., Cronin, T., Fleming, F., Ishman, S., Poore, R. Willard, D. & Holtz Jr., T. Joint investigations of the Middle Pliocene climate I: PRISM paleoenvironmental reconstructions. *Glob. Planet. Change* **9,** 169–195 (1994).

206. Salzmann, U., Haywood, A. M., Lunt, D. J. The past is a guide to the future? Comparing Middle Pliocene vegetation with predicted biome distributions for the twenty-first century. *Philos. Trans. Royal Soc. A.* **367** (2008).

207. Haywood, A. M., Chandler, M. A., Valdes, P. J., Salzmann, U., Lunt, D. J. & Dowsett, H. J. Comparison of mid-Pliocene climate predictions produced by the HadAM3 and GCMAM3 General Circulation Models. *Glob. Planet. Change* **66**, 208–224 (2009).

208. McKelvey, B. C., Chen, W. & Arculus, R. J. Provenance of Pliocene-Pleistocene ice-rafted debris, Leg 145, Northern Pacific Ocean. In: D. K. Rea, I. A. Basov, D. W. Scholl and J. F. Allan (Eds.), *Proceedings of the Ocean Drilling Program, Scientific Results* **145**, 195–204 (1995).
